# Reaction-diffusion models in weighted and directed connectomes

**DOI:** 10.1371/journal.pcbi.1010507

**Published:** 2022-10-28

**Authors:** Oliver Schmitt, Christian Nitzsche, Peter Eipert, Vishnu Prathapan, Marc-Thorsten Hütt, Claus C. Hilgetag

**Affiliations:** 1 Medical School Hamburg, University of Applied Sciences, Hamburg; University of Rostock, Department of Anatomy, Rostock, Germany; 2 Jacobs University, Life Sciences & Chemistry, Computational Systems Biology, Bremen, Germany; 3 University of Hamburg, Department of Computational Neuroscience, Hamburg, Germany; University of Nottingham, UNITED KINGDOM

## Abstract

Connectomes represent comprehensive descriptions of neural connections in a nervous system to better understand and model central brain function and peripheral processing of afferent and efferent neural signals. Connectomes can be considered as a distinctive and necessary structural component alongside glial, vascular, neurochemical, and metabolic networks of the nervous systems of higher organisms that are required for the control of body functions and interaction with the environment. They are carriers of functional phenomena such as planning behavior and cognition, which are based on the processing of highly dynamic neural signaling patterns. In this study, we examine more detailed connectomes with edge weighting and orientation properties, in which reciprocal neuronal connections are also considered. Diffusion processes are a further necessary condition for generating dynamic bioelectric patterns in connectomes. Based on our precise connectome data, we investigate different diffusion-reaction models to study the propagation of dynamic concentration patterns in control and lesioned connectomes. Therefore, differential equations for modeling diffusion were combined with well-known reaction terms to allow the use of connection weights, connectivity orientation and spatial distances.

Three reaction-diffusion systems Gray-Scott, Gierer-Meinhardt and Mimura-Murray were investigated. For this purpose, implicit solvers were implemented in a numerically stable reaction-diffusion system within the framework of *neuroVIISAS*. The implemented reaction-diffusion systems were applied to a subconnectome which shapes the mechanosensitive pathway that is strongly affected in the multiple sclerosis demyelination disease. It was found that demyelination modeling by connectivity weight modulation changes the oscillations of the target region, i.e. the primary somatosensory cortex, of the mechanosensitive pathway.

In conclusion, a new application of reaction-diffusion systems to weighted and directed connectomes has been realized. Because the implementation was realized in the *neuroVIISAS* framework many possibilities for the study of dynamic reaction-diffusion processes in empirical connectomes as well as specific randomized network models are available now.

## Author summary

Reaction-diffusion systems were adapted and analyzed in weighted and directed connectomes. The systems were applied to a multiple sclerosis model by modulating connectivity weights within the reaction-diffusion process. This leads to changes in the oscillation patterns of a target region of the mechanosensitive pathway.

## Introduction

Diffusion is the process by which matter or particles like atoms or molecules naturally move from regions where they are highly *concentrated* to regions where they are not as concentrated. We can think of diffusion of different particles as a dynamic process along a line or one dimension. The particles move randomly, which is also called a *random walk*. In view of the numerous realizations of diffusion processes in natural biological systems and compartments, it appears obvious to apply diffusion in neural networks. Models of reaction-diffusion (RD) adapted to networks can be used to investigate the spreading of information through networks or connectomes which may shape dynamic states that can be related to functional processes. It has been shown that dynamic models of brain communication have begun to create links between connectional architectures and function. Furthermore, brains have the capacity to support a great diversity of dynamic patterns which are complex at a broad range of temporal frames to sustain a large number of competing functional demands. A large amount of different dynamic patterns has been considered as a functional repertoire of a network that allows flexibility across a broad range of cognitive functions [1].

### Applications of diffusion

Reaction-diffusion systems produce complex self-organized patterns, such as spreading pulses and fronts, stationary dissipative shapes, rotating waves and turbulences [2–6]. Some reaction-diffusion models have been found to work on networks through interacting species occupying network nodes and diffusively transferred across links [7–11]. Reaction-diffusion models can also correspond to networks of diffusively coupled biological cells or regions and chemical reactors [8–11].

Reaction-diffusion processes can be considered and applied on different scales. On the macro and meso level, the application is suitable for neuronal networks, connectomes and local circuits. Recently, a paper on 3D reaction-diffusion models of neurons and networks was published [12]. At the micro level, reaction-diffusion systems are applied to the modeling of cellular and subcellular processes. Here, NeuroRD (https://github.com/neurord; http://modeldb.science/modellist?id=139757&all_simu=true) should be mentioned, which is used for the simulation of neuronal signaling pathways such as that of stochastic simulation on spiny dendrites [13]. In NeuroRD the Gillespie’s tau-leap reaction algorithm, and the stochastic diffusion algorithm of Blackwell are realized. At the synaptic level [14] and on biological surfaces [15] diffusion modeling is applied, for example, by MCell (https://mcell.org/). In detailed synpase models, diffusion processes are also considered [16, 17]. Beyond MCell, diffusion processes are also used in particularly detailed synpase models [16, 17]. Virtual Cell (VCell) (https://vcell.org/) is a comprehensive platform for modeling cell biological systems based on a central database and distributed as a web application [18, 19]. This environment is also used to model cellular diffusion processes [20, 21] and neuronal signaling [22]. A set of libraries for GENESIS is Chemesis, which allows the simulation of reaction-diffusion systems, including calcium release. This approach has been used by [23] to create in a multicompartment-model of phototransduction, calcium dynamics, and ionic currents of a photoreceptor. We should also mention that there is a wide range of applications for electrodiffusion such as the calculation of extracellular electric potentials from neuron stimulations [24], the simulation of electrodiffusion and water movements in brain tissue [25], ionic electrodiffusion with cortical propagation depression [26], and application in electrodiffusive neuron-extracellular-glia models to study the emergence of slow potentials in the brain [27]. Finally, we must mention here the application of diffusion processes to the modeling of diseases such as strokes [28, 29] and natural developmental processes [30].

In complex networks, the analysis of self-organization is difficult and analysis has been restricted to non-equilibrium pattern formation as synchronization [31–33] or epidemic spreading [34–36]. Turing demonstrated [2] that changes of the diffusion constants of activators and inhibitors produce a destabilization of the uniform state of a system and lead to the spontaneous emergence of oscillatory patterns (Turing patterns) which emerge in chemical reactions, biological morphogenesis, and in ecosystems. This kind of complex non-equilibrium self-organization and Turing instabilities can occur in networks as well [8]. Such network-instabilities have been explored further in mathematical frameworks [9–11]. However, their analyses were restricted to regular lattices [8, 9] and small networks [10, 11]. Recent theoretical work has elucidated the relationship between network architecture and diversity of Turing patterns [37].

Diffusion models have been proven useful for delineating functional modules [38, 39] and predicting statistical dependencies (functional connectivity) among remote neuronal time courses [40, 41]. *Network diffusion* or *graph diffusion* [42] has also been applied successfully to network modularity analysis [43] as well as to modeling the relation between structural and functional brain connectivity networks [44].

Meanwhile, several research groups are engaged in the application of diffusion models in networks of nervous systems [40, 44–46]. In most cases, the above-mentioned particles of diffusion processes are considered as components of information. Diffusion processes can then be extended as a flow-based communication model between areas of a nervous system [47–51]. This type of dynamic model is also suitable to describe the phenomenon of multisensory integration [47] or to model the dynamics of brain diseases [45, 46, 48].

### Diffusion in connectomes

Through the use of different reaction terms in the differential equations, reaction-diffusion models allow a versatile parameterization of the dynamic behavior in coupled systems such as neuronal connectomes. In the present study, we adopted different models such as predator-prey and activator-inhibitor for use in connectomes to have more options available for studying the propagation of diffusion processes in networks.

Analyzing reaction-diffusion models on connectome architectures provides insight in the pattern forming capabilities and, hence, the feasible collective modes, of such architectures. Here we first illustrate, using simple, generic network architectures, how reaction-diffusion systems create sets of nodes with common dynamical behaviors, which cannot be trivially derived from the network architecture alone. We subsequently apply this approach to the spinal cord, brainstem, diencephalic and cortical connectivity of the mechanosensory pathways. This new approach—probing connectomes with reaction-diffusion models—is fully integrated in *neuroVIISAS* [52]. A detailed tutorial is provided as Supporting Information.

For the application of reaction-diffusion models, the weights of neuronal connections of a connectome are considered as strengths of connections between regions. Weights of connections in tract-tracing studies encode the number of nerve fibers that have incorporated a tract-tracing substance or an estimate of the number of traced nerve fibers. Thus, estimates of weights of connections do not describe mean thickness of myelin sheaths which are investigated by transmission electron microscopy or other techniques. In most tract-tracing studies weights or densities of connections are estimates which are described in the three basic categories weak, moderate and strong. In addition, connections may be described without any categories at all or in further categories of weights like “weak to moderate” or “very strong”. A survey of all categories, their interpretation, relations and comparisons are given by Schwanke et al. [53]. In terms of a relation of weights of connections and their functional mean they can be interpreted as the strength of a connection which allows transmission of an electrochemical signal between regions of the nervous system. If the strength of a connection is strong then more information can be transmitted. With regard to neuropathological changes of nerve fibers like those in multiple sclerosis where demyelination and axon degeneration occur, the weights of connection can be related to such pathological processes. In this case the weights of connection will be reduced. If weights of connections are used in this study, the ordinal scaled estimates were always logarithmically transformed.

A generalized form of the predator-prey model of Lotka-Volterra was introduced by Mimura-Murray [6, 54, 55] with some advantages with regard to the original concept. The *Mimura-Murray model* (MM) appears to be promising for adaption to network diffusion.

An activator-inhibitor reaction-diffusion model was developed by *Gierer and Meinhardt* (GM) [56–58]. It consists of a reaction term with activator and inhibitor parameters that could be adopted to generate specific oscillation patterns in weighted digraphs. Therefore, the GM model appears to be an interesting candidate for applying in network diffusion. Finally, the *Gray-Scott model* (GS), which is a classical mathematical model for isothermal autocatalytic reaction with another type of reaction term in the differential equation [59–65], has been adapted to network diffusion.

The objective of this study was to investigate reaction-diffusion models with regard to weighted digraphs and distances in the diffusion terms. Here, data of the connectome of the rat nervous system were used and synthetic randomized directed networks with preserved edges and nodes. The reaction-diffusion models of Gierer-Meinhardt [56–58], Mimura-Murray [6, 54, 55] and Gray-Scott [59–65] were adapted to weighted digraphs under consideration of the estimated or linear Euclidean distances between nodes in order to build more realistic coupled dynamic models based on empirical data.

A second aim of this investigation was the analysis of pattern forming in such a way that the reaction-diffusion models generate sets of nodes with common dynamical behavior.

A further objective of this work was to investigate the effects of changes of connection weights within the reaction-diffusion process because this could be a starting point for modeling progression of neurodegenerative diseases [66–71]. The change in connection weights in parameterized models relates specifically to the class of neurodegenerative diseases found in neuronal connections rather than gray matter or neuronal perikarya. Multiple sclerosis is one of such demyelinating diseases [72–81] with a large variety of temporal and topographic progression patterns. The framework used here allows to define sets of source nodes or regions and sets of target nodes of interest embedded in a large connectome context to systematically investigate signal propagation or information diffusion through highly complex connectional architectures which undergo precisely defined changes of connections weights. Hence, this simulation environment appears to be a promising starting point to investigate in a consistent and reproducible way changes of network features as in neurological disorders and their effect of dynamic pattern modifications.

## Materials and methods

### Connectome data and structure

The connectome data were generated in a metastudy of original research publications. Collating information of neuronal connections between pairs of regions can be performed by manual readout of data in over 7000 original research publications which describe the anterograde and retrograde transport of tract-tracing substances. This metastudy approach is well established and has been successfully performed in ferret, avian, macaque, cat and rat [82–87]. The connectome, circuit and lattice data used in this investigation can be downloaded from [88] and the *neuroVIISAS* framework from https://neuroviisas.med.uni-rostock.de/neuroviisas.shtml. How to install and start *neuroVIISAS* is described in the supplement. The test.brain and MS.brain project files can be directly loaded in *neuroVIISAS*. The reference.bib file (included in [88]) provides the links of connections with original research literature and need to be configured in *neuroVIISAS* [52].

### Implementation of reaction-diffusion models

Starting from the broad application of reaction-diffusion systems presented in the introduction, we would like to investigate the property of pulse propagations in networks. More specifically, the reaction-diffusion models will be applied to weighted and directed connectomes. Since all areas are represented in our connectome data that are also affected in multiple sclerosis diseases, a differential investigation of the reaction-diffusion models of control connectome and in multiple sclerosis lesioned connectome will be performed. Since there are no studies on the propagation of concentrations of the reaction-diffusion models in a directional and weighted connectome, we have implemented three basic models, namely the Gierer-Meinhardt, Mimura-Murray and Gray-Scott models, in the neuroVIISAS framework in such a way that the models are directly applicable with selectable parameters to connectomes with the properties listed above.

In the tutorial part 2 the Gierer-Meinhardt model, as well as the other two models, will be explained in more detail, therefore only the functions of the two substances *U*(*x*, *y*, *t*) and *V*(*x*, *y*, *t*) at node *N*(*x*, *y*) of two-dimensional regular lattice should be shown in the following:

$$\begin{array}{ccc} \frac{dU(t)}{dt} & =\underset{≕f(U,V)}{\underset{︸}{r_{u}\cdot \frac{U^{2}}{(1+\kappa U^{2})V}-\mu_{u}\cdot U+\sigma_{u}}} & +D_{u}\Delta U \end{array}$$ (1)

$$\begin{array}{ccc} \frac{dV(t)}{dt} & =\underset{≕g(U,V)}{\underset{︸}{r_{v}\cdot U^{2}-\mu_{v}\cdot V+\sigma_{v}}} & +D_{v}\Delta V \end{array}$$ (2)

The Laplace operator Δ in the following form

$$\begin{array}{c} \Delta U=\frac{\partial^{2}U}{\partial x^{2}}+\frac{\partial^{2}U}{\partial y^{2}},\;\Delta V=\frac{\partial^{2}V}{\partial x^{2}}+\frac{\partial^{2}V}{\partial y^{2}} \end{array}$$ (3)

allows the diffusion of the two substances to be determined.

The reaction-diffusion models applied to the connectome data are based on differential equation systems which could be stiff and are solved by implicit solvers. Their form, solution and extension with regard to noise, connection weight modulation and Euclidean distances are described in detail in the second part of the Supporting Information (Tutorial part 2).

In tutorial part 2, we describe in detail the motivation for using reaction-diffusion models in directed and weighted connectomes. In particular, the formal implementation of the reaction-diffusion models is discussed in detail. For this purpose we start from the Laplace operator to model spatial diffusion. First, the model is not developed for connectomes but for regular lattices. By means of discretization we obtain a system of ordinary differential equations (ODE). For the solution of the ODEs different methods have been implemented in neuroVIISAS (Euler solver, Runge-Kutta, step-controlled Dormand-Prince).

The three reaction-diffusion models are formally described and the initial values of the parameters of the models with which the diffusion patterns known from the literature can be generated. With our implemented models, the same patterns as the patterns known from the literature have been computed, so that our models provide reproducible results. After the formal introduction of the three models, we describe in the tutorial how they are incorporated in a directed network and what has to be considered to define the diffusion directions in a directed network. For this purpose we have provided a selection option that allows to choose between input diffusion and output diffusion of the adjacency matrix and the adjacency matrix transposition (for details see turtorial part 2).

Since multiple sclerosis is a dynamic disease of the myelin sheaths, the connection weights in the connectome also change dynamically. In the tutorial part 2 we explain how the coupling matrix *L* was temporally modulated to realize the dynamics of demyelination and remyelination within a simulation:

$$\begin{array}{c} \frac{dU}{dt}=f\left(U\right)+L\left(t\right)\cdot U \end{array}$$ (4)

Following the implementing the basic properties of the connectome diffusion models, two methods for generating additive noise and stochastic noise (Ornstein-Uhlenbeck process) were implemented to verify the stability of the reactions-diffusion model with respect to different noise levels. To realize this, a coupled Ornstein-Uhlenbeck process *δ* was applied additively to the directed diffusion. The different steps for implementing this function were also explained in detail in Tutorial Part 2:

$$\begin{array}{c} U(t+dt)=U(t)+\Delta t\cdot \delta \cdot L\cdot U(t) \end{array}$$ (5)

The advantage of our connectome frameworks is that neuronal connectivity is embedded in a standard stereotaxic coordinate system, so that a centroid can be estimated from each region and linear Euclidean distances can be estimated between centroids. Of course, real axonal connections run with curvatures as well as partially stronger unsmooth kinks. Therefore we speak here also only of estimations. We have presented in tutorial part 2 an approach to map Euclidean distances to graph edges by inserting nodes. Here, node *E* has been inserted to specify the distance between node *A* and node *B*. *D* is the diffusion constant and Θ controls the levarge and delay of the inserted node respectively:

$$\begin{array}{cc} \frac{dA}{dt} & =-D\cdot e_{1}\cdot A \end{array}$$ (6)

$$\begin{array}{cc} \frac{dE}{dt} & =D\cdot e_{1}\cdot A-\Theta \cdot D\cdot e_{1}\cdot E \end{array}$$ (7)

$$\begin{array}{cc} \frac{dB}{dt} & =\Theta \cdot D\cdot e_{1}\cdot E \end{array}$$ (8)

We validated the Gray-Scott implementation by applying it to a regular grid with the parameters published by Buric [89] and were able to generate concentration patterns (Fig P in S1 Text) typical of the Gray-Scott model. Furthermore, the Gierer-Meinhardt model was applied to a regular grid and tested with the parameters published in Koch and Meinhardt [56, 58]. As with the Gray-Scott model, the Turing pattern could be generated (Fig Q in S1 Text), which is also known from the literature.

In addition, we tested the runtime of the Gierer-Meinhardt model on a i7–6500U CPU (2,5 GHz). Here, a linear relationship between the number of iterations of the diffusion model and the computation time is found. 10^6^ iterations need about 60 s and 10^4^ iterations about 2 s (the multiple sclerosis mechanosensitive connectome was used). The number of meaningful iterations depends on the parameters of the model, the step size and the structure of the network and must be tested accordingly. Copying the concentration states at the time points for further analysis or graphical representation also takes time, but this was not considered in the values given above. In addition to the influence of the number of iterations, we studied the effect of the number of nodes. Here we found a quadratic relationship between the number of nodes and the computation time for the Gierer-Meinhardt model.

We are pleased to provide the Java source code on request and have also made it available for download at figshare (see below). A direct download is available for the connectivity data and for the executable Java version for Linux, Windows and iOs platforms on the neuroVIISAS web page.

## Results

### GM and MM in an embedded pathway of a subconnectome

**Fig 1:**
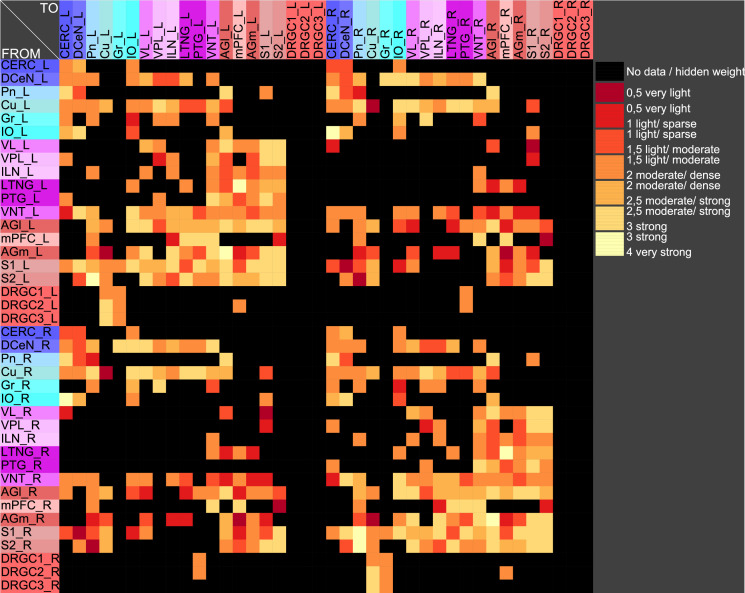
The weighted adjacency matrix of a bilateral mechanosensory subconnectome. The weighted adjacency matrix of the spinal cord, brainstem, diencephalic and cortical connectivity of the mechanosensory pathways. The last character of the area abbreviation indicates the side of the hemisphere: L: left hemisphere, R: right hemisphere. AGl: Lateral agranular prefrontal cortex, AGm: Medial agranular prefrontal cortex, CERC: Cerebellar cortex, Cu: Cuneate nucleus, DCeN: Cerebellar nuclei, DRGC1: Dorsal root ganglion of cervical segment 1, DRGC2: Dorsal root ganglion of cervical segment 2, DRGC3: Dorsal root ganglion of cervical segment 3, Gr: Gracile nucleus principal part, ILN: Intralaminar nuclei, IO: Inferior olive, LTNG: Lateral thalamic nuclear group, mPFC: Medial prefrontal cortex, Pn: Pontine nuclei, PTG: Posterior group, S1: Primary somatosensory cortex, S2: Secondary somatosensory cortex, VL: Ventrolateral thalamic nucleus, VNT: Ventral thalamus, VPL: Ventral posterolateral thalamic nucleus.

**Fig 2:**
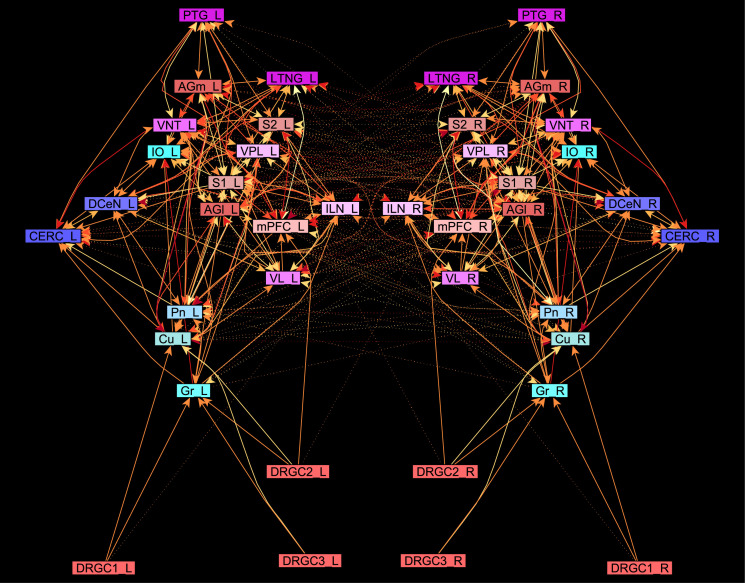
The weighted digraph of the bilateral mechanosensory subconnectome. The bilateral weighted digraph of the adjacency matrix shown in Fig 1. The dashed lines indicate contralateral projections.

The central pathways of mechanosensitivity originate from the central processes of pseudounipolar neurons in the dorsal root ganglia outside the spinal cord. The connections of the first cervical segments of this pathways were filtered from the complete connectome and both sides of the central nervous system were selected to compute the weighted, directed and bilateral adjacency matrix (Fig 1). These segments manifest particularly severe changes in multiple sclerosis [90, 91]. A symmetric graph representation of this matrix is shown in Fig 2. The source of the mechanosensitive pathway starts in the peripheral nervous system from the mechanoreceptors of the subepidermal layers of the skin or from internal organs. In multiple sclerosis, the mechanosensitive projection exhibits particularly pronounced alterations at specific sites in the spinal cord, primarily causing common neurological symptoms [92]. We consider the central projection from the dorsal root ganglion (first neuron) to the ipsilateral cuneate nucleus (second neuron) then to the right ventrolateral thalamic nucleus (third neuron) with termination in the right somatosensory cortex (terminal neuron). This mechanosensitive pathway is embedded in the bilateral network shown in Fig 2. The first three cervical spinal cord segments were used in the RD modeling.

#### Change of oscillations following reduction of connection weights

In the following, it was investigated how a single reduction of a connection weight affects the transmission in a coupled RD system. The weight reduction was realized by reducing normalized logarithmic transformed connection weights by 90% and keeping this reduction constant until the end of the process. To perform weighted network analysis, the ordinal weighted categories *x* were transformed to an exponential scale [93]:

$$\begin{array}{cc} f(x) & =10^{\left(-\frac{16}{49}\cdot {\left(x-4\right)}^{2}\right)} \end{array}$$ (9)

**Fig 3:**
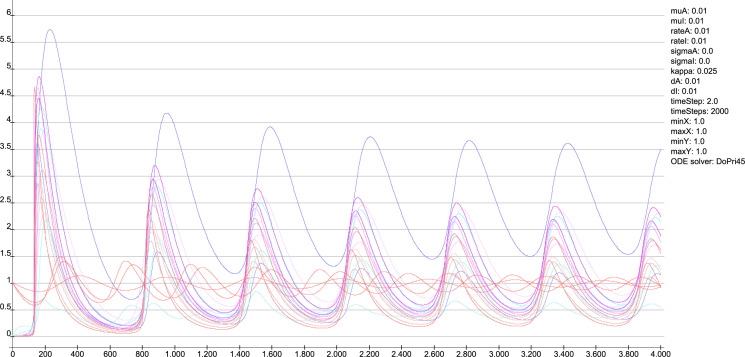
Functions of concentrations for regions of left side. The functions of concentrations of left hemispheric regions are shown for the GM RD-model. Initial conditions *V*_0_ = 1 and *W*_0_ = 1 were set for the dorsal root ganglia of the left side. The color coding of the functions in this diagram and all following function representations in diagram form are based on the color definition in the area hierarchy, which is simplified in the adjacency matrix in Fig 1. Thus, the colors of the columns and rows of the adjacency matrix represent the color scale of the functions. The x-axis shows the iteration steps of the function. On the y-axis the diffused concentrations are shown according to the applied models and functions. These axis assignments were also maintained uniformly for all subsequent function diagrams.

In particular the oscillation of the target region right somatosensory cortex was compared with the unchanged connections weights. The functions of concentrations of the GM RD model are displayed for all regions of the left side (Fig 3) and right side (Fig BJ in S1 Text) in distinct diagrams. Both presentations of concentrations are from the same GM RD-simulation. Right hemispheric (contralateral to initial conditions) regions show lower concentration amplitudes (first amplitudes are < 2.5) than regions on the ipsilateral side of initial condition for the DRGs.

**Fig 4:**
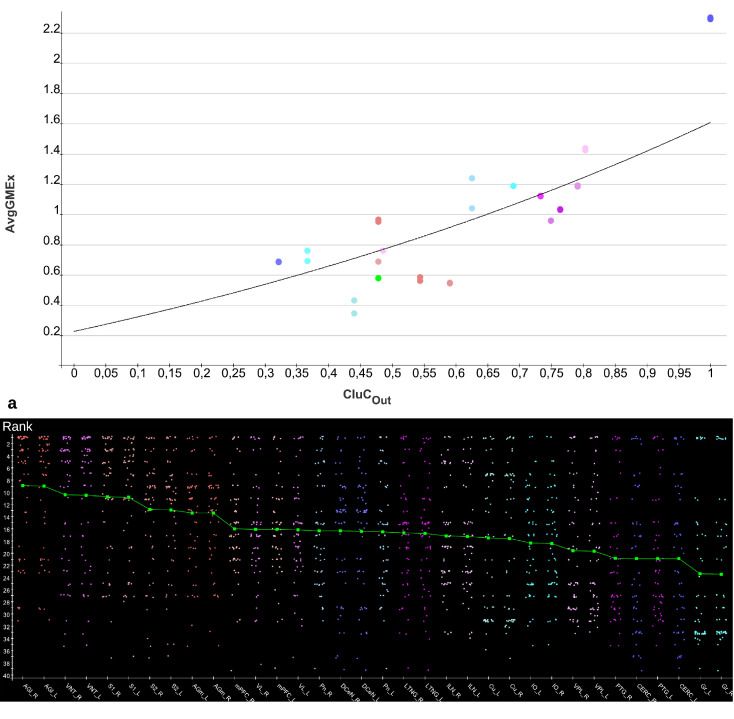
Relation of average concentrations and local network parameters. a) The correlation c = 0.726 of average concentrations (*AvgGEMEx*) and the *CluC*_*Out*_ ($\frac{DG_{in}}{DG_{a}ll}$) coefficient. b) Each point indicates the rank of a local network parameter of a region. The green curve represents the mean ranks. The colors correspond to the color assignments to the considered regions of the network, which were introduced in Fig 1 in the form of an adjacency matrix.

The functions of concentrations in Fig 3 and Fig BJ in S1 Text show variable amplitudes. The largest amplitude (blue) belongs to the cerebellar cortex which does not have direct efferents to non-cerebellar regions. The concentrations of the GM RD are relatively strong due to local connectivity in the cerebellum. Largest logarithmic correlations were found between the $\frac{DG_{in}}{DG_{a}ll}$ coefficient (convergent-divergent degree coefficient) (*c* = 0.726) (Fig 4 top) and the cluster-coefficient for output connections (*CluC*_*out*_) with *c* = 0.728. The ranks of local parameters show a relatively low average rank for the cerebral cortex region (Fig 4 bottom).

To apply the models to a connectome composed of regions affected in multiple sclerosis disease, the definition of the origins of the somatosensory pathway are particularly important, as this is where the initialization of the models as well as the change in pathway properties is set. The literature shows that the upper cervical spinal cord segments are particularly affected by demyelination [90, 91]. The central axons of the pseudounipolar neurons in the cervical spinal ganglia (1st neuron) project with a branch in the posterior funiculus to the 2nd neuron in the cuneate nucleus and a colateral via a switch in the Rexed layers V-VII (nucleus proprius of spinal cord) via the ventral spinothalamic tract to the ventral posterolateral nucleus of the thalamus. For simplicity, we consider only the projection of the pseudounipolar neurons via the posterior funiculus and, in particular, the cuneate funiculus to the cuneate nucleus. The regions of interest (ROI) are the DRG’s of the first three cervical segments of the left side, which were initialized with *V*_0_ = 1 and *W*_0_ = 1. The three concentration functions of the left DRGs start at 1 and were documented graphically (Fig BK in S1 Text). In this figure, as previously described, the individual regions were assigned the same colors as in other diagrams with function curves: The small blue curve indicates the concentration in the cuneate nucleus. The magenta curve indicates concentration in the contralateral ventrolateral thalamic nucleus. The brown concentration curve shows the concentration of activators in the contralateral (right hemispheric) somatosensory cortex. The weights of all three connections from left DRG’s 1–3 to the left cuneate nucleus were reduced by a factor of 0.3 for the whole simulation time. It turns out that amplitudes of the ipsilateral cuneate nucleus, contralateral ventrolateral thalamic nucleus and contralateral somatosensory cortex are larger than without weight reduction (Fig BL in S1 Text). Moreover, the oscillation pattern appears to be more regular, especially for the cuneate nucleus. Furthermore, the amplitudes of the contralateral somatosensory cortex increase obviously stronger than those of the cuneate nucleus or ventrolateral thalamic nucleus.

#### Stable coherent oscillations can be observed even for parameter variations

**Fig 5:**
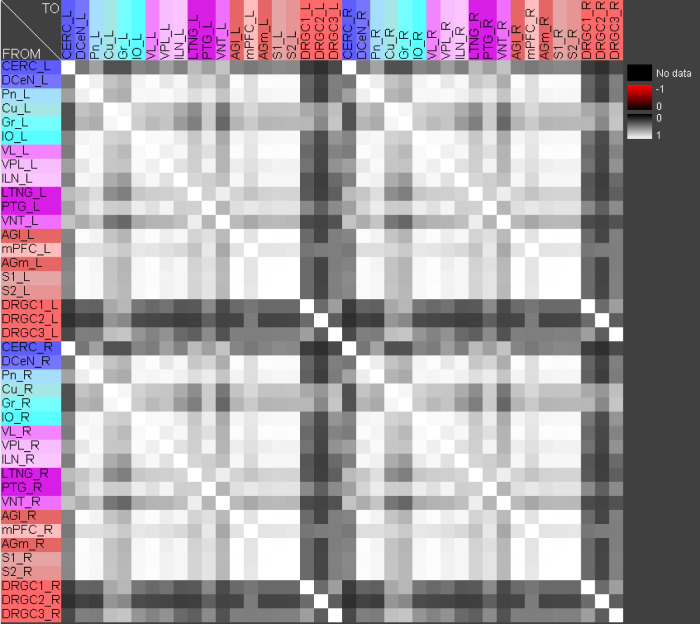
Average cross-correlation matrix of four parameters of the GM model. The average cross-correlation matrix of the variation of the four reaction constants is shown. This matrix has been used for further cluster analysis.

**Fig 6:**
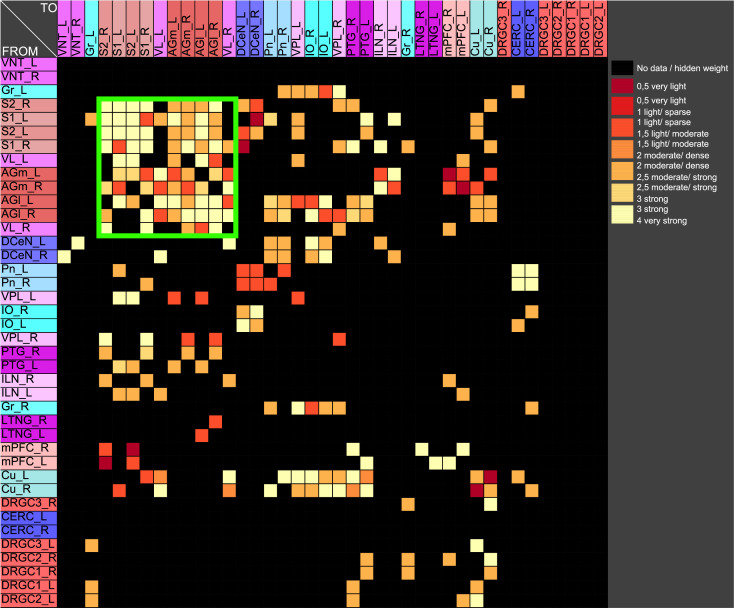
Average cross-correlation matrix of two reaction constants. The average cross-correlation of the variation of the two reaction constants has been analyzed by spectral clustering. A coherent group of regions (green rectangle) could be determined.

**Fig 7:**
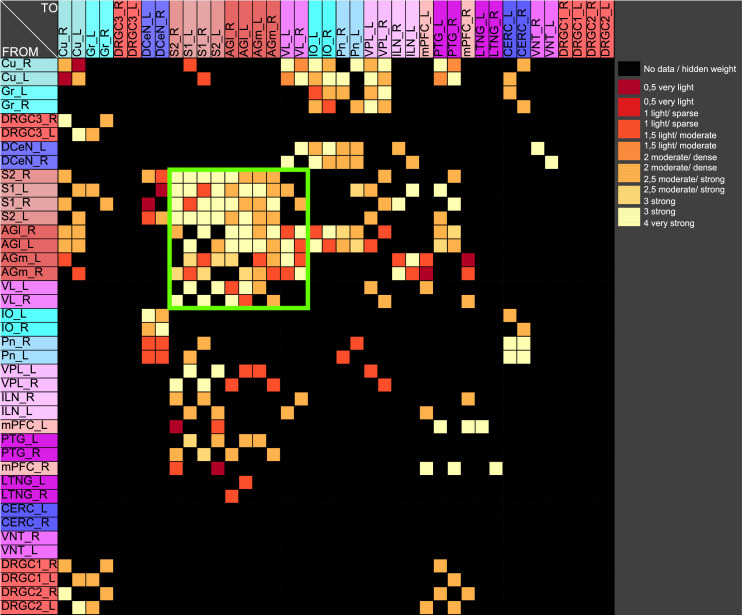
Average cross-correlation matrix of four parameters. The average cross-correlation of the variation of the two reaction constants and the two decay parameters for activators and inhibitors has been analyzed by spectral clustering. A similar group of regions (green rectangle) could be determined as found with a two parameter variation.

If pairs of regions have similar courses of concentrations over a range of different parameters of a RD-model then they might constitute coherent dynamic or functional groups. This has been investigated by varying RD-parameters of the GM and MM models. Here, the cross-correlations of all pairs of regions of the mechanosensitive subconnectome as introduced in Fig 1 has been determined for each variation of parameters. The average cross-correlations over all variations of parameters for each pair of regions is displayed as an average cross-correlation matrix. For the GM model the parameters reaction constants for the reaction substances A (*rateA*) and I (*rateI*) as well as the decay rates for the substance A (*muA*) and I (*muI*) were investigated over a range from 0.004 to 0.016 with a step size of 0.004. 256 simulations were performed (Fig 5). The average cross-correlation matrix for the variation of the two reaction constants is shown in Fig 6. The average cross-correlation matrix for the variation of the four parameters (2 reaction constants and the 2 decay parameters) is shown in Fig 7. Spectral clustering was performed to detect those regions which share similar average cross-correlations. The group of the 2 parameter reaction constant simulation with 16 simulations consists of left and right hemispheric primary and secondary somatosensory cortex, primary and secondary motor cortex and ventrolateral thalamic nuclei. Same regions build a cluster when the 4 parameters reaction constant and decay rates were varied.

**Fig 8:**
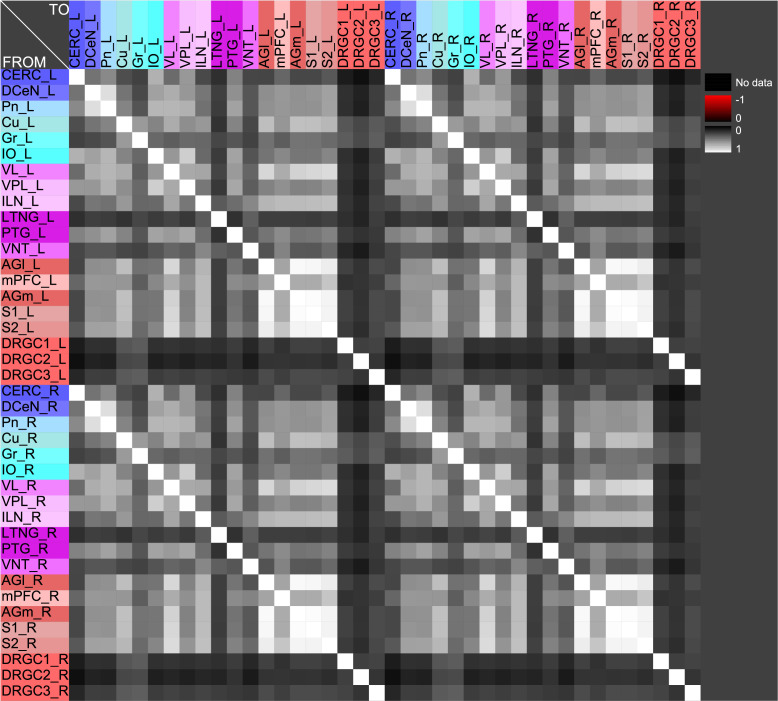
Average cross-correlation matrix of four parameters of the MM model. The average cross-correlation of the variation of four reaction parameters is shown. Large positive correlation values close to 1 indicate a strong similarity of concentrations of a pair of regions.

**Fig 9:**
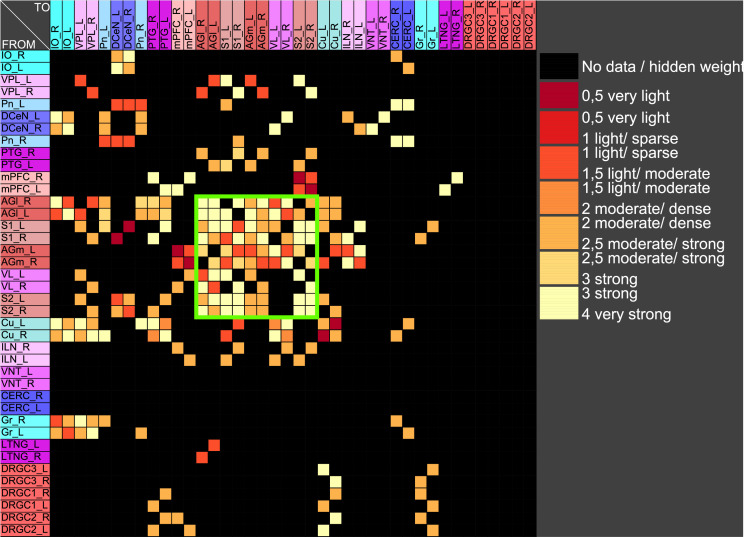
Spectral cluster analysis of the average cross-correlation matrix of the variation of four parameters of the MM model. The average cross-correlation of the variation of the four reaction parameters has been analyzed by spectral clustering. A similar group of regions (green rectangle) could be determined as found with the GM parameter variation.

The 4 reaction parameters *A*, *B*, *C*, *D* of the MM-model were varied (*A*: 10 − 15 with step-size: 1, *B*: 13 − 18 with step size: 1, *C*: 8 − 10 with step size: 1, *D*: 0.2 − 0.6 with step size: 0.1) over 540 simulations. The average cross-correlation matrix is shown in Fig 8. Spectral clustering of the average cross-correlation matrix determined the same cluster of coherently activated regions of the left and right hemisphere: S1, S2, AGl, AGm and VL (Fig 9).

**Fig 10:**
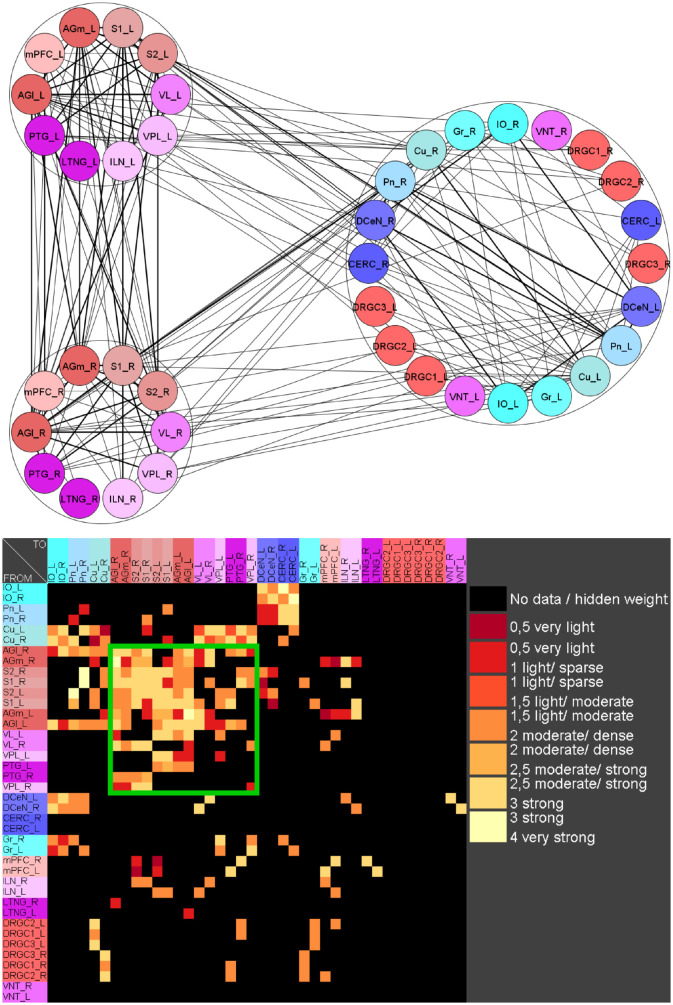
Modularity and spectral cluster analysis. The modularity analysis of connectivity similarity among regions generates 3 modules. The spectral cluster analysis of the connectivity matching matrix reveal a cluster with similar regions like those of the clustered cross-correlation matrices.

The modularity analysis of the adjacency matrix is shown in Fig 10. The regions are distributed over three modules. Cortical left and right hemispheric regions are separated in two different modules. These two modules contain again primary and secondary somatosensory cortex, primary and secondary motor cortex and ventrolateral thalamic nuclei. In addition the VPL, ILN, PTG and LTNG are assigned to these modules. The spectral cluster analysis of the connectivity matching matrix for input and output connections display same regions within a cluster that has been highlighted in Fig 10. Therefore, the clustering of the connectional structure of regions matches the dynamics of coherent regions with synchronous co-concentrations of the reaction-diffusion models. Furthermore, the coherency appears to be stable within the investigated space of parameters.

#### Cluster analyses generate groupings of regions with very similar dynamic properties even under the condition of parameter variations

To prove the effects of changes of parameters on the similarity of the oscillating functions of regions, the variation of parameters are analyzed by spectral [94, 95] and Markov clustering [96]. Spectral clustering techniques are using the spectrum (eigenvalues) of a similarity matrix and the Markov cluster algorithm (MCL) is an unsupervised clustering technique [96]. The following parameters were used (matrix values are normalized between 0 and 1):

- *Maximum value considered zero* (≥ 0) [Default: 1.0*e* − 7]: Test if matrix values are zero. Values below this threshold are set to zero (convergence can be faster)
- *Maximum difference between values* (≥ 0) [Default: 1.0*e* − 7]: If the difference of two values are below the threshold then they are considered as equal (termination of computing)
- *Loop gain* (≥ 0) [Default: 0]: Probability for considering a connection on the diagonal. If values > 0 then connectivity loops or self-connections are used. If 0.5 is set as a loop gain, then each value on the diagonal is set to 0.5
- *Inflation exponent* (Default: 2.0): Exponent is iteratively applied to matrix elements

In the first step the similarity matrix of either cross-correlations, co-activation or Kuramoto indices are calculated for a simulation. The similarity matrix allows the comparison of cross-correlations, co-activations or the Kuramoto indices between all pairs of regions. Either the cross-correlations, co-activation or Kuramoto index matrices are used to cluster the regions by spectral and Markov methods.

The parameters of the GM or MM models are modified, followed by the calculation of the similarities and the clustering of the regions. To compare the different clusterings the the Jaccard coefficient is used. For every cluster *X* in clustering *A* and every cluster *Y* of clustering *B* the Jaccard coefficient *J*(*X*, *Y*) = |*X* ∩ *Y*|/|*X* ∪ *Y*| is calculated. The corresponding cluster for *X* is the cluster *Y* that maximizes *J*(*X*, *Y*). If *Y* is the corresponding cluster for *X*, *X* does not have to be the corresponding cluster for *Y*. As a measurement of similarity of the two clusterings *A* and *B* we use the average Jaccard coefficient over all clusters of *A* and *B* and their corresponding cluster:

$$\begin{array}{c} \frac{\sum_{X\in A}{\text{max}}_{Y\in B}J\left(X,Y\right)+\sum_{Y\in B}{\text{max}}_{X\in A}J\left(Y,X\right)}{\left|A|+|B\right|} \end{array}$$ (10)

**Fig 11:**
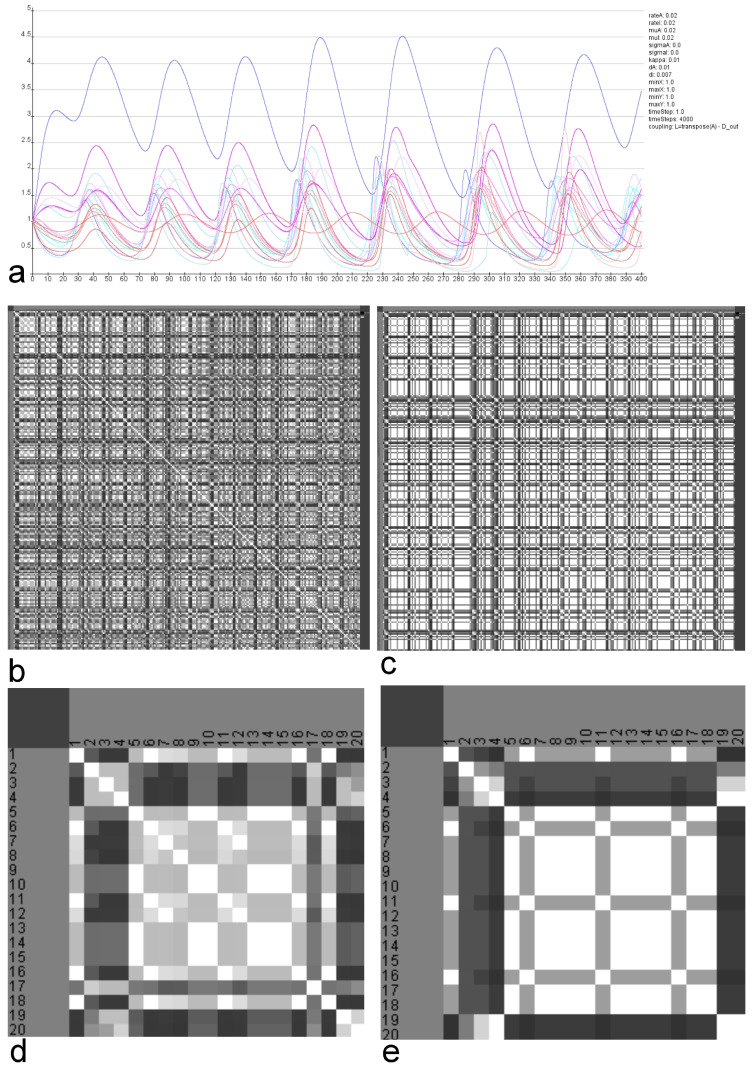
Similarities of clusterings referring GM parameter variation. The reaction parameters and decay rates for substances A and B were varied 256 times. a) RD functions of all regions using default parameters. b) Similarities of the clusterings using spectral clustering (Light: high similarity, dark low similarity). c) Similarities of the clusterings using Markov clustering (Light: high similarity, dark low similarity). d) Magnification of upper left corner of the matrix in b). e) Magnification of upper left corner of the matrix in c).

**Fig 12:**
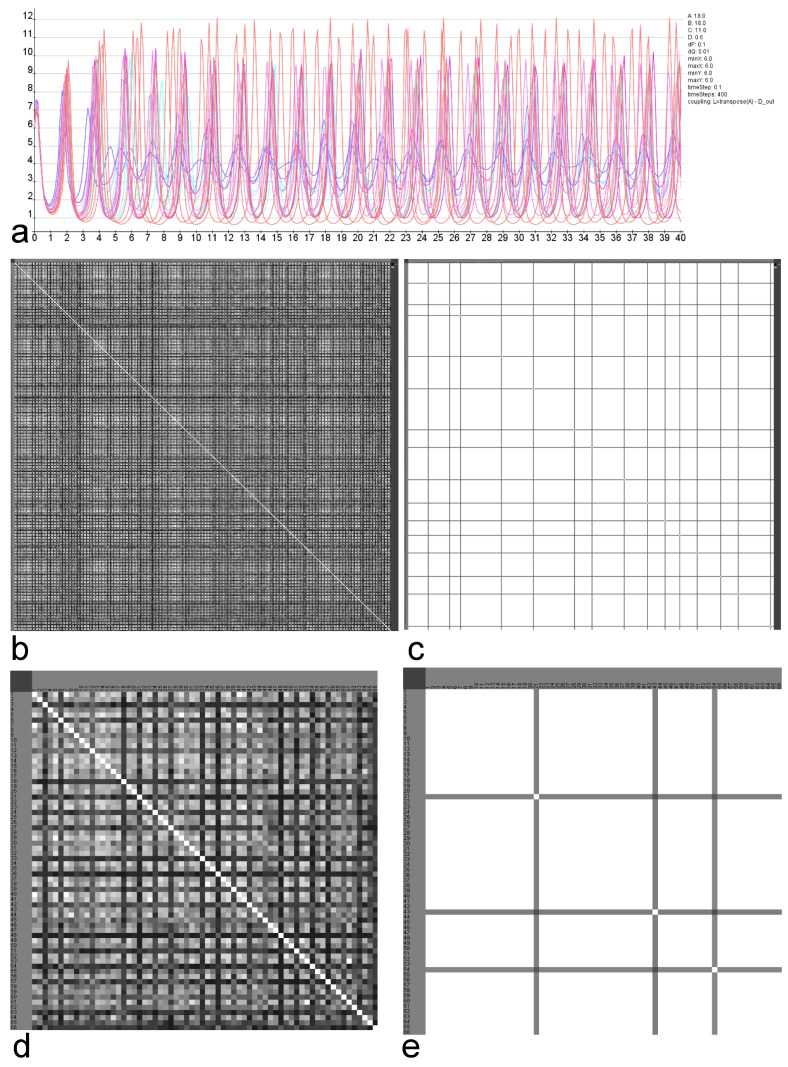
Similarities of clusterings referring MM parameter variation. The reaction parameters A-D were varied 375 times. a) RD functions of all regions using default parameters. b) Similarities of the clusterings using spectral clustering (Light: high similarity, dark low similarity). c) Similarities of the clusterings using Markov clustering (Light: high similarity, dark low similarity). d) Magnification of upper left corner of the matrix in b). e) Magnification of upper left corner of the matrix in c).

Following parameter variations the similarities of the calculated clusters are visualized in a matrix (Figs 11 and 12).

In the case of the GM model the variation of reaction parameters rateA, rateI, muA and muI range from 0.004 to 0.016 with a step size of 0.004 leads to 256 combinations of these parameters. For each of the 256 combinations the similarity of functions between pairs of regions has been calculated by the cross-correlation. Referring to this similarity the regions were clustered and the comparison of these 256 clusterings is visualized in a matrix. This matrix shows the pairwise similarity of region clusterings that result from the simulation using a particular selection of reaction parameters.

The RD functions of all regions using default parameters are shown in Fig 11a. The similarities of the clusters using the spectral clustering is shown in Fig 11b. The magnification (Fig 11d) shows a pattern in this matrix. For example the group of parameter sets 5–16 lead to very similar region clusterings. The similarities of the Markov clusterings based on the same parameter variation is shown in Fig 11c. Again the magnification displays homogeneous groups of parameter sets indicating a larger stability of RD functions.

In a comparable way, the similarity, respectively, dissimilarity of clusters of cross-correlations of 375 sets of parameters of the MM model were analyzed. The reaction parameters *A*, *B*, and *C* range from 14 to 18 with a step size of 1. Reaction parameter *D* ranges from 0.2 to 0.6 with a step size of 0.2 (*dP* = 0.1 and *dQ* = 0.01). The RD functions of all regions using default parameters are shown in Fig 12a. The similarities of the clusters using the spectral clustering is shown in Fig 12b. The magnification (Fig 12d) also shows a pattern. For example the group of parameter sets 5–17 lead to very similar clusterings. The similarities of the Markov clusterings based on the same parameter variation is visualized in Fig 12c. The magnification displays highly homogeneous groups of parameter sets indicating a very large stability of RD functions.

#### Functionally similar regions can be reconstructed based on their dynamic properties in both the GM and MM models

By comparing the coherent dynamics between MM and GM processes, we want to find out to what extent these two models produce similar results in the same connectome. If similar dynamical behavior of functionally similar regions is obtained in different models, this indicates the reproducibility of a result by another model and thus a certain stability of the dynamics independent of a specific model. Furthermore, we can consider to what extent the similar results of both models can be explained by the specific connectivity rather than by minor changes in parameter settings of the models. We expect that functionally intensive or densely interconnected areas of somatosensory and somatomotor cortical areas will exhibit similar diffusion dynamics and therefore form greater synchronization of concentrations outputs of diffusion functions. This can be tested by comparing pairs of regions with large coherence using a cross-correlation analysis.

**Fig 13:**
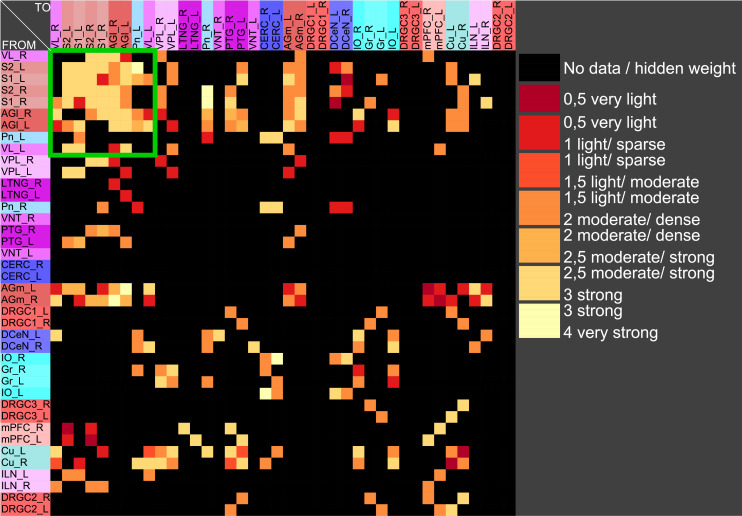
Result of clustering the cross-correlation matrix of a GM process. The somatosensory regions constitute a cluster (green rectangle).

**Fig 14:**
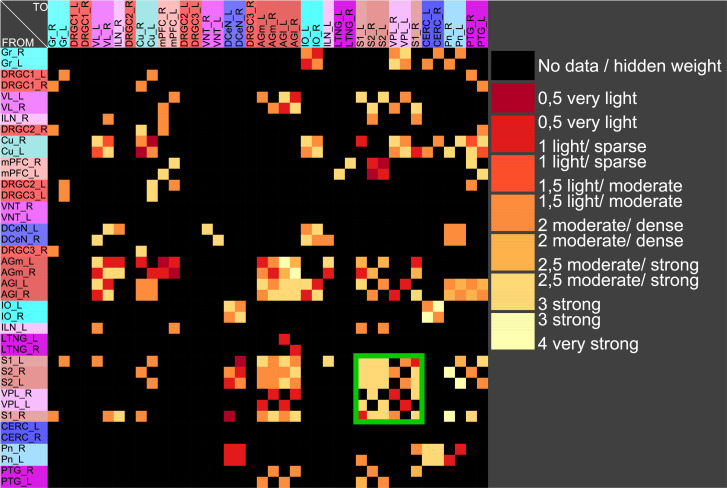
Result of clustering the cross-correlation matrix of a MM process. The somatosensory regions are contained in the same cluster like in the GM model.

The somatosensory regions constitute a set of nodes with common dynamical behavior. A GM and a MM process was applied to the mechanosensory subconnectome. The cross-correlation matrix of co-activations has been determined and analyzed by spectral clustering to obtain groups of regions that share similar synchronous behavior. The left and right hemispheric primary and secondary somatosensory regions build such a group of regions in both RD models (Figs 13 and 14). Moreover, the MM model allows a separation of primary and secondary motoric regions as well as primary and secondary somatosensory regions (Fig 14).

#### The bifurcation analysis suggests a stable oscillatory behavior of the GM model

A comparable approach has been suggested elsewhere in order to predict the morphology of pattern generation in a regular lattice [97]. In order to find the oscillation state and the fix-point state of the GM model a simplification using default parameters has been performed. We consider a single PDE without coupling and *σ*.

$$\begin{array}{c} \frac{\partial u}{\partial t}=0.01\;\frac{u^{2}}{v}\;-\;b\;u\;+\;a \end{array}$$ (11)

$$\begin{array}{c} \frac{\partial v}{\partial t}=0.01\;u^{2}\;-\;0.01\;v \end{array}$$ (12)

**Fig 15:**
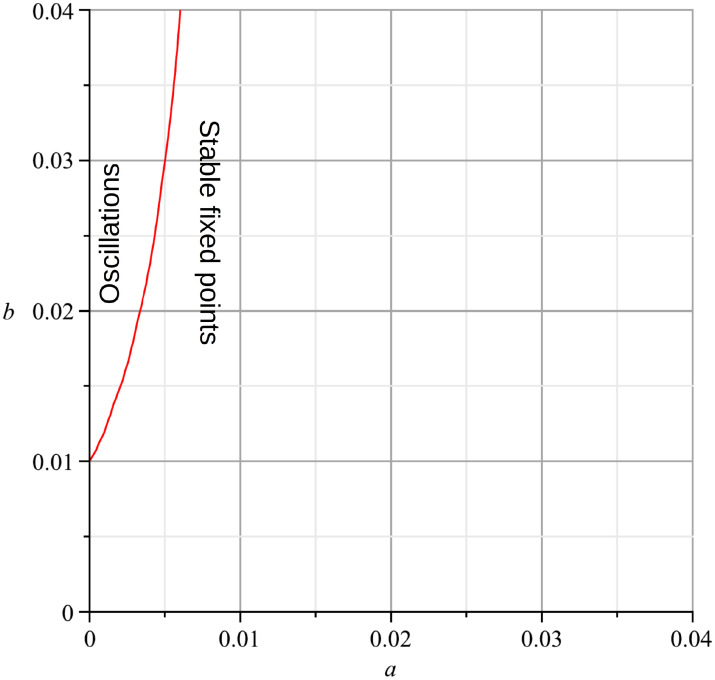
Linear stability analysis of the GM model. The curve shows where the real part of the first eigenvalue of the Jacobian matrix in the fix-point is zero in dependence of the parameters *a* (sigmaa) and *b* (mua).

**Fig 16:**
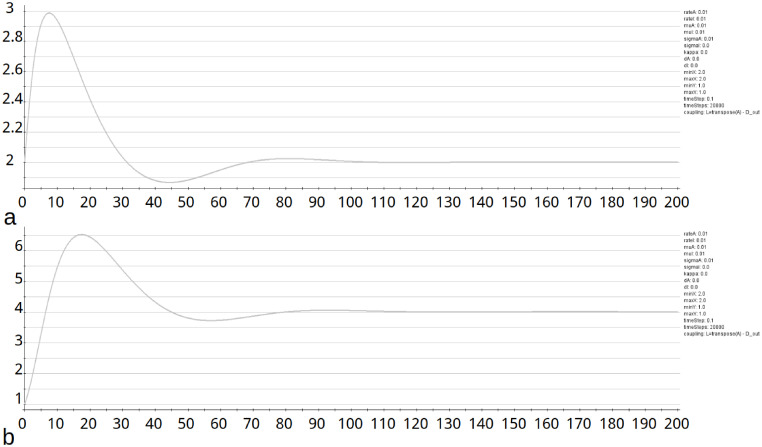
Stability of function *u* and *v*. a) The GM model was applied to exactly one non-connected node to show that the function *u* has a stable progression above a certain number of iterations. The function of *u* for *a* = *b* = 0.01 shows stability at about 100 iterations. b) For the same parameters as given before, the function v is given.

**Fig 17:**
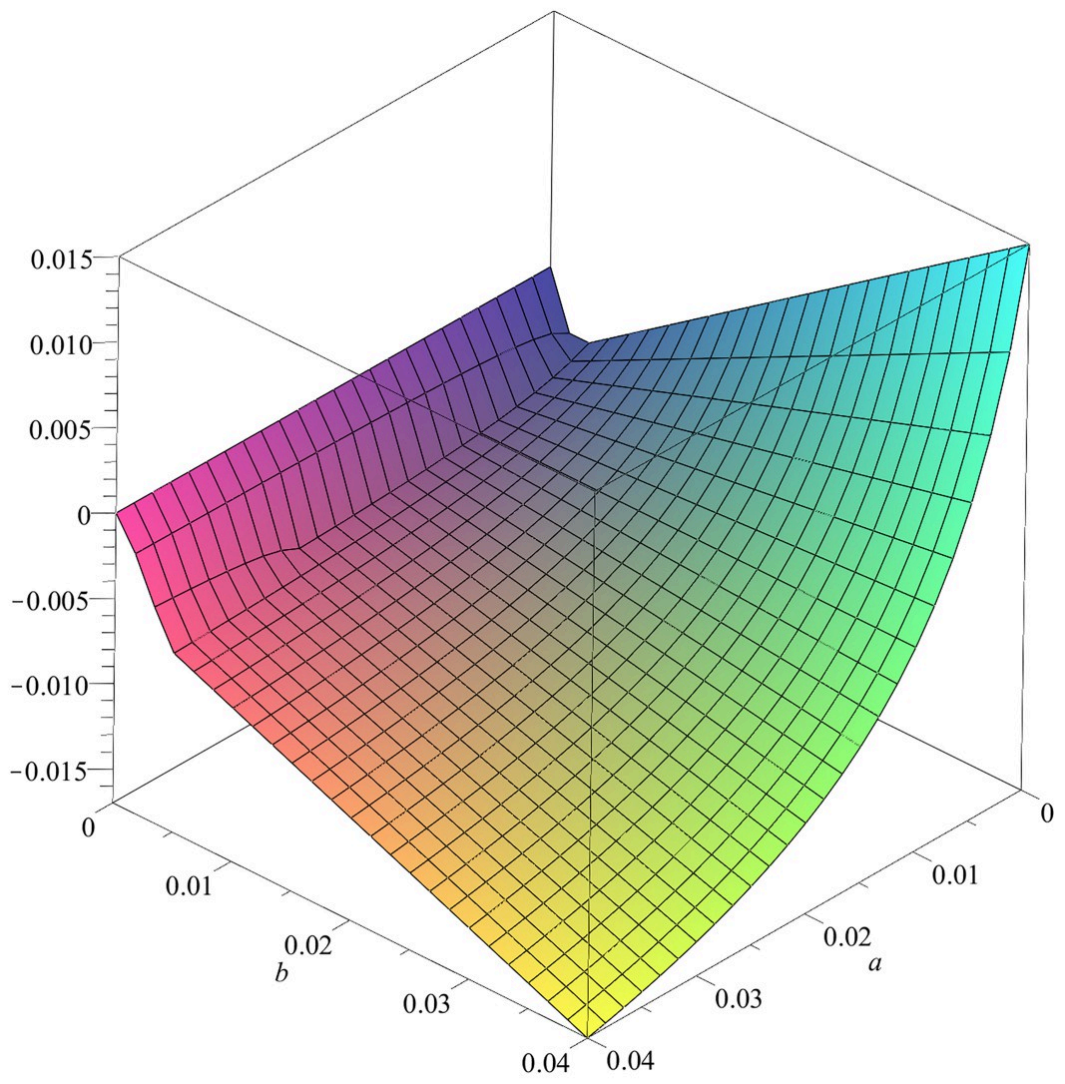
Real part of first eigenvalue of the GM model. The real parts of the first eigenvalue of the Jacobian matrix in dependence of the parameters *a* (sigmaa) and *b* (mua).

**Fig 18:**
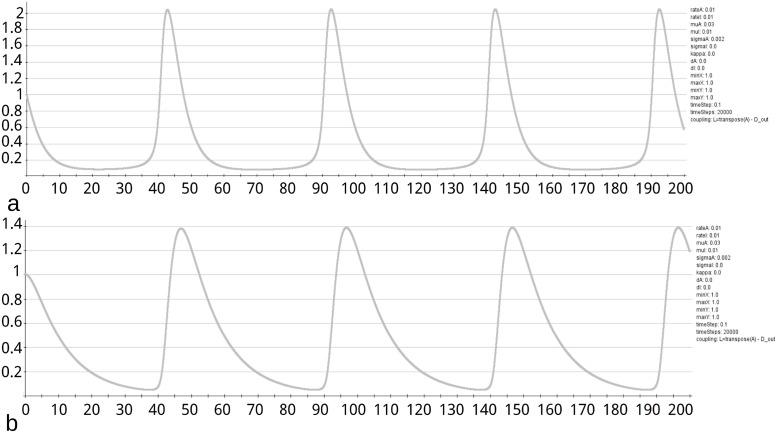
Oscillations of functions *u* and *v*. a) In addition to the stability of the function *u* in Fig 16 the regularity of the oscillations shall be shown here. The regularity extends over all performed iterations. b) The regularity of the oscillations of the function *v* is shown here. As with the function *u*, the regularity extends over all iterations performed.

Analyzing the fixed points of the GM model by examing the eigenvalues of the Jacobian, reveals the (*a*, *b*) plane is divided into an oscillatory region and a region with stable fixed points (Fig 15). For pairs of parameters (*a*, *b*) on the right side of the curve *r* is negative, what indicates stability in the fix-point. E.g., the fix-point *u* = 2 and *v* = 4 for the parameters *a* = *b* = 0.01 and is stable. This is demonstrated for a single, non connected node in *neuroVIISAS* (Fig 16a and 16b). The real part *r* of the first eigenvalue as a function of parameters *a* and *b* is displayed in Fig 17. For parameters (*a*, *b*) on the left side of the curve the GM model has oscillatory behavior (Fig 18a and 18b). The same parameters were applied to a network with coupling of PDEs. It is demonstrated that the oscillatory behavior is still stable. This becomes apparent from the figures of the functions *u* and *v* (Fig BM and Fig BN in S1 Text).

#### The Wilson Cowan model, like the GM and MM models, leads to stable oscillations with coherent patterns

The neural mass model of Wilson and Cowan (WC) [98–100] was used (“WC-simulation” in *neuroVIISAS* [101]) to obtain an visual impression of the dynamics (Fig BO in S1 Text) which results from the same network as used for the GM and MM models (Figs 1 and 2). We used the parameters for a limit cycle oscillation of a single Wilson-Cowan oscillator: *a*_*E*_: 1.2, *a*_*I*_: 2.0, *c*_*EE*_: 5.0, *c*_*II*_: 1.0, *c*_*IE*_: 6.0, *c*_*EI*_: 10.0, *θE*: 2.0, *θI*: 3.5, *η*: 20.0, *P*: 0.25, *E*_0_: 0.1, *I*_0_: 0.1, time steps: 1000, time step: 0.1. After about 100 steps the WC model gives rise to stable oscillation within the interconnected regions. The similarity of functions is largest within the peaks of oscillations. The Kuramoto index over all regions displays a regular and stable course. The DRG regions without input connections show a small initialization peak and after about 100 steps they show, as expected, a flat line. Principally, the WC model generates strongly regular oscillations which exhibit some differences when comparing with GM and MM models. The MM model produces much more irregular functions of concentrations with an obvious lower synchronized behavior as can be seen in the smaller Kuramoto indices. The GM model generates more regular oscillations with damping of amplitudes.

#### The GM and MM models produce different dynamics in degree preserving surrogate networks

The same number of nodes and connections were used by generating Erdös-Rényi (uniform distributed edges) [102], Watts-Strogatz (small-world) [103], Barabasi-Albert (scale-free) [104], Ozik-Hunt-Ott (small-world) [105], rewiring [106], Klemm-Eguiluz (growing scale-free) [107] and multifractal (cluster coefficient) [108] randomized networks to investigate the effects of structural changes of a network to GM and MM models. The GM model is able to produce regular and stable oscillatory functions within nearly all random networks with the exception of the rewiring network (Fig BH in S1 Text). The cross-correlation matrices display large interregional correlations of function similarities. In the case of the rewiring network the Kuramoto index shows strong changes and functions in early stages of the iterations are damped. Interestingly, the Watts-Strogatz model generates a relatively homogeneous network with regard to edge distribution. However, a chessboard like pattern of large and small cross-correlations can be seen in the cross-correlation matrix. The MM model generates much more irregular oscillations in the different random networks. An obvious feature is the slight overlapping low pass oscillation which is missing in the rewiring network. The rewiring network appears to generate more regularity of the oscillating functions. The limit cycles int phase diagram are lying closer together indicating more similarity of waves (Fig BI in S1 Text).

#### Dynamic weight changes of the GM model lead to a change in the oscillation of the network nodes of the mechanosensitive pathway

**Fig 19:**
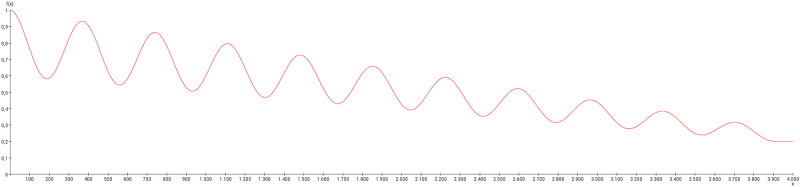
Weight modulation function. This weight modulation function (damped cosine function) is applied to the GM RD-system. Initial conditions *V*_0_ = 1 and *W*_0_ = 1 were set for the dorsal root ganglia of the left side.

In the following experiment, the weight modulation has been adapted to a progressive relapsing multiple sclerosis disease progression (Fig 19).

By comparing the effect of nonlinear weight modulation of the three connections from the left DRG 1–3 to the left cuneate nucleus with the connectome under normal conditions, the concentrations of the GM RD-system show some remarkable differences. The initial amplitudes of concentrations in the ventrolateral thalamic nucleus and somatosensory cortex are similar (Fig BP in S1 Text) when compared with those in Fig BK in S1 Text. The following amplitudes are slightly more irregular than those in Fig BK in S1 Text with unchanged weights. Furthermore, some small phase shifts are visible when comparing the concentration functions of the contralateral ventrolateral nucleus and somatosensory cortex of nonlinear weight modulated simulation in Fig BP in S1 Text with those in Fig BK in S1 Text. When analyzing the average concentrations by differential connectome analysis [53], strongest differences of the control connectome and weight-reduced connectome are localized at the cuneate nucleus, posterior group of the thalamus, gracile nucleus, lateral thalamic nucleus, intralaminar thalamic nuclei and ventral posterolateral thalamic nucleus. Thalamic nuclei appear to be most affected by a reduction of weights of primary neurons along the mechanosensitive pathway.

To study more specifically the effect of weight reduction within the regions of a pathway embedded in the network, we determined the input regions with diffusion outputs to the regions of the mechanosensitive pathway (Fig BD in S1 Text—Fig BG in S1 Text). It turns out that the ventrolateral thalamus receives slightly changed input following a weight reduction of the afferents of the cuneate nucleus. It is conceivable that such an effect could also occur in multiple sclerosis and lead to transmission disturbances of sensory or mechanosensitive signal patterns with resulting neurological symptoms. These changes are visible after sorting the input nodes by the average concentrations of activators (Fig BG in S1 Text). The ranking of the right cuneate nucleus and the right secondary somatosensory cortex changed (Fig BF in S1 Text in comparison with Fig BG in S1 Text). Stronger changes of ranks of regions with regard to the average concentrations are found for afferents of the right primary somatosensory cortex (Fig BF in S1 Text in comparison with Fig BG in S1 Text).

**Fig 20:**
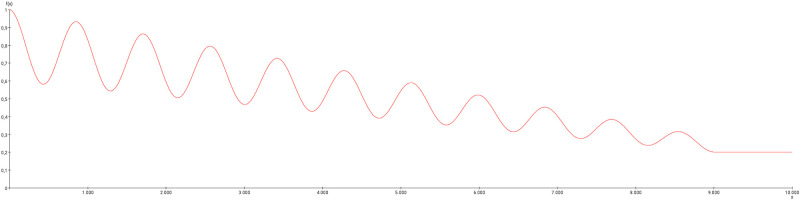
Weight modulation function. The weight modulation function (damped cosine function) is applied to the GM RD-system. Initial conditions *V*_0_ = 1 and *W*_0_ = 1 were set for the dorsal root ganglia of the left side. The number of iterations is 10000. The amplitudes were fixed to 10 and the lower value of the weight reduction was set to 0.1.

Long term changes of the oscillation pattern of the regions of the mechanosensitive pathway were observed when iterations were extended up to 10000 (2 [*timeStep*] × 5000 [*timeSteps*] = 10000) (Fig BQ in S1 Text). The Kuramoto order parameter *r* [109, 110] was calculated to estimate the extent of synchronization or desynchronization of the oscillations of concentrations of the regions of the mechanosensitive pathway. The weight modulation function (Fig 20) has been applied to the output connections of the first three cervical DRGs to the cuneate nucleus of the left side. The progression of concentrations of the GM RD-model for the ventrolateral thalamic nucleus and the primary somatosensory cortex of the right side and the cuneate nucleus of the left side is shown in Fig BQ in S1 Text. By applying initial values *V*_0_ = 1 and *W*_0_ = 1 to the left DRG 1–3 regions the oscillations of the regions of the mechanosensitive pathway develop a slow decrease of amplitudes without change of frequencies or phases. Beside cuneate nucleus, ventrolateral thalamus and primary somatosensory cortex the DRG 2 is plotted in Fig BQ in S1 Text, too. DRG 2 shown an obvious decrease of amplitude size due to its lack of inputs. Following the application of the weight modulation function shown in Fig 20 the amplitudes of concentrations of all these regions of the mechanosensitive pathway decrease until iteration 4700 and then they increase. The Kuramoto order parameter indicates a slight decrease of synchronization of the oscillations of concentrations of the three mechanosensitive regions (Fig BR in S1 Text).

#### Dynamic weight changes of the MM model lead to a change in the oscillation of the network nodes of the mechanosensitive pathway

The MM RD system works on another time scale than the GM RD. Here, 400 time steps with a step size of 0.1 allows a sufficient survey of the progression of the functions of the concentration of ROIs. To allow comparison of GM and MM the same DRGs of the cervical segments 1–3 of the left side were related to constant non-zero initial conditions with *P*_0_ = 6 and *Q*_0_ = 12 (Fig BS in S1 Text). Initial amplitudes of the ipsilateral cuneate nucleus, contralateral ventrolateral thalamic nucleus and somatosensory region are slightly delayed or shifted to the right on the x-axis in Fig BT in S1 Text. The amplitudes of concentrations of the cuneate nucleus (blue curve) are better visible in Fig BT in S1 Text because the overlap is not as strong as in Fig BS in S1 Text. The amplitudes of the concentration in the weight-reduced (step function) model of the contralateral somatosensory cortex appear to be smaller than in the control connectome without weight reduction.

**Fig 21:**
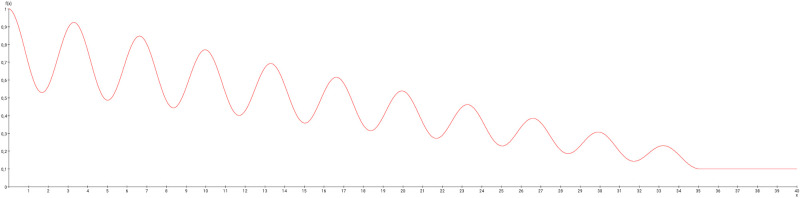
The weight modulation function. This weight modulation function (damped cosine function) is applied to the MM RD-system. It has the same scale of time like the MM RD simulation.

A comparable (Fig 19) damped cosine function as a weight modulation function (Fig 21) was applied to the MM RD simulation with the same initial conditions like those 19 in S1 Text. The concentration curves appear to have a lower coherency than in the case of a constant weight reduction (Fig BU in S1 Text). This can be seen particularly well for the function course of the cuneate nucleus, which was assigned the color cyan. Before the reduction of the edge weights, the amplitudes of the cuneate nucleus are hidden by a grouping of amplitudes of most other regions in smaller areas or smaller sections on the x-axis. In other words, most of the amplitudes are co-located and co-occur. If the weights are now reduced, the amplitudes of the cyan coded cuneate nucleus move out of the amplitude group, so that the coherence or synchronization of the amplitudes has slightly decreased.

#### The introduction of distance information creates a damping of the oscillations in the GM model

**Fig 22:**
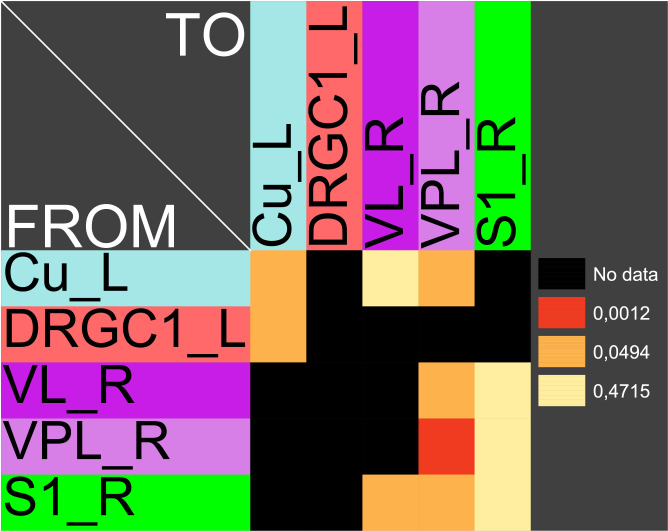
Adjacency matrix. Adjacency matrix of mechanosensitive subnetwork.

**Fig 23:**
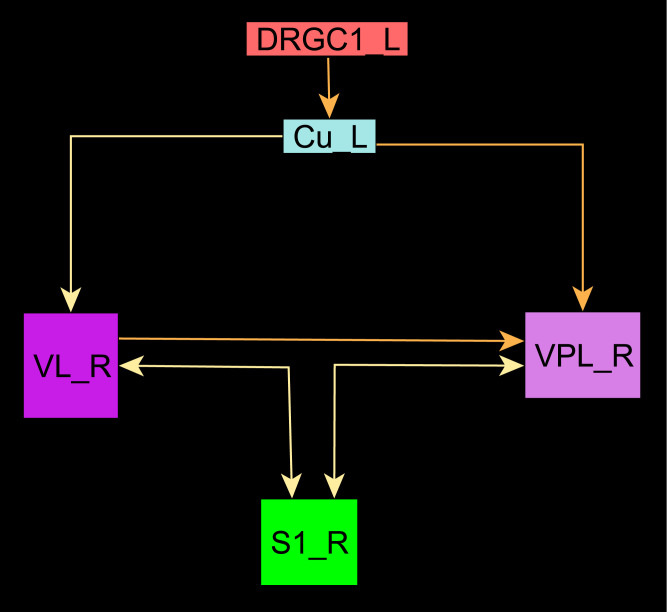
Network representation of the adjacency matrix. The network of the adjacency matrix of Fig 22 is visualized.

The Euclidean distance of the major mechanosensitive pathway from the first dorsal root ganglion and cervical spinal cord segment through the ipsilateral cuneate nucleus, contralateral ventrolateral thalamic nucleus and ventroposterolateral thalamic nucleus to the primary somatosensory cortex (Figs 22 and 23) were used in a GM RD model. When ignoring the distance parameter the oscillation of concentrations is shown in Fig BV in S1 Text. By applying Euclidean distance (d) and log-transformed weights through the following transform *F*_1_ × *d* + *F*_2_/*w* + *S* (*F*_1_ = 0.01, *F*_2_ = 0.01, $w=10^{\left(-\frac{16}{49}\cdot {\left(x-4\right)}^{2}\right)}$, *S* = 1) to the GM RD process a damping of oscillations was found (Fig BW in S1 Text).

## Discussion

Three different RD models were applied to directed and weighted networks and connectomes. It turns out that the GS model does not generate prolonged oscillations which are necessary to investigate the propagation of dynamic signals or the spreading of information through a connectome. The GM and MM RD models are able to generate oscillation patterns in directed and weighted networks. The implemented GM model generates in a regular lattice Turing-like patterns (white eye Turing pattern) [111–113]. The oscillations of the concentrations in the studied networks and circuits are basically similar to the oscillations of RD systems documented in the literature [56, 114]. However, it cannot be said with absolute certainty that there is a perfect match between the oscillation behavior of the procedures we implemented and the same procedures documented in the literature. One reason for this is that different solvers can be used to solve the differential equations and initial conditions can vary. The GM and MM models are stable with regard to additive noise and stochastic noise of the Ornstein-Uhlenbeck process (Fig Y in S1 Text). In addition to these candidate models, there are other promising diffusion models (Barkley, Brusselator, Keller-Segel) that can be applied to networks in the sense of network diffusion [115–120]. However, in the context of this work, we focused on RD models that seemed promising in terms of accounting for weights, distances, and diffusion directions. Therefore, the above-mentioned models were applied to the mechanosensitive pathway embedded in a partial connectome consisting of bilateral regions of the brainstem, diencephalon and cerebral cortex. This subnetwork is of particular interest due to the multiple sclerosis demyelination disease. The mechanosensitive pathway is of special interest because most patients are suffering because of the main symptoms of pain, hypoesthesia and paresthesia [121, 122]. Other complexes of main symptoms (urinary, bowel, musculoskeletal, throat, speech, vision, central symptoms) and their associated pathways are not considered here. A control connectome without changes was compared with a connectome reflecting the demyelination disease progression of the dorsal fascicle of the spinal cord which contains mechanosensitive fibers from dorsal root ganglia to the cuneate nucleus for cervical segments.

Using linear stability analysis [123–131] and bifurcation analysis [131–135] we computed the oscillation state and fix-point state of the GM model. Now it is possible to generate directly oscillatory states of the GM model and study the its dynamic behavior in connectomes. The effect of parameter variations such as rateA, rateI, muA and muI on the coherence of functions between all pairs of regions provides information of the stability of the RD models. We observed similar clustering of coherent dynamics of regions when changing parameters. This stability was found for the GM as well for the MM model. When comparing the Wilson-Cowan neural mass model with the MM and GM models it turned out that the Wilson-Cowan model produces similar oscillations like the GM model. If the WC, GM and MM models are applied to the same network, they produce distinct spectra of amplitudes and frequencies. Furthermore, we detected oscillatory stability if the connectivity changes from the empirical biological connectome to a scale-free or small-world null model. Interestingly, the oscillatory behavior changes strongly when using a rewiring null model.

By varying reaction parameters of the GM and MM models it was found that the same regions give rise to dynamically related groups. The primary and secondary somatosensory and motoric cortical regions as well as the ventrolateral thalamic nucleus of both hemispheres were assigned by spectral clustering to the same cluster. This group constitutes a functional cluster for controlling somatomotor (AGl, AGm) behavior in dependence of sensory input (S1, S2) from the ventrolateral thalamic nucleus.

In the case of a weight reduction by a step function obvious changes of oscillations in the target region of the mechanosensory pathway which is the contralateral primary somatosensory cortex were found. Moreover, it is possible to change the connections weights in analogy to a progressive remitted disease progression of the disease (it is one of the four forms of multiple sclerosis summarized as follows: Clinically isolated syndrome (CIS), Relapsing-remitting MS (RRMS), Primary progressive MS (PPMS), Secondary progressive MS (SPMS)) with distinct changes of oscillations in the contralateral somatosensory cortex [66, 67, 136]. In the meantime, there are models to simulate the clinical course of the aforementioned CIS relatively accurately [71, 137, 138]. In the present study, however, we have restricted ourselves to simply describable functions of weight modulation in order not to consider too many different influencing factors. After applying such a periodic weight reduction (Fig AH in S1 Text), increased amplitudes of the contralateral target region (somatosensory cortex) and slight phase shifts were found. The effect can be carefully interpreted as a dynamic change caused by a structural change in the organization of the network’s connection weights. Moreover, the change of dynamics or oscillation of regions within the mechanosensory pathway may shape the demyelinations effects resulting in paresthesias in the contralateral somatosensory cortex. By investigating local effects of activator concentration of afferents of the mechanosensitive pathway, a change of ranks of regions projecting to the ventrolateral thalamic nucleus and the somatosensory cortex was found. This could indicate a change of input priority of afferent regions or a disruption of temporal signal patterns processed by these thalamic and somatosensory regions. Further, models of weight modulation should be considered as the one suggested by [139]. So far, there exist no computational models that shape the effects of demyelination at the level of networks and connectomes. Most modeling research of multiple sclerosis is performed at the cellular level [71, 137, 138, 140–144], statistically [145] or in MRI studies [146, 147]. We have mentioned above the comparison and application of changes in connection weights with the courses of neurological deficits of CIS of multiple sclerosis. A disadvantage of using RD models is the comparability of time scales to the clinical courses of multiple sclerosis. The clinical courses of multiple sclerosis are over years to decades with oscillatory phases that can last for several months. The RD models are represented by the iterations and iteration step sizes of the functions. The relationship between these fundamentally different scales of neurological progression and function iteration of RD models remains to be worked out.

Further options for extending more parameters to RD models are the consideration of delay differential equations [148–150] to better take note of Euclidean distances in combination with weights of a connectome. A number of works have already investigated the introduction of delays in reaction diffusion systems [133–135, 151], which is also applicable to the future extension of the RD models studied here. Moreover, the consideration of region volume estimates could introduce an important parameter for preferred pathways of the transmission of concentrations of RD through a connectome with many alternative pathways [86, 152].

The implementation of GM and MM RD in the *neuroVIISAS* framework offers the possibility to either use directional and/or weight information of connectivity. Thus, a RD modeling tool has been made available which enables investigators to apply it to non-directed binary adjacency matrices, complex weighted and directed connectivity data. A further development of these RD approaches studied here is to consider distance-dependent delays in diffusion solved by using delay differential equations which allow the control of a time parameter within a simulation.

Those sets of parameters in the GM and MM models which generate oscillations in empirical weighted and directed networks like the mechanosensitive pathway network give rise to oscillations in other empirical networks with similar global network features. However, it needs to be analyzed how the amount of reciprocal connections, modularity and homogeneity (variability of degrees) may influence RD parameters with regard to the stability of oscillations.

By investigating specific pathways of an empirical network which undergo neuropathological changes like pain pathways, mechanosensory pathways and the visual pathway new techniques are required to judge specific contributions of connections in such pathway-subgraphs. This could support the comprehension how embedded pathways in networks may transmit signals through multiple reciprocal connections as well as alternative routes through connectomes [86]. How signals are transmitted in networks has traditionally been analyzed in gene and protein networks by *network propagation* and *network spreading* analysis [153–157]. *Signal propagation*, *signal transmission* and *signal routing* analysis is used to investigate the traveling of signals in generalized and neuronal networks. It has been shown that propagation patterns can be measured in humans and that these patterns are remarkably stable [158]. Therefore, it appears promising to further investigate signal propagation through pathways in partial connectomes shaping certain functions which are disrupted in multiple sclerosis. An important area of work arises from the structured analysis of stimulus response patterns during network diffusion. For this purpose it would be useful to implement FFT [159, 160] and wavelet analyses [161, 162] of the oscillations of nodes of the networks. Several methods exist which demonstrate how analysis of signal transmission may work in neuronal networks [163–166]. Transmission of propagating signals can be analyzed in terms of spreading pattern exhibiting *cross networks communications* as well as a *straight forward transmission* with minimal interaction through a certain route of a connectome. At least network flow analysis [167] has been applied successfully for the Caenorhabditis elegans connectome. It should be mentioned that a more basic theory exists of how to model signal propagation in networks using *linear response theory* [168]. These propagation approaches may be applied to better understand the propagation of signals in non-lesioned control connectomes and connectomes with modulation of connections weights shaping demyelination processes in multiple sclerosis as well as loss of neuron populations in neurodegenerative diseases including Parkinson’s, Alzheimer’s, Huntington’s, Batten disease and amyotrophic lateral sclerosis.

## Conclusion

The three reaction diffusion models of Gierer-Meinhardt, Mimura-Murray, and Gray-Scott can be adapted in directed and edge weighted networks or connectomes of the nervous system. Consideration of Euclidean distances between nodes of the treated network in the diffusion processes was also realized. It was shown that the reaction diffusion systems retain their stability against noise. The comparison of the results of the reaction diffusion systems developed here with previously published studies, showed a qualitative agreement. The compound weights can be modulated during a diffusion process. Thus, it is possible to apply demyelination and remyelination processes as they occur in certain forms of multiple sclerosis in a reaction diffusion simulation. It was shown what effects periodic modulations of connection weights have on the oscillation of concentrations in network nodes during diffusion processes. The described implementation was done in *neuroVIISAS* and is directly executable on different operating systems. In summary, it can be stated that the three reaction diffusion models mimic the effects of demyelination through weight changes within the diffusion process and could be a promising tool to predict changes of connectivity of those regions which do not show obvious function changes.

## Supporting information

S1 Text Supporting information file containing tutorials and supplemental Figs A-BW. The Supporting Information is divided into several sections and is intended to make the use of the discussed RD models in *neuroVIISAS* comprehensible. For this purpose, a short introduction to *neuroVIISAS* is given in the first part of a tutorial. In the second part of the tutorial more detailed background information about the RD models is given. In the third part of the tutorial, complementary experiments to the main findings described in the article are presented. In the last section of the Supporting Information, graphical representations are shown to which references are given in the main body of this paper. They are used for comparison to the described experiments and for the consolidation of individual findings. The exemplar connectivity data (MS.brain) that can be loaded directly into neuroVIIAS and the source code (Reaction-diffusion.zip) are available on figshare (https://doi.org/10.6084/m9.figshare.21081418.v1).
(PDF)
