# Supplementary material for: Reaction-diffusion models in weighted and directed connectomes
Source: PLoS Comput Biol. 2022 Oct 28;18(10):e1010507. doi: 10.1371/journal.pcbi.1010507 (PMC9683624; doi:10.1371/journal.pcbi.1010507)
Supplement: S1 Text — The Supporting Information is divided into several sections and is intended to make the use of the discussed RD models in neuroVIISAS comprehensible. For this purpose, a short introduction to neuroVIISAS is given in the first part of a tutorial. In the second part of the tutorial more detailed background information about the RD models is given. In the third part of the tutorial, complementary experiments to the main findings described in the article are presented. In the last section of the Supporting Information, graphical representations are shown to which references are given in the main body of this paper. They are used for comparison to the described experiments and for the consolidation of individual findings. The exemplar connectivity data (MS.brain) that can be loaded directly into neuroVIIAS and the source code (Reaction-diffusion.zip) are available on figshare (https://doi.org/10.6084/m9.figshare.21081418.v1). (PDF) [file pcbi.1010507.s001.pdf]

Supporting information

A tutorial for network diffusion

The diffusion reaction models were implemented in the *neuroVIISAS* framework [1]. Due to the generic approach and integration of digital atlasing, neuroontology generation, visual analytics as well as structural and dynamic network analysis it was necessary to describe configurations, functions and workflows in a help system inside the framework. So far a detailed description of how to use the RD models in the context of directed and weighted networks and dynamic network configurations is not available in the help system. Thus, the following tutorial part 2 should give an overview of theoretical foundations of network diffusion and serves as a step-by-step guide for applying the RD models.

In the **first part of the tutorial**, a workfkow is described in order to start *neuroVIISAS*, to configure the MS.brain dataset that was used in this investigation and to perform RD models for reproducing the findings in the subsection "Results".

In the **second part of the tutorial**, an introduction, theoretical foundation and implementation details of the RD models are provided. Within the subsection of implementation details examples of RD outcomes are shown.

In the **third part of the tutorial**, the results of non-weighted and weighted couplings of small graphs are compared and interpreted.

Tutorial part 1: Starting *neuroVIISAS* and loading the MS.brain data

neuroVIISAS should be started through a MS-Windows batch file (run.bat) or a Linux script file (run.sh) which are located in the installation directory. If a 32 bit JRE is installed and a 64 bit *neuroVIISAS*.jar is started from a desktop item then the error information of the console will be not available and it appears that nothing happens. It is recommended to use a 64 bit JRE for a 64 bit *neuroVIISAS*.jar. The run.sh contains a starting command which can be adapted to the size of a connectome project and directly entered on the console:

```
/usr/java/jre1.8.0_66/bin/java -splash:Images/logo_splash.png
-jar -Xms2000m -Xmx10000m -Xss24M neuroVIISAS.jar
```

The command consists of the path to the Java Run Time Environment (JRE) and the flags -Xms, -Xmx and -Xss for starting the Java Virtual Machine (JVM). -Xms defines the amount of memory up to the maximum of -Xmx memory for the *neuroVIISAS* Java process which is relatively large in the example above: 10000Mb. Also, -Xss is relatively large and assigns the stack size with 24 Mb which is important for huge hierarchies of connectome regions. If *neuroVIISAS* is started without these parameters and a project file with larger sizes of regions and connections is loaded then it is probable that error messages will occur in the console window and data files are not loaded.

After loading *neuroVIISAS* the main window appears (Fig A). The MS.brain project file can be loaded by clicking on "File" in the main menu bar (Fig A-E) and selecting "Open project". The references.bib can be loaded in the "Settings" menu and "Change project settings" (Clicking on the button "Choose new bibtex file") allows to specify a reference database, however, it is not mandatory for quantitative rather than qualitative connectome analysis.

Following a click on the "Analysis" menu in the main window menu bar the "Advanced connectome analysis" item must be selected. In the bottom row of the window a list of basic matrix, table and analyses representations is displayed in the form of "Tabs" (Fig C-H). After opening the "Advanced connectome analysis" window the "Adjacency Matrix" tab is active, however, an empty analysis window will be displayed (Fig B).

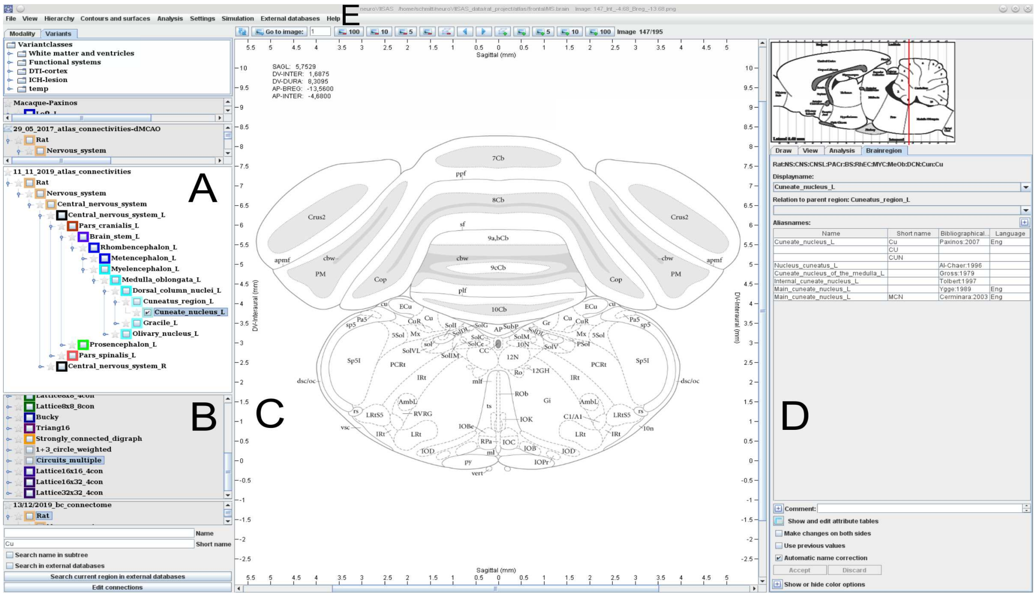

Fig A. *neuroVIISAS* start window. A) The subwindow with white background color indicates the active connectome project which is the MS.brain data with highlighted "Cuneate nucleus" of the left side. B) Is a further project which contains different circuits (test.brain data set). A and B are located in the first third of the main window. In this part, all projects are shown. They can be activated by clicking into the subwindows. C) indicates the center subwindow which displays stacks of histological, MRI or atlas images. D) is located in the right third of the main window. Longnames, abbreviations and further information of particular regions of a connectome are displayed. E) Is the main menu bar. "File" has to be clicked to load a *.brain project file and "Analysis" has to be selected to open a submenu for the connectome analysis.

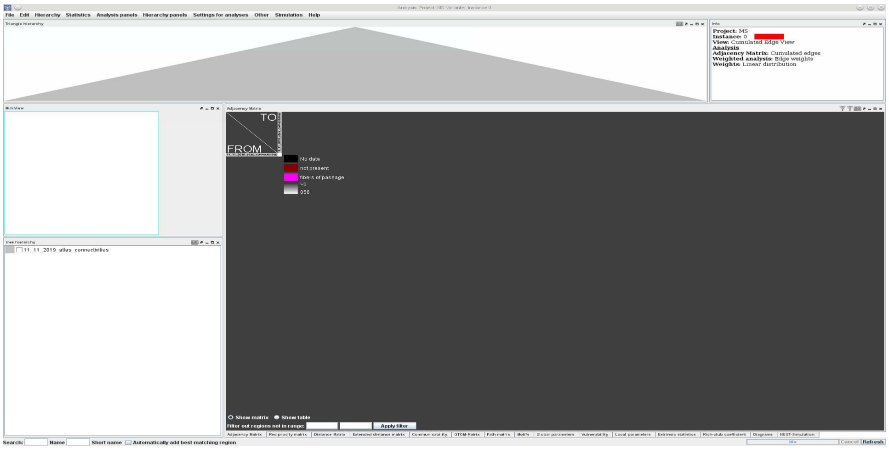

Fig B. The analysis window. Initially the analysis window is empty and does not display any data.

The hierarchy or a list of regions of a connectome can be opened in several ways. The straightforward method is to repeatedly press the “+” key until the lowest level of the hierarchy of regions of the connectome has been reached (Fig C-A). Then the connections between these regions can be displayed by pressing the “Enter” key or by clicking on the “Refresh” button in the lower right corner of the window (Fig C-H). A default display of connections (Fig C-B) with a navigation window (Fig C-C) appears. In the default display, the number of connections between pairs of regions that were found in the tract tracing research literature is presented. It can be enlarged by scrolling the mouse wheel. In order to change the matrix display, the “Settings” window must be opened by clicking on the “Settings” icon (Fig C-E) which is an important feature for most other matrix representations as well. The “Settings” window (Fig C-F) can be scrolled down until the radio button “Average weight / Most frequent weight” appears (beside the “F” in Fig C). Thereafter, the ordinal coded average weights are represented in a green shaded default color palette (Fig D).

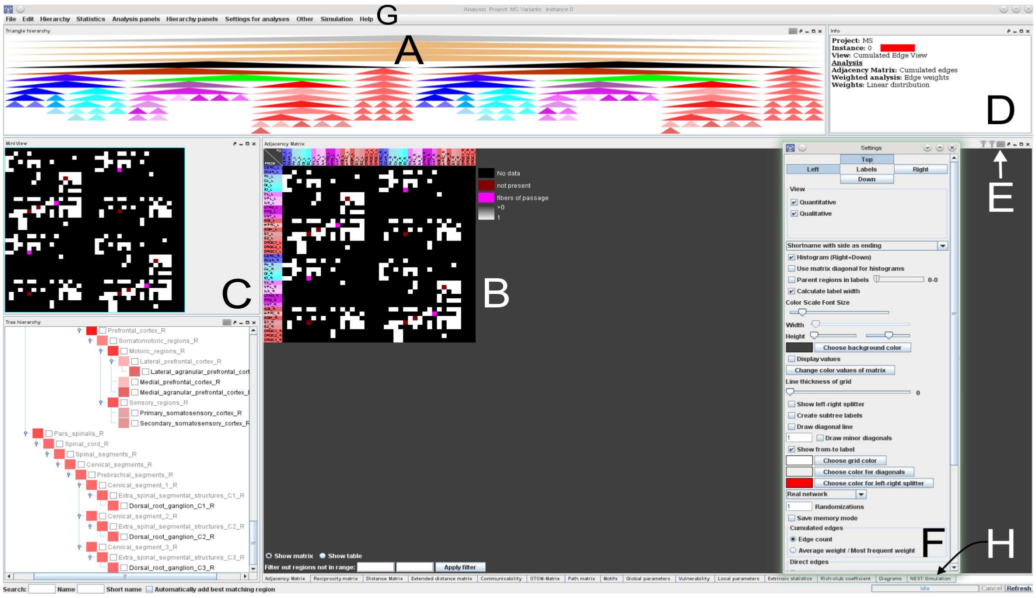

Fig C. A configured connectome in the analysis window. Hierarchically organized regions of a connectome can be extended by pressing repeatedly the ”+“ key. After reaching a hierarchy level which contains the nodes or regions with connections the ”Enter“ key must be pressed to computed the adjacency matrix (default view) A) The extended triangle representation of the hierarchy of regions of the connectome. B) The default representation of connections (edge count matrix). C) The navigator window. D) The data information window. E) The settings button must be clicked to open the ”Settings“ window F. By clicking in the ”Settings“ window F on ”Average weight / ...“ the adjacency matrix representation will change. H) is the tabular bar with the ”Refresh“ button in the lower right corner.

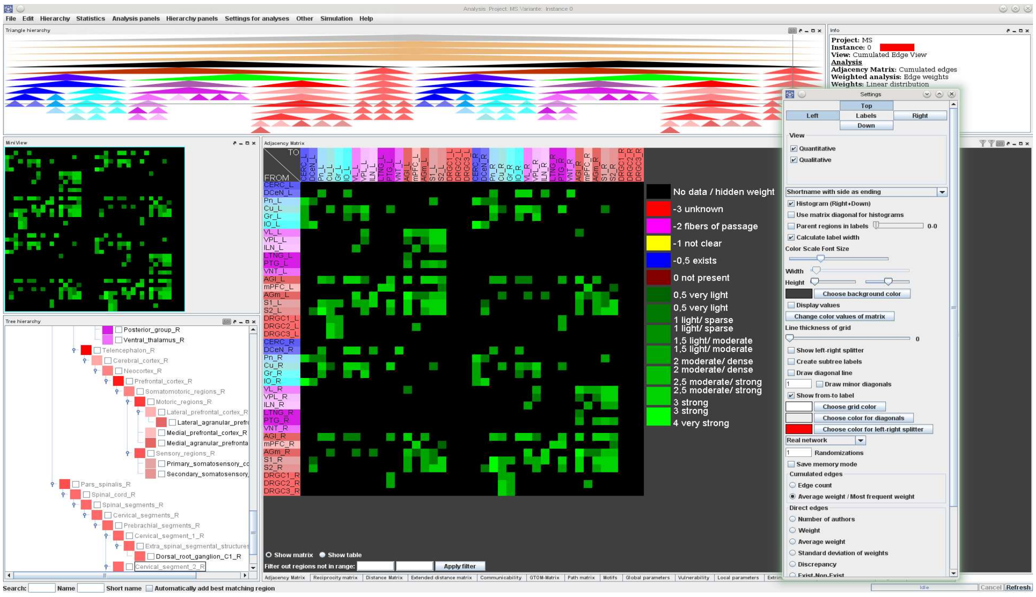

Fig D. The average weight view in the analysis window. After selecting "Average weight / Most frequent weight" from the "Settings" window the adjacency matrix will be directly updated. The mouse wheel enlarges the adjacency matrix.

In order to apply a RD model to the selected regions and connections as indicated in the "Adjacency matrix" tab "Analysis panels" in the menu bar (Fig C-G) and "Simulation" must be clicked (Fig E). Thereafter, a new empty Tab will be generated and automatically opened (Fig F). By clicking on the "Settings" icon in the RD model window (see also Fig C-E) the parameters of the Gierer-Meinhardt model can be defined. After setting the parameters, the "Refresh" button must be clicked to perform the RD process. After finishing the RD process the table of results of the GM simulation is filled with average activation values and spike count values. In the MiniView the coactivation matrix is shown which can be enlarged by clicking on the radio button "Show coactivation matrix". To obtain the display of frequency space of the GM simulation the button "Function" must be clicked. In the window a diagram is shown (Fig G) were all concentrations of regions are initially represented by dots. By choosing a linear or cubic interpolation the functions are displayed as curves. A single function or a set of functions can be displayed by selecting the regions in the results table.

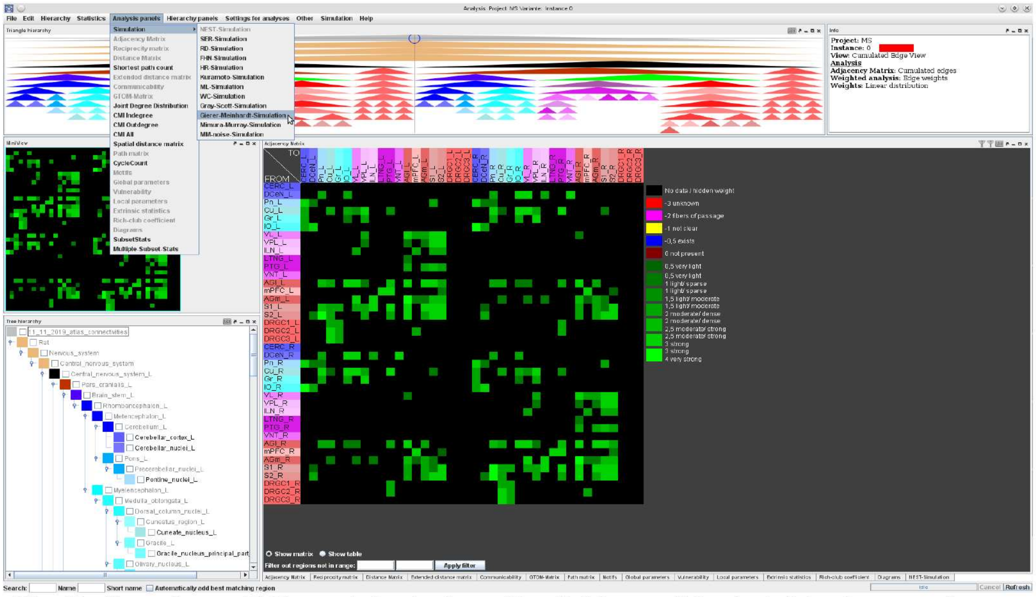

Fig E. Opening a RD model window. By clicking on "Analysis" in the menu bar followed by clicking on "Simulation" (then pull mouse within the same row of the menu). In the simulation menu the item "Gierer-Meinhardt" has been selected.

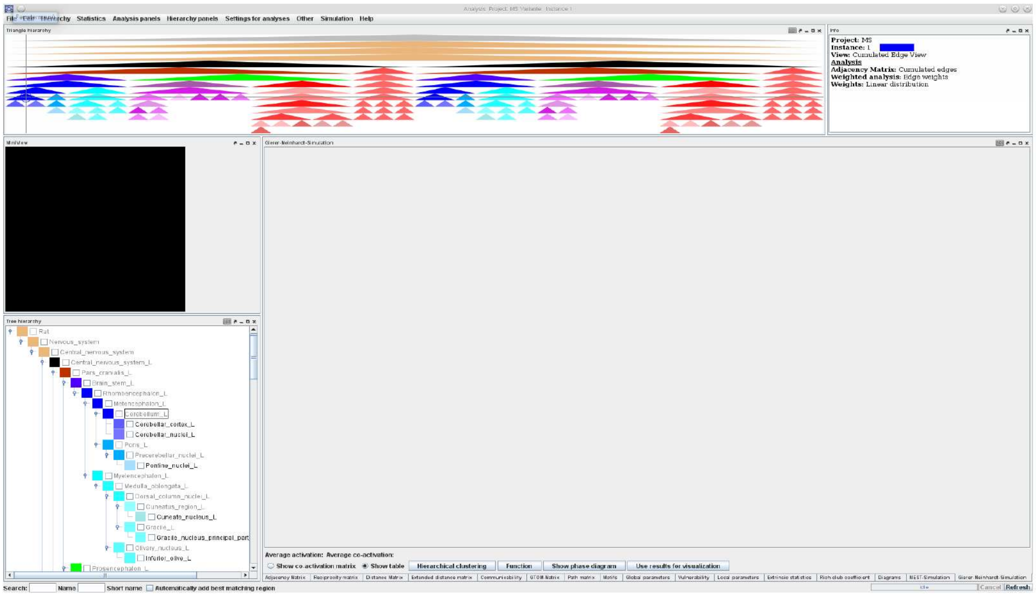

Fig F. The empty RD model window. By clicking on the "Settings" icon the dialogue for defining the RD model will be opened (Fig G).

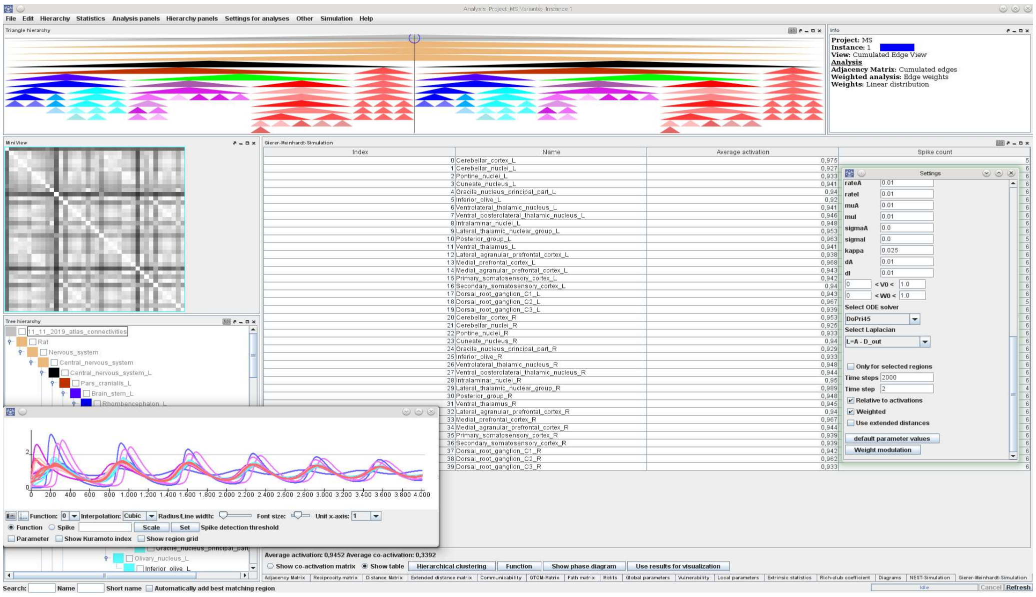

Fig G. The RD model window with the "Settings" menu. The "Settings" window has been reduced and scrolled down. Some default parameters of the Gierer-Meinhardt model were modified ($rateI$, muI, dI). Randomized initial values between 0 and 1 (V_0, W_0) were used. The DoPri45 solver was applied and Laplacian $L = A - D_{out}$. Time steps were set to 2000 and one time step has the size of 2. The connection weights were used by checkmarking "Weighted" and a normalization of the coactivation matrix was applied ("Relative to activation").

In order to apply transformed connection weights it is necessary to click on “Settings for analyses” in the menu bar of the main analysis window and then selecting “Settings for graph analyses” (Fig H). In the window for settings of weights and the applications (Fig I) the button “Logarithmic distribution 2” must be pressed (Fig J). Thereafter, the window can be closed and it possible to directly proceed with preceding parameters of the GM model to perform a new simulation by pressing the “Refresh” button. The results are displayed in the table or by pressing the “Function” button (Fig K). The concentration changes of each region are shown over 2000 time steps of the size 2. The values were divided by 10 (“Unit x-axis“). A cubic interpolation was applied and the Kuramoto order parameter was checkmarked (”Show Kuramoto index“).

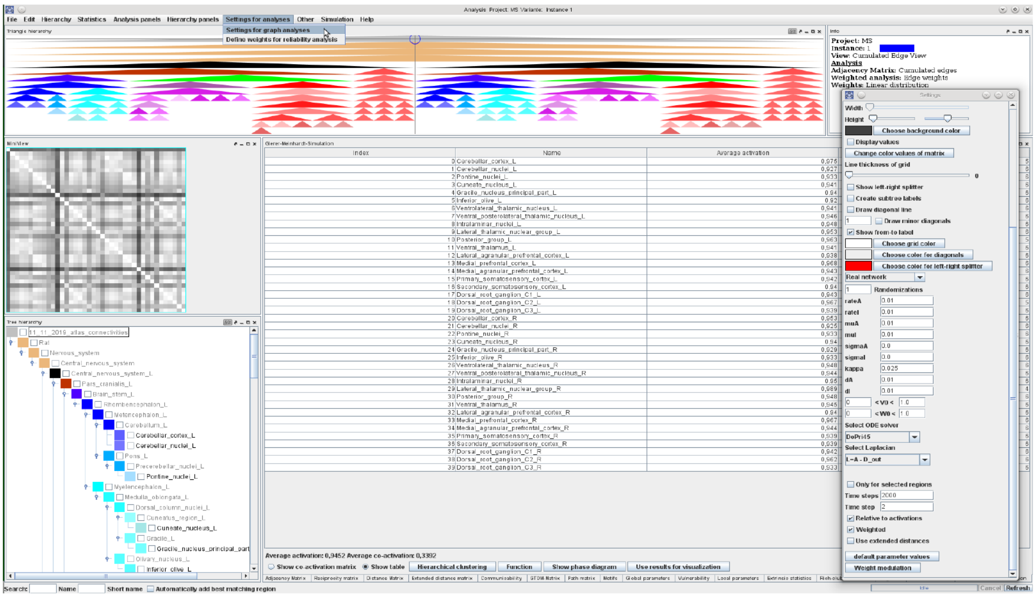

Fig H. Opening the dialogue to change connection weights. The ”Settings for analyses“ in the menu bar of analysis main window must be clicked to open the ”Settings for graph analyses“ window.

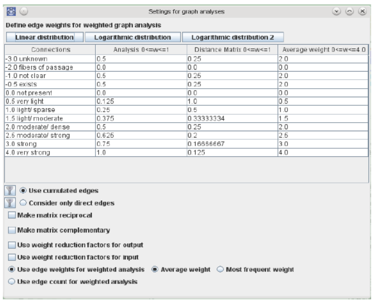

Fig I. The ”Settings for graph analysis“ window. After opening the window for defining connection weights the default configuration is shown. The ordinal scaled connection weights are active.

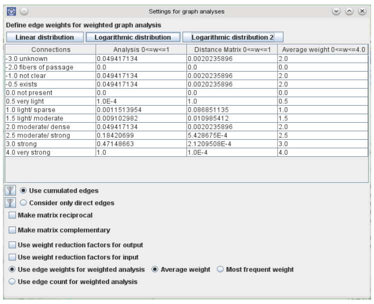

Fig J. Changing connection weights. To transform the connection weights it is necessary to click on the "Logarithmic distribution 2“ button. The transformed values of weights are displayed in the table.

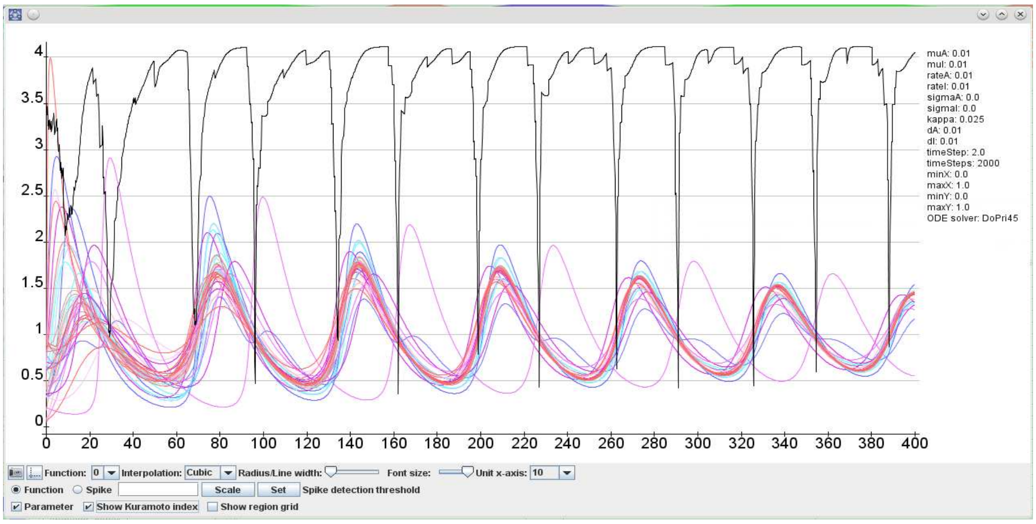

Fig K. Applying transformed connection weights in a GM process. The concentrations of the GM process for logarithmically transformed connection weights is displayed. The black curve represents the Kuramoto order parameter.

In the next step, the weights of the output connections of the left dorsal root ganglia of the cervical segments 1-3 will be reduced. In the "Settings“ window where GM model parameters were adopted the button "Weight modulation“ must be pressed. A new interval for the change of weights need to be defined by clicking on "Add interval“. In the message window "Input“ the end of the x-interval (iteration) has to be set (3600) and in the following message window the end of the y-interval must be defined (0.1) (Fig L). Now the cosine function can be select from the List-window. As scale factors x=1.0 and y=0.2 (amplitude damping) were used. Finally, 10 oscillations should be generated. The parameters are applied after pressing the "Accept“ button. Before, the "Apply to selected regions“ button is pressed, the three regions in the table of results of the GM process have to be selected by clicking on the rows and holding the shift key. Thereafter, by pressing the "Apply to selected regions“ button the "Choose an option“ window appears were the "Output“ button should be pressed. Then, the weight modulation function is applied to the three selected regions, only (Fig M). The "Refresh“ button will perform the FM process with weight modulation of the three highlighted regions whereas all other regions will keep there original logarithmically transformed weights (if the "Function“ window does not appear after pressing the "Function“ button working

with a Linux OS/KDE it may be hidden behind other windows: Alt+Tab, then scroll through the list of windows). The results of the GM process with weight reduction of the first three left side DRG regions is displayed in the "Function" window (Fig N).

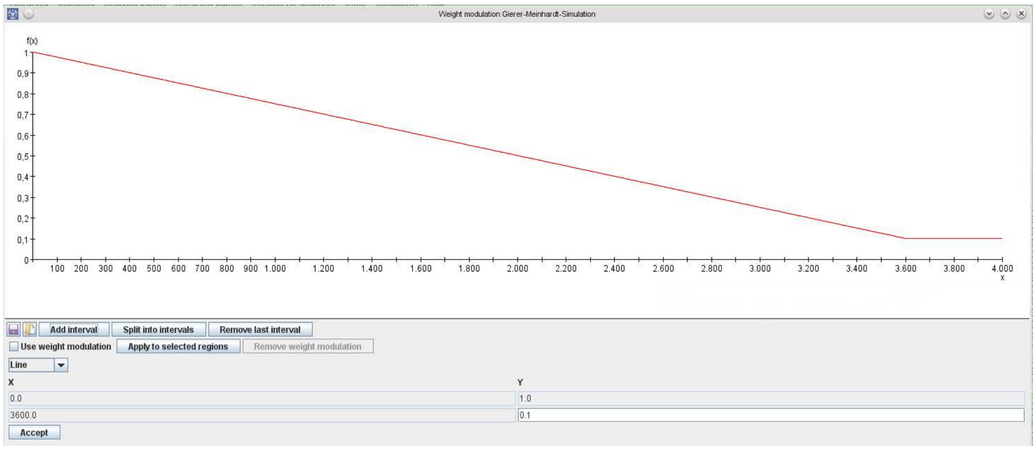

Fig L. Defining the a weight modulation function for a GM process. The interval of the x-axis and y-axis as well as the modulation function features are defined in this dialogue window.

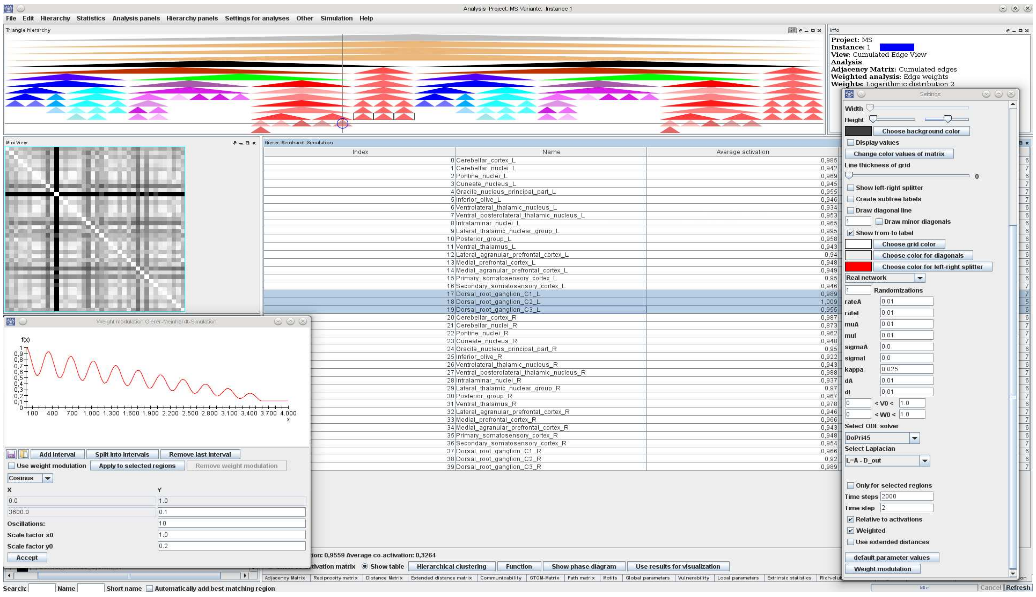

Fig M. The weight modulation function. The weight modulation function has to be assigned to a set of highlighted regions.

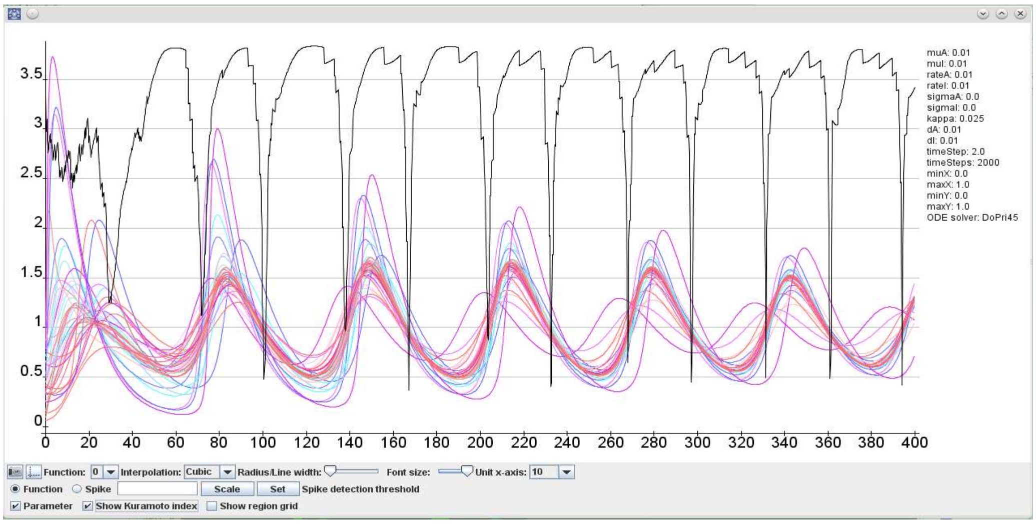

Fig N. Results of a GM process with a weight modulation function. The decrease of concentration and change of the Kuramoto order parameter can be seen in this diagram.

References

1. Schmitt O, Eipert P. neuroVIISAS: approaching multiscale simulation of the rat connectome. Neuroinformatics 2012; 10: 243-267.

Tutorial part 2: Reaction-diffusion systems (RD)

Introductory remarks

The phenomenon of diffusion was discovered two centuries ago by Graham and Fick [1, 2]. Fick found that diffusion flux is proportional to the negative of the concentration gradient of particles (*Fick law*). Diffusion can be observed in physics with regard to particles (particle diffusion), in chemistry and in biology (macromolecules, ions, cell organelles). Alan Turing [3] developed a generative concept of diffusion which helps to understand how stable patterns (*Turing stability*) evolve in artificial and natural systems [4–6]. Soon after Turing's discovery, the concept of diffusion was abstracted and carried forward to the field of epidemics. Diffusion helps to understand and predict the spread of infections in compartmental models of contagion like the SI (Susceptible-Infectious), SIS (Susceptible-Infectious-Susceptible) and SIR (Susceptible-Infectious-Recovered) models [7–9]. Proceeding from the diffusion of infections in epidemic research, diffusion has been applied to generate information flow in networks [10] in order to determine how the topology of a network or the pattern of connections between regions affects diffusion.

The diffusion equation can be used in a more technical context like the reduction of noise in biological and medical imaging to preserve the geometry of anatomical details [11]. However, the diffusion process itself is not used in this application to generate a dynamic process in irregular grids such as planar graphs, networks or digraphs. After all, the measurement of diffusion of water along plasma membranes and myelin sheaths of neurits is fundamental for *in vivo* and *non-invasively* diffusion tensor imaging (DTI) to visualize non-directed neuronal connections in living brains [12–17].

The microenvironment of brain tissue is structured by membranes and myelin or bundles of myelinated nerve fiber surfaces. These boundaries separate spatial compartments or phases through an interface that permits metastability and/or bistability of different ional and specific molecular concentration patterns. In these compartments, signals of neurons are transmitted by action potentials which are generated and propagated by ion fluxes or ion transport processes, respectively [18, 19]. Such *electro-diffusion* processes modeled (electrodiffusive Kirchhoff-Nernst-Planck framework) [18, 20, 21] at a microscopic level of neurons constitute a precondition for coding of memory, learning, motion control, vision and auditory information processing at the macroscopic level [22–24].

Many diffusion processes are known in biological, in particular, neuronal systems. *Intracellular diffusion* processes are fundamental in the cytoplasm and axoplasm [18, 25–28, 44]. *Intercellular diffusion* [18, 29–33] and glymphatic flow [34] in the central nervous system are necessary for molecular signaling and convection of metabolites. At the interface of intracellular and intercellular diffusion, a transition between these two compartments permits diffusion as well: the *transmembrane diffusion* [35, 36]. With regard to ional fluxes *diffusive coupling* has been conceptualized [18, 37–39]. Finally, *drift diffusion* models have been developed for imitating perceptual decision making [40, 41]. Taking the different diffusion processes occurring naturally in different compartments into account, a perception of *interacting diffusion networks* emerge. It has been shown that some of these diffusion processes can be modeled in a reproducible way [22, 42–45].

In view of the numerous realizations of diffusion processes in natural biological systems and compartments, it appears obvious to apply diffusion in neural networks. Models of reaction-diffusion (RD) adapted to networks can be used to investigate the spreading of information through networks or connectomes which may shape dynamic states that can be related to functional processes. It has been shown that dynamic models of brain communication have begun to create links between connectional

architectures and function. Furthermore, brains have the capacity to support a great diversity of dynamic patterns which are complex at a broad range of temporal frames to sustain a large number of competing functional demands. A large amount of different dynamic patterns has been considered as a functional repertoire of a network that allows flexibility across a broad range of cognitive functions [46].

Complex self-organized patterns, such as spreading pulses and fronts, stationary dissipative forms, rotating waves, and turbulences, are produced by reaction-diffusion systems [3, 47–49, 84]. Several reaction-diffusion models have been discovered to function on networks by interacting organisms occupying network nodes and diffusively transferring information across links [50–54]. Chemical reactors and networks of diffusively connected biological cells or areas can both be represented using reaction-diffusion models [51–54]. Self-organization analysis in complex networks is challenging, therefore it has been limited to non-equilibrium pattern development as synchronization [55–57] or epidemic spreading [58–60]. Changes in the diffusion constants of activators and inhibitors destabilize the uniform state of a system, resulting in the spontaneous formation of rhythmic patterns (Turing patterns) in chemical processes, biological morphogenesis, and ecosystems, according to Turing [3]. Turing instabilities and complicated non-equilibrium self-organization can also develop in networks [51]. In mathematical frameworks, network instability have been studied further [52–54]. However, their research was limited to regular lattices [51, 52] and small networks [53, 54].

Diffusion models have been shown to be effective in identifying functional units [61, 62] as well as forecasting statistical interdependence (functional connectivity) among distant neural time courses [63, 64]. *Network diffusion*, also known as *graph diffusion* [65, 66], has been effectively applied to network modularity analysis [67] and dementia disease [68] progression modeling.

Similar random walk and diffusion models [61, 65] were investigated and compared with the FitzHugh Nagumo (FHN) excitable neuron and susceptible-excited-refractory dynamic cycle (SER) models [69], a reaction-diffusion approach [4, 70] and a generative coactivation pattern in excitable neural networks [71]. One hypothesis standing behind dynamic modeling in networks is that connectome topology shapes, organizes and constrains dynamic processes [70]. By adopting a one-dimensional reaction-diffusion (RD) system [4] it could be shown that this model can produce stable patterns in coactivation matrices [70]. These promising results lead to further experiments using stochastic versions of two well-established computational models of brain activity. These are the excitable FitzHugh-Nagumo neuron and SER models which reproduce some patterns of empirical functional connectivity as measured in resting-state fMRI experiments [72, 73]. The deterministic SER model is a three-state cellular automaton model which is capable of generating excitable dynamics. The FHN model is similar to diffusion models and generates more distinct dynamic patterns in modular networks than the SER and RD models [69].

RD and SER are not built for incorporating further features of a connectome like connection weights, Euclidean distances and volume estimates of regions or nodes. Furthermore, originally RD and SER were not applied to digraphs with reciprocal edges. Here, the FHN model has been adapted to generate oscillatory dynamics on a weighted digraph.

Until now, reaction-diffusion models with varied reactions terms in the differential equations have allowed for better parametric creation of individual connectome topologies. In this study, the predator-prey and activator-inhibitor models were used.

Analyzing reaction-diffusion models on connectome architectures provides insight in the pattern forming capabilities and, hence, the feasible collective modes, of such architectures. Here we first illustrate, using simple, generic network architectures, how

reaction-diffusion systems create sets of nodes with common dynamical behaviors, which cannot be trivially derived from the network architecture alone. We subsequently apply this approach to the spinal cord, brainstem, diencephalic and cortical connectivity of the mechanosensory pathways. This new approach - probing connectomes with reaction-diffusion models - is fully integrated in *neuroVIISAS*. A detailed tutorial is provided as Supplementary Information.

The weights of neural connections in a connectome are used to calculate the strength of connections between areas in reaction-diffusion models. The number of nerve fibers that have included a tract-tracing substance or an estimate of the number of traced nerve fibers are encoded by the weights of connections in tract-tracing investigations. As a result, estimations of connection weights do not represent the mean thickness of myelin sheaths studied using transmission electron microscopy or other methods. Most tract-tracing investigations use estimates of link weights or densities, which are divided into three categories: weak, moderate, and strong. Furthermore, connections can be defined without any classifications or in weight categories such as "weak to moderate" or "very strong". Schwanke et al. [74] provides an overview of all categories, their interpretation, relationships, and comparisons. The strength of a connection that facilitates transmission of an electrochemical signal across parts of the nervous system may be described in terms of a relationship between the weights of connections and their functional mean. More information may be sent if the connection strength is high. The weights of connection can be linked to neuropathological alterations in nerve fibers, such as those seen in Multiple Sclerosis (MS), when demyelination and axon degradation occur. In this situation, the connection weights will be lowered. The ordinal scaled estimates were always logarithmically converted if connection weights were utilized in this investigation.

The predator-prey model of Lotka-Volterra [75–79, 84] is one frequently used differential equation model to investigate interactions between species for understanding mechanisms of pattern formation. Reaction-diffusion systems modeling predator-prey interactions show a rich spatiotemporal dynamic (oscillatory behavior, spatial pattern developments). Some examples are the predator-prey system of the Segel-Jackson model [96, 97], the Leslie-Gower model [80] and the diffusive predator-prey system with delay effect [81, 82].

Mimura-Murray presented an expanded version of Lotka-predator-prey Volterra's model [83–85], which has several benefits over the original idea. As a result, the *Mimura-Murray model* (MM) looks to be promising for network diffusion adaptation.

Gierer and Meinhardt Gierer and Meinhardt (GM) devised an activator-inhibitor reaction-diffusion model [86–88]. It comprises of a reaction term with activator and inhibitor parameters that may be used to construct various weighted digraph oscillation patterns. As a result, the GM model looks to be a promising contender for network diffusion applications. Finally, the Gray-Scott model (GS) has been modified to network diffusion. The GS is a standard mathematical model for isothermal autocatalytic reaction with another type of reaction component in the differential equation [89–95].

The goal of this research was to look at reaction-diffusion models in terms of weighted digraphs and diffusion distances. Synthetic randomized directed networks with maintained edges and nodes were created using data from the connectome of the rat nervous system. In order to develop more realistic coupled dynamic models based on empirical data, the reaction-diffusion models of Gierer-Meinhardt [86–88], Mimura-Murray [83–85], and Gray-Scott [89–95] were adapted to weighted digraphs while taking Euclidean distances between nodes into account.

A second aim of this investigation was the analysis of pattern forming in such a way that the reaction-diffusion models generate sets of nodes with common dynamical behavior.

Another goal of this research was to look at the consequences of changing connection weights in the reaction-diffusion process, since this might be a starting point for modeling neurodegenerative disease development [98–103]. Particularly those types of neurodegenerative disorder that affect neural connections rather than gray matter or neuronal perikarya. Multiple sclerosis is a demyelinating condition that progresses in a wide range of temporal and geographic patterns [104–113]. The framework used here allows for the definition of sets of source nodes or regions and sets of target nodes of interest embedded in a large connectome context in order to systematically investigate signal propagation or information diffusion through highly complex connectional architectures with precisely defined changes in connection weights. As a result, our simulation environment looks to be a good starting point for investigating changes in network properties in morphopatholgical neurological disorders and their influence on dynamic pattern alterations in a consistent and repeatable manner.

References

1. Graham T. A short account of experimental researches on the diffusion of gases through each other, and their separation by mechanical means. Quart J Sci Lit Art. 1829; 27: 74-83.

2. Fick A. Über Diffusion. Pogg Ann Phy. 1855; 94: 59-86.

3. Turing AM. The chemical basis of morphogenesis. Phil Trans R Soc Lond B. 1952; 237: 37-72.

4. Young DA. A local activator-inhibitor model of vertebrate skin patterns. Math Biosci. 1984; 72: 51-58.

5. Howard J, Grill SW, Bois JS. Turing's next steps: the mechanochemical basis of morphogenesis. Nat Rev Mol Cell Biol. 2011; 12: 392-398.

6. Brinkmann F, Mercker M, Richter T, Marciniak-Czochra A. Post-Turing tissue pattern formation: Advent of mechanochemistry. PLoS Comput Biol. 2018; 14: e1006259.

7. Bailey N. The mathematical theory of infectious diseases and its applications. London, Charles Griffin and Company Ltd.

8. Keeling M, Rohani P. Modeling infectious diseases in humans and animals. Princeton University Press.

9. Newman MEJ. Networks: an introduction. Oxford, Oxford University Press.

10. Meier J, Zhou X, Hillebrand A, Tewarie P, Stam CJ, Van Mieghem P. The epidemic spreading model and the direction of information flow in brain networks. Neuroimage. 2017; 152: 639-646.

11. Perona P, Malik J. Scale-space and edge-detection using anisotropic diffusion. IEEE Trans. Pattern Anal Mach Intell. 1990; 12: 629-639.

12. Le Bihan D, Breton E. Imagerie de diffusion in-vivo par résonance magnétique nucléaire. C R Acad Sci. 1985; 301: 1109-1112.

13. Merboldt K, Hanicke W, Frahm, J. Self-diffusion NMR imaging using stimulated echoes. J Mag Res. 1985; 64: 479-486.

14. Taylor DG, Bushell MC. The spatial mapping of translational diffusion coefficients by the NMR imaging technique. Phys Med Biol. 1985; 30: 345-349.

15. Ghosh A, Deriche R. A survey of current trends in diffusion MRI for structural brain connectivity. J Neural Eng. 2016; 13: 011001.

16. Steltjes B, Brunner RM, Fritzsche KH, Laun FB. Diffusion tensor imaging: Introduction and atlas.. Springer-Verlag, Berlin, Heidelberg, 2013.

17. Mori S, Tournier J-D. Introduction to diffusion tensor imaging and higher order methods. Elsever Science, 2014.

18. Keener J, Sneyd J. Mathematical physiology. I: Cellular physiology. 2nd ed., Springer, 2009.

19. Hodgkin AL, Huxley AF. A quantitative description of membrane current and its application to conduction and excitation in nerve. J Physiol. 1952; 117: 500-544.

20. Coggan JS, Prescott SA, Bartol TM, Sejnowski TJ. Imbalance of ionic conductances contributes to diverse symptoms of demyelination. Proc Natl Acad Sci U S A. 2010; 107: 20602-20609.

21. Maex R. On the Nernst-Planck equation. J Integr Neurosci. 2017; 16: 73-91.

22. Xiang ZX, Liu GZ, Tanf CX, Yan LX. A model of ion transport processes along and across the neuronal membrane. J Int Neuro. 2017; 16: 33-55.

23. Halnes G, Mäki-Marttunen T, Keller D, Pettersen KH, Andreassen OA, Einevoll GT. Effect of ionic diffusion on extracellular potentials in neural tissue. PLoS Comput Biol. 2016; 12: e1005193.

24. Solbrå A, Bergersen AW, van den Brink J, Malthe-Sørenssen A, Einevoll GT, Halnes G. A Kirchhoff-Nernst-Planck framework for modeling large scale extracellular electrodiffusion surrounding morphologically detailed neurons. PLoS Comput Biol. 2018; 14: e1006510.

25. Najac C, Branzoli F, Ronen I, Valette J. Brain intracellular metabolites are freely diffusing along cell fibers in grey and white matter, as measured by diffusion-weighted MR spectroscopy in the human brain at 7 T. Brain Struct Funct. 2016; 221: 1245-1254.

26. Fonkeu Y, Kraynyukova N, Hafner AS, Kochen L, Sartori F, Schuman EM et al. How mRNA localization and protein synthesis sites influence dendritic protein distribution and dynamics. Neuron. 2019; 103: 1109-1122.

27. Zhang Y, Tzingounis AV, Lykotrafitis G1,. Modeling of the axon plasma membrane structure and its effects on protein diffusion. PLoS Comput Biol. 2019; 15: e1007003.

28. Palti Y, Gold R, Stämpfli R. Diffusion of ions in myelinated nerve fibersx. Biophys J. 1979; 25: 17-31.

29. Syková E, Nicholson C. Diffusion in brain extracellular space. Physiol Rev. 2008; 88: 1277-12340.

30. Veraart J, Fieremans E, Novikov DS. On the scaling behavior of water diffusion in human brain white matterx. Neuroimage. 2019; 185: 379-387.

31. Holter KE, Kehlet B, Devor A, Sejnowski TJ, Dale AM, Omholt SW et al. Interstitial solute transport in 3D reconstructed neuropil occurs by diffusion rather than bulk flow. Proc Natl Acad Sci U S A. 2017; 114: 9894-9899.

32. Magdoom KN, Brown A, Rey J, Mareci TH, King MA, Sarntinoranont M. MRI of whole rat brain perivascular network reveals role for ventricles in brain waste clearance. Sci Rep. 2019; 9: 11480.

33. Rodriguez-Grande B, Konsman JP. Gas diffusion in the CNS. J Neurosci Res. 2018; 96: 207-218.

34. Jessen NA, Munk AS, Lundgaard I, Nedergaard M. The glymphatic system: A beginner's guide. Neurochem Res. 2015; 40: 2583-2599.

35. Jourdain P, Pavillon N, Moratal C, Boss D, Rappaz B, Depeursinge C et al. Determination of transmembrane water fluxes in neurons elicited by glutamate ionotropic receptors and by the cotransporters KCC2 and NKCC1: a digital holographic microscopy study. J Neurosci. 2011; 31: 11846-11854.

36. Heine M. Surface traffic in synaptic membranes. Adv Exp Med Biol. 2012; 970: 197-219.

37. Durand DM, Park EH, Jensen AL. Potassium diffusive coupling in neural networks. Philos Trans R Soc Lond B Biol Sci. 2010; 365: 2347-2362.

38. Durand DM, Park EY. Diffusive coupling can induce synchronized periodic activity in neural networks. Conf Proc IEEE Eng Med Biol Soc. 2008; 2008: 3677-3678.

39. Park EH, Feng Z, Durand DM. Diffusive coupling and network periodicity: a computational study. Biophys J. 2008, 95: 1126-1137.

40. Fard PR, Park H, Warkentin A, Kiebel SJ, Bitzer S. A bayesian reformulation of the extended drift-diffusion model in perceptual decision making. Front Comput Neurosci. 2017; 11: 29.

41. Sun P, Landy MS. A two-stage process model of sensory discrimination: An alternative to drift-diffusion. J Neurosci. 2016; 36: 11259-11274.

42. Blackwell KT. An efficient stochastic diffusion algorithm for modeling second messengers in dendrites and spines. J Neurosci Methods. 2006; 157: 142-153.

43. Kalantzis G. Hybrid stochastic simulations of intracellular reaction-diffusion systems. Comput Biol Chem. 2009; 33: 205-215.

44. Lagache T, Jayant K, Yuste R. Electrodiffusion models of synaptic potentials in dendritic spines. J Comput Neurosci. 2019; 47: 77-89.

45. Newton AJH, McDougal RA, Hines ML, Lytton WW. Using NEURON for Reaction-Diffusion Modeling of Extracellular Dynamics. Front Neuroinform. 2018; 12: 41.

46. Deco G, Jirsa VK, McIntosh AR. Resting brains never rest: computational insights into potential cognitive architectures. Trends Neurosci. 2013; 36: 268-274.

47. Prigogine I, Lefever R. Symmetry breaking instabilities in dissipative systems. J Chem Phys. 1968; 48: 1695-1700.

48. Castets V, Dulos E, Boissonade J, De Kepper P. Experimental evidence of a sustained standing Turing-type nonequilibrium chemical pattern. Phys Rev Lett. 1990; 64: 2953-2956.

49. Ouyang, Q, Swinney HL. Transition from an uniform state to hexagonal and striped Turing patterns. Nature 1991; 352: 610-612.

50. Barrat A, Barthélemy M, Vespignani A. Dynamical processes on complex networks. Cambridge Univ. Press, 2008.

51. Othmer HG, Scriven LE. Instability and dynamic pattern in cellular networks. J Theor Biol. 1971; 32: 507-537.

52. Othmer HG, Scriven LE. Nonlinear aspects of dynamic pattern in cellular networks. J Theor Biol. 1974; 43: 83-112.

53. Horsthemke W, Lam K, Moore PK Network topology and Turing instability in small arrays of diffusively coupled reactors. Phys Lett A. 2004; 328: 444-451.

54. Moore PK, Horsthemke W. Localized patterns in homogeneous networks of diffusively coupled reactors. Physica D. 2005; 206: 121-144.

55. Ichinomiya T. Frequency synchronization in a random oscillator network. Phys Rev E, 2004; 70, 026116.

56. Boccaletti S, Latora V, Moreno Y, Chavez M, Hwang D-U. Complex networks: Structure and dynamics. Phys Rep. 2006; 424: 175-308.

57. Arenas A, Díaz-Guilera A, Kurths J, Moreno Y, Zhou C. Synchronization in complex networks. Phys Rep. 2008; 469: 93-153.

58. Pastor-Satorras R, Vespignani A. Epidemic spreading in scale-free networks. Phys Rev Lett. 2001; 86: 3200-3203.

59. Colizza V, Pastor-Satorras R, Vespignani A. Reaction-diffusion processes and metapopulation models in heterogeneous networks. Nature Phys. 2007; 3: 276-282.

60. Colizza V, Vespignani A. Epidemic modeling in metapopulation systems with heterogeneous coupling pattern: Theory and simulations. J Theor Biol. 2008; 251: 450-467.

61. Betzel RF, Griffa A, Avena-Koenigsberger A, Goñi J, Thiran J-P, Hagmann P et al. Multi-scale community organization of the human structural connectome and its relationship with resting-state functional connectivity. Netw Sci. 2013; 1: 353-373.

62. Delvenne J-C, Yaliraki SN, Barahona M. Stability of graph communities across time scales. Proc Natl Acad Sci USA. 2010; 107: 12755-12760.

63. Goñi J, Avena-Koenigsberger A, Velez de Mendizabal N, van den Heuvel MP, Betzel RF, Sporns O. Exploring the morphospace of communication efficiency in complex networks. PLoS One. 2013; 8: e58070.

64. Mišić B, Betzel RF, Nematzadeh A, Goñi J, Griffa A, Hagmann P et al. Cooperative and competitive spreading dynamics on the human connectome. Neuron. 2015; 86: 1518-1529.

65. Abdelnour F, Voss HU, Raj A. Network diffusion accurately models the relationship between structural and functional brain connectivity networks. Neuroimage. 2014; 90: 335-347.

66. Abdelnour F, Dayan M, Devinsky O, Thesen T, Raj A. Functional brain connectivity is predictable from anatomic network's Laplacian eigen-structure. Neuroimage. 2018; 172: 728-739.

67. Nematzadeh A, Ferrara E, Flammini A, Ahn Y-Y. Optimal network modularity for information diffusion. Phys Rev Lett. 2014; 113: 088701.

68. Raj A, Kuceyeski A, Weiner M. A network diffusion model of disease progression in dementia. Neuron 2012; 73: 1204-1215.

69. Messé A, Hütt MT, König P, Hilgetag CC. A closer look at the apparent correlation of structural and functional connectivity in excitable neural networks. Sci Rep. 2015; 5: 7870.

70. Hütt MT, Kaiser M, Hilgetag CC. Perspective: network-guided pattern formation of neural dynamics. Philos Trans R Soc Lond B Biol Sci. 2014; 369(1653).

71. Messé A, Hütt MT, Hilgetag CC. Toward a theory of coactivation patterns in excitable neural networks. PLoS Comput Biol. 2018; 14: e1006084.

72. Haimovici A, Tagliazucchi E, Balenzuela P, Chialvo DR. Brain organization into resting state networks emerges at criticality on a model of the human connectome. Phys Rev Lett. 2013; 110: 178101.

73. Messé A, Rudrauf D, Benali H, Marrelec G. Relating structure and function in the human brain: relative contributions of anatomy, stationary dynamics, and non-stationarities. PLoS Comput Biol. 2014; 10: e1003530.

74. Schwanke S, Jenssen J, Eipert P, Schmitt O. Towards differential connectomics with NeuroVIISAS. Neuroinformatics. 2019; 17: 163-179.

75. Lotka AJ. Elements of Physical Biology. Williams and Wilkins, Baltimore, 1925.

76. Kazarinov N, van den Driessche P. A model predator - prey systems with functional response. Math Biosci. 1978; 39: 125-134.

77. Medvinsky AB, Petrovskii SV, Tikhonova IA, Malchow H, Li BL. Spatiotemporal complexity of plankton and fish dynamics. SIAM Rev. 2002; 44: 311-370.

78. Rosenzweig LM. Paradox of enrichment: destabilization of exploitation ecosystems in ecological time. Science. 1971; 171: 385-387.

79. Owen MR, Lewis MA. How predation can slow, stop or reverse a prey invasion. Bull Math Biol. 2001; 63: 655-684.

80. Gupta RP, Chandra P. Bifurcation analysis of modified Leslie - Gower predator-prey model with Michaelis-Menten type prey harvesting. J Math Anal Appl. 2013; 398: 278-295.

81. Zuo W, Wei J. Stability and Hopf bifurcation in a diffusive predator-prey system with delay effect. Nonlin Anal. 2013; 12: 1998-2011.

82. Rao F, Castillo-Chavez C, Kang Y. Dynamics of a diffusion reaction prey-predator model with delay in prey: Effects of delay and spatial components. J Math Anal Appl. 2018; 461: 1177-1214.

83. Mimura M, Murray JD. On a diffusive prey-predator model which exhibits patchiness. J Theor Biol. 1978; 75: 249-262.

84. Murray JD. Mathematical Biology, I. An Introduction, third edition. Interdiscip Appl Math. 2002; Vol. 17, Springer-Verlag, New York.

85. Nakao H, Mikhailov AS. Turing patterns in network-organized activator-inhibitor systems. Nat Phys. 2010; 6: 544-550.

86. Gierer A, Meinhardt H. A theory of biological pattern formation. Kybernetik. 1972; 12: 30-39.

87. Gierer A. Generation of biological patterns and form: Some physical, mathematical, and logical aspects. Progr Biophys Molec Biol. 1981; 37: 1-47.

88. Koch AJ, Meinhardt H. Biological pattern formation: from basic mechanisms to complex strucutres. Rev Mod Phys. 1994; 66: 1481-1507.

89. Pearson JE. Complex patterns in a simple system. Science. 1993; 261: 189-192.

90. Lee KJ, McCormick WD, Ouyang Q, Swinney HL. Pattern formation by interacting chemical fronts. Science. 1993; 261: 192-194.

91. Gray P, Scott SK. Autocatalytic reactions in the isothermal continuous stirred tank reactor: isolas and other forms of multistability. Chem Eng Sci. 1983; 38: 29-43.

92. Gray P, Scott SK. Autocatalytic reactions in the isothermal continuous stirred tank reactor: oscillations and instabilities in the system a + 2b $\rightarrow$ 3b, b $\rightarrow$ c. Chem Eng Sci. 1984; 39: 1087-1097.

93. Gray P, Scott SK. Sustained oscillations and other exotic patterns of behaviour in isothermal reactions. J Phys Chem 1985; 89: 22-32.

94. Gray P, Scott SK. Chemical oscillations and instabilities. Non-linear chemical kinetics. Clarendon Press, Oxford, 1994.

95. Hausenblas E, Randrianasolo TA, Thalhammer M. Theoretical study and numerical simulation of pattern formation in the deterministic and stochastic Gray-Scott equations. J Comp Appl Math 2019; 364: 112335.

96. Segel LA, Jackson JL. Dissipative structure: An explanation and an ecological example. J Theoret Biol. 1972; 37: 545-559.

97. Wang J, Wang Y. Bifurcation analysis in a diffusive Segel-Jackson model. J Math Anal Appl. 2014; 415: 2014-2016.

98. Zhou F, Zhuang Y, Gong H, Wang B, Wang X, Chen Q et al. Altered inter-subregion connectivity of the default mode network in relapsing remitting multiple sclerosis: a functional and structural connectivity study. PLoS One. 2014; 9: e101198.

99. Sbardella E, Tona F, Petsas N, Upadhyay N, Piattella MC, Filippini N et al. Functional connectivity changes and their relationship with clinical disability and white matter integrity in patients with relapsing-remitting multiple sclerosis. Mult Scler. 2015; 21: 1681-1692.

100. Ropero Peláez FJ, Taniguchi S. The gate theory of pain revisited: modeling different pain conditions with a parsimonious neurocomputational model. Neural Plast. 2016; 2016: 4131395.

101. Kotelnikova E, Kiani NA, Abad E, Martinez-Lapiscina EH, Andorra M, Zubizarreta I et al. Dynamics and heterogeneity of brain damage in multiple sclerosis. PLoS Comput Biol 2017; 13: e1005757.

102. Tommasin S, De Giglio L, Ruggieri S, Petsas N, Giannì C, Pozzilli C et al. Relation between functional connectivity and disability in Multiple sclerosis: a non-linear model. J Neurol. 2018; 265: 2881-2892.

103. Fleischer V, Radetz A, Ciolac D, Muthuraman M, Gonzalez-Escamilla G, Zipp F et al. Graph theoretical framework of brain networks in Multiple sclerosis: A review of concepts. Neuroscience. 2019; 403: 35-53.

104. Bando Y, Takakusaki K, Ito S, Terayama R, Kashiwayanagi M, Yoshida S. Differential changes in axonal conduction following CNS demyelination in two mouse models. Eur J Neurosci. 2008; 28: 1731-1742.

105. He Y, Dagher A, Chen Z, Charil A, Zijdenbos A, Worsley K et al. Impaired small-world efficiency in structural cortical networks in multiple sclerosis associated with white matter lesion load. Brain. 2009; 132: 3366-3379.

106. Guye M, Bettus G, Bartolomei F, Cozzone PJ. Graph theoretical analysis of structural and functional connectivity MRI in normal and pathological brain networks. MAGMA. 2010; 23: 409-421.

107. Wu GF, Alvarez E. The immunopathophysiology of multiple sclerosis. Neurol Clin. 2011; 29: 257-278.

108. Brennan KM, Galban-Horcajo F, Rinaldi S, O'Leary CP, Goodyear CS, Kalna G et al. Lipid arrays identify myelin-derived lipids and lipid complexes as prominent targets for oligoclonal band antibodies in multiple sclerosis. J Neuroimmunol. 2011; 238: 87-95.

109. Beecham AH, Patsopoulos NA, Xifara DK, Davis MF, Kemppinen A, Cotsapas C et al. Analysis of immune-related loci identifies 48 new susceptibility variants for multiple sclerosis. Nat Genet. 2013; 45: 1353-1360.

110. Gamboa OL, Tagliazucchi E, von Wegner F, Jurcoane A, Wahl M, Laufs H et al. Working memory performance of early MS patients correlates inversely with modularity increases in resting state functional connectivity networks. Neuroimage. 2014; 94: 385-395.

111. Tewarie P, Steenwijk MD, Tijms BM, Daams M, Balk LJ, Stam CJ et al. Disruption of structural and functional networks in long-standing multiple sclerosis. Hum Brain Mapp. 2014; 35: 5946-5961.

112. Brändle SM, Obermeier B, Senel M, Bruder J, Mentele R, Khademi M et al. Distinct oligoclonal band antibodies in multiple sclerosis recognize ubiquitous self-proteins. Proc Natl Acad Sci U S A. 2016; 113: 7864-7869.

113. Muthuraman M, Fleischer V, Kolber P, Luessi F, Zipp F, Groppa S. Structural Brain Network Characteristics Can Differentiate CIS from Early RRMS. Front Neurosci. 2016; 10: 14.

General form of a RD model and the Laplace operator

RD systems can be used to model the heterogeneous progression of *concentrations* of interacting substances on a two-dimensional domain. Instead of *concentration* the terms *substance*, *amount of substance* or *abundance* can be used. The general form of an n-dimensional reaction-diffusion system is:

$$\frac{\partial Y(\mathbf{x},t)}{\partial t} = f(\mathbf{x},t) + \Delta Y \tag{14}$$

with $Y \in \mathbb{R}^n$. The Laplace-operator is

$$\Delta Y = \sum_{i=1}^{n} \frac{\partial^2 Y}{\partial x_i^2}$$

which allows modeling of spatial diffusion of the two substances. Equation (14) is a partial differential equation which is transferred into a system of ordinary differential equation by spatial discretization. Here, are reaction terms considered which consist of two substances, respectively, the concentrations u and v. First, the case of a two-dimensional system is considered which will be extended to a reaction-diffusion system of networks. For the two spatial dimensions, the second derivatives are approximated by finite differences

$$\frac{\partial^2 u(x_0)}{\partial x^2} \approx \frac{u(x_0+\Delta x) - 2u(x_0) + u(x_0-\Delta x)}{(\Delta x)^2}$$
$$\frac{\partial^2 u(y_0)}{\partial y^2} \approx \frac{u(y_0+\Delta y) - 2u(y_0) + u(y_0-\Delta y)}{(\Delta y)^2}$$

It is assumed that the distance between two points on a regular lattice is equidistant, then $\Delta x = \Delta y = h$ and we obtain:

$$\begin{aligned}\Delta u(x_0,y_0) \approx & \frac{1}{h^2}\big(u(x_0+h,y_0) + u(x_0-h,y_0) \\ & - 4u(x_0,y_0) + u(x_0,y_0+h) + u(x_0,y_0-h)\big)\end{aligned}$$

To the nodes at the border, periodical boundary conditions are applied. In an example of a regular lattice of 4×4 nodes described by $u_i(t) = u(x_i, y_i, t)$ the following unknown functions are searched for:

$$u_1(t), ..u_{16}(t), v_1(t), .., v_{16}(t)$$

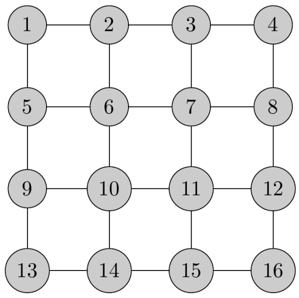

Fig O. Lattice with numbering of nodes. This is an example of a possibility to number consecutively the nodes of lattice. It is used in the following.

As an example, for the first upper left node (sub-index: 1) in the lattice (Fig O) we obtain

$$\begin{aligned}\frac{\partial u_1(t)}{\partial t} &= g(u_1(t), v_1(t)) + \frac{D_u}{h^2} \cdot (u_2(t) + u_5(t) + u_4(t) + u_{13}(t) - 4 \cdot u_1(t)) \\ \frac{\partial v_1(t)}{\partial t} &= f(u_1(t), v_1(t)) + \frac{D_v}{h^2} \cdot (v_2(t) + v_5(t) + v_4(t) + v_{13}(t) - 4 \cdot v_1(t)).\end{aligned} \tag{15}$$

A homogeneous linear differential equation with constant coefficients of first order is used here, for example

$$\begin{aligned}\frac{dy_1}{dt} &= 10y_1 - 5y_2 \\ \frac{dy_2}{dt} &= 6y_1 - y_2.\end{aligned}$$

The differential equation systems can be written by using a matrix

$$A = \begin{pmatrix} 10 & -5 \\ 6 & -1 \end{pmatrix}$$

or simply as an adjacency matrix A

$$\frac{dy}{dt} = Ay. \tag{16}$$

By applying the discretization through the Laplace operator, the system of partial differential equations is formed to a system of ordinary differential equations:

$$\frac{\partial}{\partial t}\begin{pmatrix} u_1(t) \\ \vdots \\ u_{16}(t) \\ v_1(t) \\ \vdots \\ v_{16}(t) \end{pmatrix} = \begin{pmatrix} f(u_1(t), v_1(t)) \\ \vdots \\ f(u_{16}(t), v_{16}(t)) \\ g(u_1(t), v_1(t)) \\ \vdots \\ g(u_{16}(t), v_{16}(t)) \end{pmatrix} + \begin{pmatrix} \frac{D_u}{h^2}A & 0 \\ 0 & \frac{D_v}{h^2}A \end{pmatrix}\begin{pmatrix} u_1(t) \\ \vdots \\ u_{16}(t) \\ v_1(t) \\ \vdots \\ v_{16}(t) \end{pmatrix} \tag{17}$$

The constraint should be kept in mind that the application of spatial discretization allows an approximation of a solution for the nodes and not an exact solution.

Numerics of the differential equations

In the following

$$\frac{dx(t)}{dt} = f(t, x) \tag{18}$$

is a system of ordinary differential equations. By setting $x(t_0) = x_0$ the equation (18) presents an initial value problem. An autonomous differential equation system is obtained if the right side $f(x,t) = f(x)$ becomes independent of time. Numerical integration of ordinary differential equations can be performed by temporal discretization and an approximation of the temporal derivative by finite differences. In the following Δt is the distance between two nodes on a regular lattice of time points. By calculating the feed-forward difference the temporal derivative can be approximated by

$$\frac{dx(t)}{dt} \approx \frac{x(t+\Delta t) - x(t)}{\Delta t}.$$

Consequently, the explicit Euler-solver can be applied.

$$x(t+\Delta t) \approx x(t) + \Delta t \cdot f(x,t)$$

More generally an explicit single-step technique is presented by

$$x(t+\Delta t) \approx x(t) + \Delta t \cdot \Phi(x,t,\Delta t)$$

with the function Φ. If Φ contains dependencies of $x(t+\Delta t)$, then an implicit method is obtained.

Runge-Kutta method

In the Runge-Kutta method the function Φ has the form

$$\Phi = b_j \cdot K_j$$

whereas K_j presents the intermediate steps. For a s-step Runge-Kutta-technique and $j = 1..s$ we get:

$$K_j = f(t + c_j \Delta t, x + \Delta t \sum_{l=1}^{s} a_{jl} K_l)$$

The coefficients c_j, b_j and a_{jl} were taken from a Butcher-tableau (RK41) [1].

Embedded Runge-Kutta method

Within each step of time the step width Δt should be small enough in order to stay under a tolerance *tol* of precision. The error can be estimated by a procedure of orders p and q with $q < p$. x_1 is a step within the procedure of p-th order and $\widetilde{x_1}$ a step within the procedure of q-th order. Then the error can be estimated by

$$err = \frac{x_1 - \widetilde{x_1}}{\Delta t}$$

A step is accepted if the tolerance is larger than the error. In the other case the step width will be reduced. The *embedded* Runge-Kutta technique is step-controlled like the Dormand-Prince procedure which has been applied.

Gray-Scott model

The Gray-Scott model allows the description of a chemical reaction of the form

$$U + 2V \rightarrow 3V$$
$$V \rightarrow P$$

by using ordinary differential equations. The substance V can be considered as an activator and U is a substrate and does not operate as in inhibitor. By applying the mass action law, the following equations are obtained, where F is a constant term of production and $-Fu$ or $-kv - Fv$ are linear decay terms:

$$\frac{du}{dt} = -uv^2 + F - Fu$$
$$\frac{dv}{dt} = uv^2 - kv - Fv$$

As an example a quadratic domain $[0,1] \times [0,1]$ is considered. The solution of the PDEs has been realized as described in (14). The following parameters were used:

Tab 1. Parameter for the Gray-Scott model. The default parameters are the same as proposed by Buric [2].

Parameter	Value
F	0.05
k	0.02
D_A	0.001
D_S	0.2
dt	0.01

For a step of time $t = 10000$ the pattern as shown in Fig P has been developed. Obviously, it has a completely other distribution of concentrations than in the homogenous equilibrium of $u = (1, ..., 1), v = (0, ..., 0)$. Therefore, it constitutes a Turing-instability.

Gierer-Meinhardt model

The Gierer-Meinhardt model describes the diffusive interaction of two substances. In the following the two-dimensional case is considered. The concentrations of the two substances are given by $U(x, y, t)$ and $V(x, y, t)$ at point or node (x, y) within time point t. The change of the concentrations within time is described by the two PDEs

$$\frac{dU(t)}{dt} = \underbrace{r_u \cdot \frac{U^2}{(1 + \kappa U^2)V} - \mu_u \cdot U + \sigma_u}_{=:f(U,V)} \qquad + D_u \Delta U$$
$$\frac{dV(t)}{dt} = \underbrace{r_v \cdot U^2 - \mu_v \cdot V + \sigma_v}_{=:g(U,V)} \qquad + D_v \Delta V$$

The Laplace-operator Δ

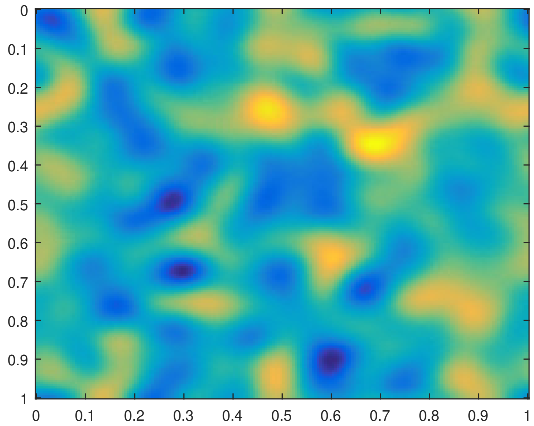

Fig P. Gray-Scott model in a regular lattice. Distribution of concentrations of U in a regular grid after 100000 iterations.

$$\Delta U = \frac{\partial^2 U}{\partial x^2} + \frac{\partial^2 U}{\partial y^2}, \quad \Delta V = \frac{\partial^2 V}{\partial x^2} + \frac{\partial^2 V}{\partial y^2}$$

provides the diffusion or spatial spread based on Brownian motion of the two substances. The model depends on 9 parameters, whereas the parameters $r_u, r_v, \mu_u, \mu_v, \kappa, \sigma_u$ and σ_v determine the local reaction of the two substances and the parameters D_u and D_v are the velocity of spatial spreading of the two substances. The reaction constants of the activator r_u and inhibitor r_v have default values of 0.01 and 0.02. The decay rates of activator μ_u and inhibitor μ_v have default values of 0.02 and 0.01 the decay rates for the activator (u) and inhibitor (v). For controlling the level of saturation or limitation of the autocatalysis of the activator κ is used with a default value of 0.025. The constant production rate of the activator is σ_u and the constant production rate of the inhibitor is σ_v. Firstly, in analogy to the Gray-Scott model the outcome of RD is considered. By using the parameters

Tab 2. Parameters of the local reaction. The parameters are based on Koch and Meinhardt [3, 4].

Parameter	Value
μ_u	0.01
μ_v	0.02
r_u	0.01
r_v	0.02
σ_u	0
σ_v	0
κ	0.025

and the diffusion constants
a Turing-pattern can be generated (Fig Q).

Tab 3. Constants of the diffusion.

Parameter	Value
D_u	0.01
D_v	0.02

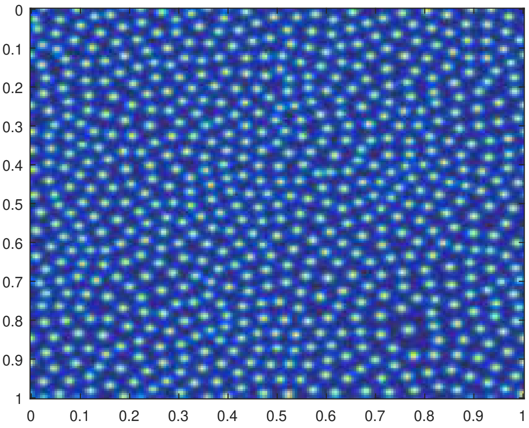

Fig Q. Gierer-Meinhardt model in a regular lattice. Distribution of concentrations of the substance U in the equilibrium.

Mimura-Murray model

The prey-predator model of Lotka and Volterra [5] uses two population sizes of preys $P(t)$ and predators $Q(t)$ where P is the predator and Q the prey population. A generalized model has been introduced by [6, 7]. The parameters a, b, c characterize the prey population and parameter d belong to the predator population. Here, the two populations are considered on a domain with two spatial dimensions in such a way that the PDEs have the form [8]:

$$\frac{\partial P(x,y,t)}{\partial t} = \left(\frac{a + bP - P^2}{c} - v\right) P + D_P \Delta P \tag{19}$$

$$\frac{\partial Q(x,y,t)}{\partial t} = \left(Q - (1 - dQ)\right) v + D_Q \Delta Q \tag{20}$$

The following parameters turned out to produce an equilibrium state in a regular lattice

Tab 4. Parameters of the local reaction of the Mimura-Murray model were used as suggested by Asllani et al. [9].

Parameter	Value
a	35
b	16
c	9
d	0.4
D_u	0.1
D_v	0.01

Reaction-diffusion models in networks

Networks are discrete structures and the Laplace operator was written in the form of a matrix after approximation of the second derivative by finite differences. This matrix corresponds on the non-diagonal elements of the negative adjacency matrix of the regular lattice. In the following, the Laplace operator is modified for the application of diffusion in networks. The Laplace matrix of a non-directed graph $G = (V, E)$ is defined as

$$L_{ij} = \begin{cases} -d_i & i = j \\ 1 & \{i, j\} \in E \\ 0 & \text{otherwise} \end{cases}$$

whereas d_i is the degree of node i. For a directed graph [10, 11] the following possibilities should be differentiated:

- $L_1 =: A^T - D_{out}$
- $L_2 =: A^T - D_{in}$
- $L_3 =: A - D_{out}$
- $L_4 =: A - D_{in}$

These variants have been implemented in *neuroVIISAS*. The difference between L_1 and L_2 is demonstrated in an empirical network which was used for the following experiments as well (Fig R). To allow a comparison with L_1 and L_3 of output diffusion, the equilibrium state of a Turing dot pattern is shown in Fig AT for L_1 and Fig AV for L_3.

When performing a diffusion process without reaction then a single Euler step with standardized step width and neglection of the diffusion constant is:

$$U_{t+\Delta t} = U_t + L \cdot U_t$$

Weight modulation within diffusion processes

Neurodegenerative disease in particular Multiple Sclerosis leads to time-dependent progressive patterns of functional loss. This functional loss is shaped by changing connectivity weights within the run time of a reaction-diffusion process. That means that the coupling matrix L is time dependent and the reaction-diffusion with time-dependent coupling is:

$$\frac{dU}{dt} = f(U) + L(t) \cdot U$$

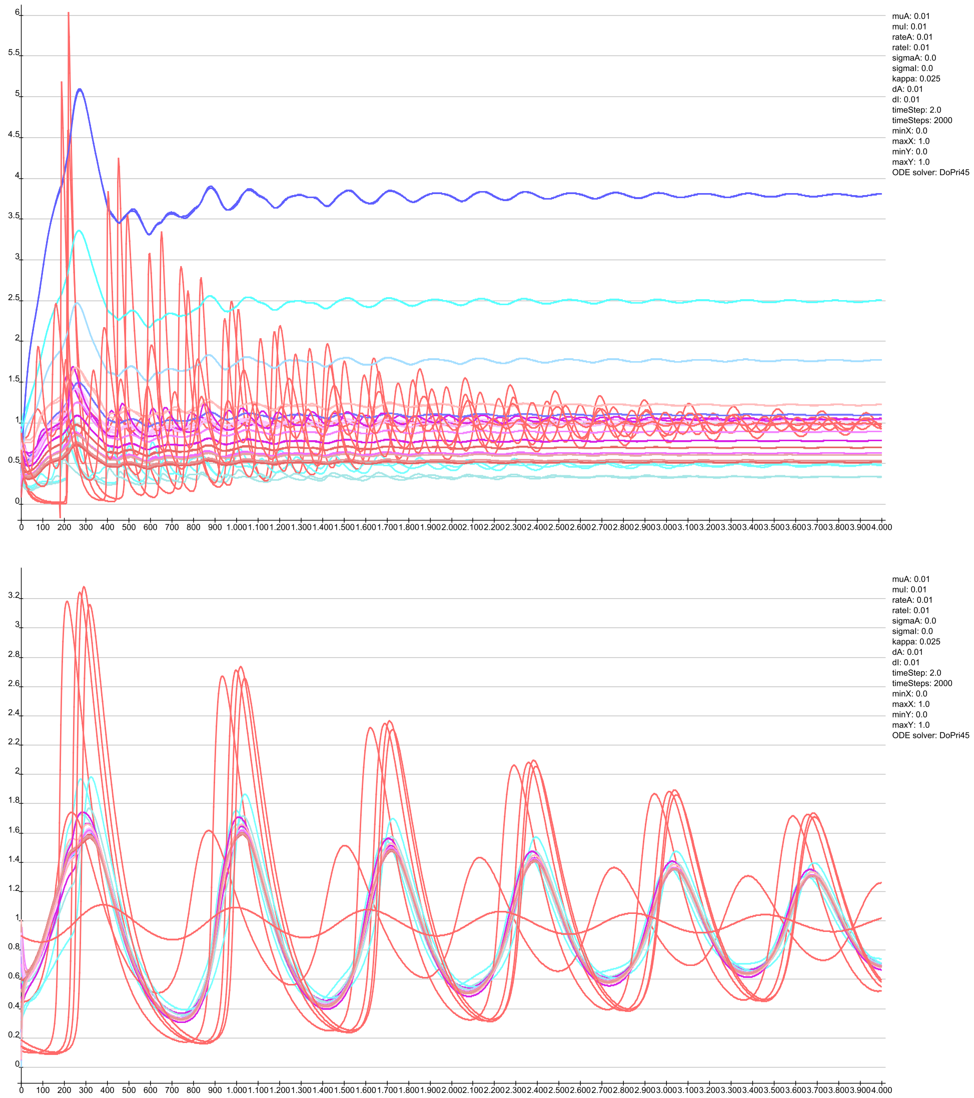

Fig R. GM RD through either output or input connections. Top: GM RD result by using the output connections of the transposed adjacency matrix (L_1). Bottom: GM RD result by using the input connections of the transposed adjacency matrix (L_2).

The weight modulation (Fig S) has been realized for subsets of nodes of a network in order to specify *diseased nodes* and their input or output connections.

To illustrate the change of connectivity weights a small cyclic 4 node graph (Fig T) should be considered. The node 2 is selected for weight reduction of outgoing or efferent connections.

The corresponding adjacency matrix has the form

$$A = \begin{pmatrix} 0 & e_1 & 0 & 0 \\ 0 & 0 & e_2 & e_3 \\ 0 & 0 & 0 & e_4 \\ e_5 & 0 & 0 & 0 \end{pmatrix}$$

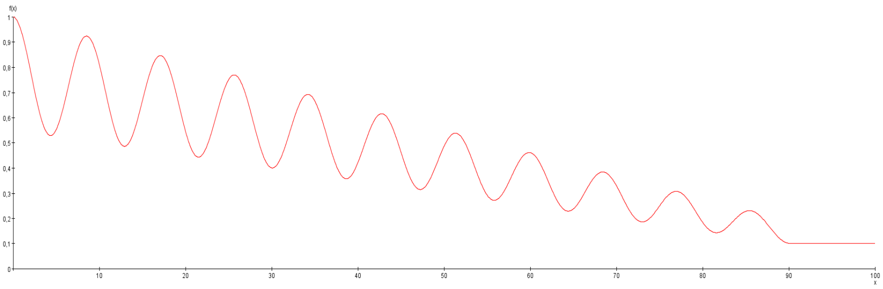

Fig S. Weight modulation. Example of a time-dependent change of connection weights.

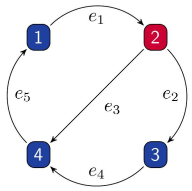

Fig T. Example graph. This graph with 4 nodes is used as an example for showing how weight modulation affects the coupling matrix.

For determining the coupling matrices L_1, L_2, L_3 and L_4 matrices A^T, D_{in} and D_{out} are needed.

$$A^T = \begin{pmatrix} 0 & 0 & 0 & e_5 \\ e_1 & 0 & 0 & 0 \\ 0 & e_2 & 0 & 0 \\ 0 & e_3 & e_4 & 0 \end{pmatrix} \quad D_{in} = \begin{pmatrix} e_5 & 0 & 0 & 0 \\ 0 & e_1 & 0 & 0 \\ 0 & 0 & e_2 & 0 \\ 0 & 0 & 0 & e_3 + e_4 \end{pmatrix}$$

$$D_{out} = \begin{pmatrix} e_1 & 0 & 0 & 0 \\ 0 & e_2 + e_3 & 0 & 0 \\ 0 & 0 & e_4 & 0 \\ 0 & 0 & 0 & e_5 \end{pmatrix}$$

The time dependencies of the weights of the outgoing connection of node 2 is expressed by the matrix

$$M = \begin{pmatrix} 1 & 0 & 0 & 0 \\ 0 & f(t) & 0 & 0 \\ 0 & 0 & 1 & 0 \\ 0 & 0 & 0 & 1 \end{pmatrix}$$

This can be generalized for any network with n nodes and a given selection of nodes or regions with the set of indices $I \subset \{1, 2, ..., n\}$ of selected nodes. Then the time dependency can be expressed with the matrix $M(t)$

$$M_{ii} = \begin{cases} 1, & \text{falls } i \notin I \\ f(t), & \text{falls } i \in I \end{cases}$$

For the coupling matrix $L_1 = A^T - D_{out}$ and $L_3 = A - D_{out}$ a simple representation of time dependency is given:

$$L_1(t) = (A^T - D_{out}) \cdot M(t)$$
$$L_3(t) = M(t) \cdot A - D_{out} \cdot M(t)$$

By analogy reduction of connection weights can be defined for ingoing connections as well as ingoing and outgoing connections.

Additive noise

In order to assess the stability of the reactions-diffusion systems a noise term has been added.

Adding internode Euclidean distances to the diffusion

We have described how weighted connections are incorporated into the diffusion process. Furthermore, the distance between regions as the Euclidean distance between gravitation centers of the regions which are characterized by contours in the digitized stereotaxic atlas is regarded in the diffusion term. Large connections weights transfer concentrations by diffusion strongly. Long distances between nodes delay the transmission by reducing the diffusion velocity of matter. Hence, the coupling should be influenced by distance, only. This can be realized by applying delay differential equations (DDE) [12–17] to the preceding concentration. Therefore, it is necessary to initiate the systems with more initial conditions because for each node an initial interval with the length of the delay must be provided.

An alternative has been chosen here. Each edge is subdivided by inserted nodes. In the most simple case, a graph with two nodes and one connection is divided by one inserted node (Fig U).

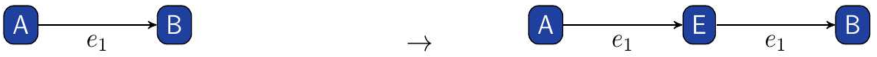

Fig U. Concept of node insertion. Left: Original graph. Right: Graph with added inserted node E.

The differential equation for modeling the example graph (Fig U) is given by using the diffusion constant D:

$$\frac{dA}{dt} = -D \cdot e_1 \cdot A$$
$$\frac{dB}{dt} = D \cdot e_1 \cdot A$$

The system of differential equations of the graph with one inserted node is:

$$\frac{dA}{dt} = -D \cdot e_1 \cdot A$$
$$\frac{dE}{dt} = D \cdot e_1 \cdot A - \Theta \cdot D \cdot e_1 \cdot E$$
$$\frac{dB}{dt} = \Theta \cdot D \cdot e_1 \cdot E$$

The parameter Θ controls the leverage or the extension of delay of the inserted node. The effect is shown by applying the initial values $(A_0, B_0) = (1, 0)$ and

$(A_0, E_0, B_0) = (1, 0, 0)$ for the inserted node, respectively. The diffusion constants are $D = 1$ and $e_1 = 1$ in both cases. In Fig V small Θ lead to a slow increase of concentration in node B because the node E interacts as an inserted node. For a large Θ, the inserted cache is depleted faster. Therefore, with increasing Θ the effect of inserted nodes decreases. In the limit case $\Theta \to \infty$ the effect of inserted node generated the same progress of concentration for A and B like in the case without inserted node.

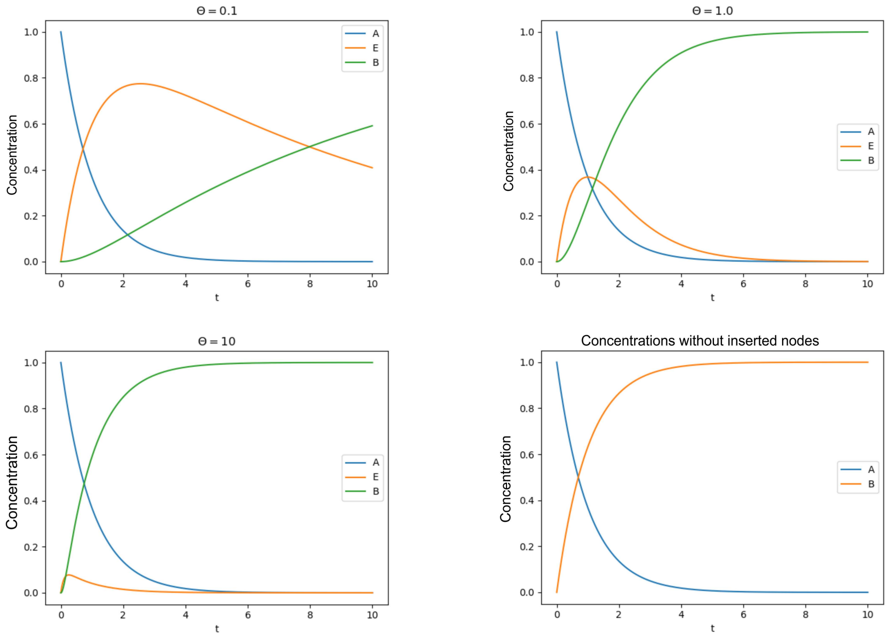

Fig V. Progression of changes of concentrations at the nodes for different Θ. The functions of concentrations are shown for different Θ and inserted nodes and for the case without node insertion where regions A and B occur, only.

Especially for digraphs, reciprocal edges should be treated independently. If the edge between 2 nodes is reciprocal then an inserted node should be generated for each direction. For a larger example (Figs. Fig AB, Fig AC) the procedure is described in the supplement.

References

1. Butcher JC. Numerical Methods for Ordinary Differential Equations. Wiley, 2016.

2. Buric F. Pattern formation and chemical evolution in extended Gray-Scott models. Master of Science thesis in Complex Adaptive Systems, Division of Physical Resource Theory, Department of Energy and Environment, Chalmers University of Technology, Gothenburg, Sweden, 2014.

3. Koch AJ, Meinhardt H. Biological pattern formation: from basic mechanisms to complex strucutres. Rev Mod Phys. 1994; 66: 1481-1507.

4. Gierer A, Meinhardt H. A theory of biological pattern formation. Kybernetik. 1972; 12: 30-39.

5. Lotka AJ. Elements of Physical Biology. Williams and Wilkins, Baltimore, 1925.

6. Mimura M, Murray JD. On a diffusive prey-predator model which exhibits patchiness. J Theor Biol. 1978; 75: 249-262.

7. Murray JD. Mathematical Biology, I. An Introduction, third edition. Interdiscip Appl Math. 2002; Vol. 17, Springer-Verlag, New York.

8. Mimar S, Juane MM, Park J, Muñuzuri AP, Ghoshal G. Turing patterns mediated by network topology in homogeneous active systems. Phys Rev E. 2019; 99: 062303.

9. Asllani M, Busiello DM, Carletti T, Fanelli D, Planchon G. Turing instabilities on Cartesian product networks. Sci Rep. 2015; 5: 12927.

10. Cencetti G, Clusella P, Fanelli D. Pattern invariance for reaction-diffusion systems on complex networks. Sci Rep. 2018; 8: 16226.

11. Nakao H, Mikhailov AS. Turing patterns in network-organized activator-inhibitor systems. Nat Phys. 2010; 6: 544-550.

12. Rombouts J, Gelens L, Erneux T. Travelling fronts in time-delayed reaction-diffusion systems. Philos Trans A Math Phys Eng Sci. 2019; 377: 20180127.

13. Hale JK. Theory of Functional Differential Equations. Springer-Verlag, 1977.

14. Bellen A, Zennaro M. Numerical methods for delay differential equations, numerical mathematics and scientific computation. The Clarendon Press, Oxford University Press, New York, 2003.

15. Kuang Y. Delay differential equations with applications in population dynamics. Academic Press, Boston, 1993.

16. Hoppensteadt FC. An introduction to the mathematics of neurons. Modeling in the frequency domain. Cambridge University Press, Cambridge, 1997.

17. Gourley SA, Kuang Y. A delay reaction-diffusion model of the spread of bacteriophage infection. SIAM J Appl Math. 2005; 65: 550-566.

Stochastic noise

A stochastic noise model was used to investigate the stability of the GM reactions-diffusion system. The coupled Ornstein-Uhlenbeck process is compared with additive normal distributed noise.

Coupled Ornstein-Uhlenbeck processes in networks

In the following, coupled Ornstein-Uhlenbeck processes in networks are considered in combination with a diffusive coupling. For a diffusion without reaction term the step $U_t \to U_{t+\Delta^t}$ is given by

$$U(t+dt) = U(t) + \Delta t \cdot \delta \cdot L \cdot U(t)$$

whereas L is the Laplace- or the general coupling matrix of the network. The Euler-Maruyama procedure for the start vector U_0 can be written as:

$$U(t=0) = U_0$$
$$U(t+\Delta t) = U(t) + \Delta t \cdot L \cdot U(t) + \Delta t \cdot (\Theta(\mu - U(t))) + \sigma\sqrt{\Delta t}\mathcal{N}(0,1)$$

OU-processes with diffusive coupling

The parameters $\sigma = \Theta = 1$, $D_u = 0.1$ and $\mu = 10$ were defined. As an initial vector random values between 0 and 1 were used. The resulting progression of concentrations is shown in Fig W. For a better overview, the progression of concentration is displayed for a subset of nodes, only.

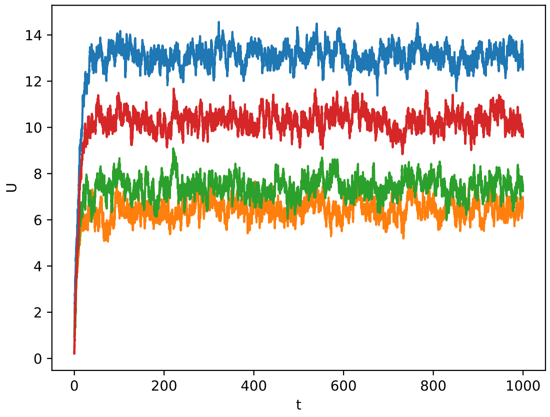

Fig W. OU-processes. Stand alone OU-processes with $D_u = 0$ and $\Theta = \sigma = 1$ as well as $\mu = 10$.

Gierer-Meinhardt with OU-process as a term of noise

First of all, the reaction term of the Gierer-Meinhardt reaction-diffusion systems with adding the noise of an OU-process is considered. To this end the stochastic differential equation system can be considered:

$$\frac{dU}{dt} = f(U,V)$$
$$\frac{dV}{dt} = g(U,V)$$
$$dR = \Theta(U-R)dt + \sigma dW(t)$$

In the case of $\sigma = 0$, the Euler-Maruyama procedure can be simplified to an Euler-procedure with constant increment. The difference of the components U and R is interesting because R presents the following noise version U. The equation of R is built so that R pursues asymptotically against $U(t)$. Subsequently, a delay between U and R appears, which is shown in the following example.

The example function has the form of

$$U = sin(t)$$
$$\frac{dR}{dt} = \Theta(U-R)$$

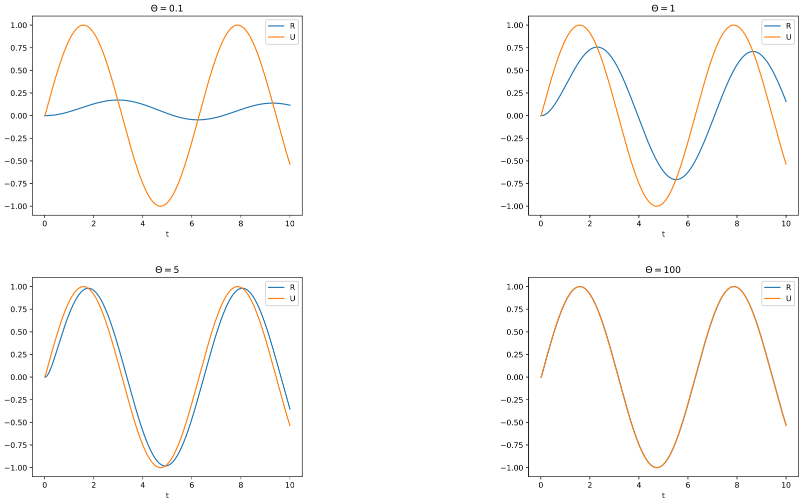

Fig X. Variation of Θ. Difference between U and R for different Θ and same initial conditions $U_0 = R_0 = 0$.

In Fig X the progression of R for different Θ is shown. In all cases the tendencies to the value of U are recognizable, however, for small values of Θ large differences are obvious. Intuitively accessible Θ represents a measure for the change of R. If the change of $\frac{dU}{dt}$ is too large, then it is not possible anymore that with a small Θ an asymptotic convergence of R against U can be reached. In this case the progression of the function of R depends of U. Thus, the parameter Θ can be interpreted as a delay. If Θ becomes sufficiently large, then the derivation of R to U becomes marginal.

Gierer-Meinhardt and Ornstein-Uhlenbeck with $\sigma \neq 0$

In the following Θ should be large enough so that no significant delay between R and U can be expected. The following system is considered

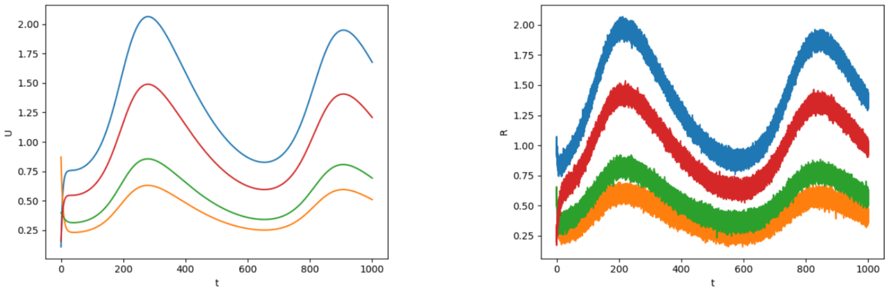

Fig Y. Comparison of a GM-model without noise and with OU noise. Progression of concentrations without noise and with noise.

$$\frac{dU}{dt} = f(U,V) + L \cdot U$$
$$\frac{dV}{dt} = g(U,V) + L \cdot V$$
$$dR_U = \Theta(U - R_U)dt + \sigma dW(t)$$
$$dR_V = \Theta(U - R_V)dt + \sigma dW(t)$$

The progression of concentrations of U and V is not influenced by the term of noise. The influence of noise is given exclusively by R. As shown in Fig Z, the results do not deviate in terms of quality from noise added afterward if the Θ is sufficiently large. Quantitatively a stronger effect of the term of noise to the progression of the function for the same σ is obvious.

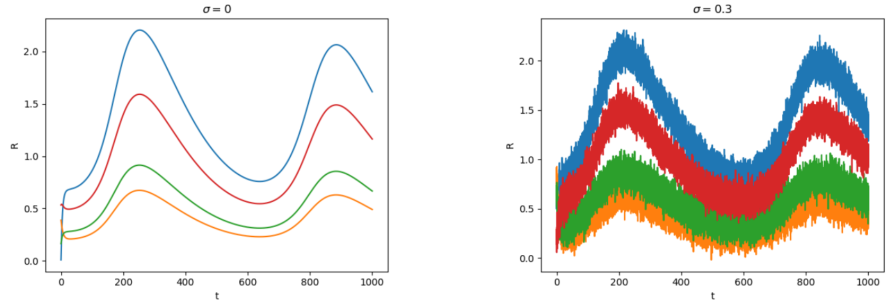

Fig Z. Effects of noise. Progression of R for sufficiently large Θ.

Implementation of noise in $neuroVISAAS$

In $neuroVISAAS$ the following form of noise term has been implemented

$$dU = (f(U,V) + L \cdot U)dt + \sigma dW(t)$$
$$dV = (g(U,V) + L \cdot V)dt + \sigma dW(t)$$

In addition to the terms of reaction and diffusion, a further additive term of noise has been built in. Here, an effect on the progression of functions of U and V was found. Different σ are shown in Fig AA.

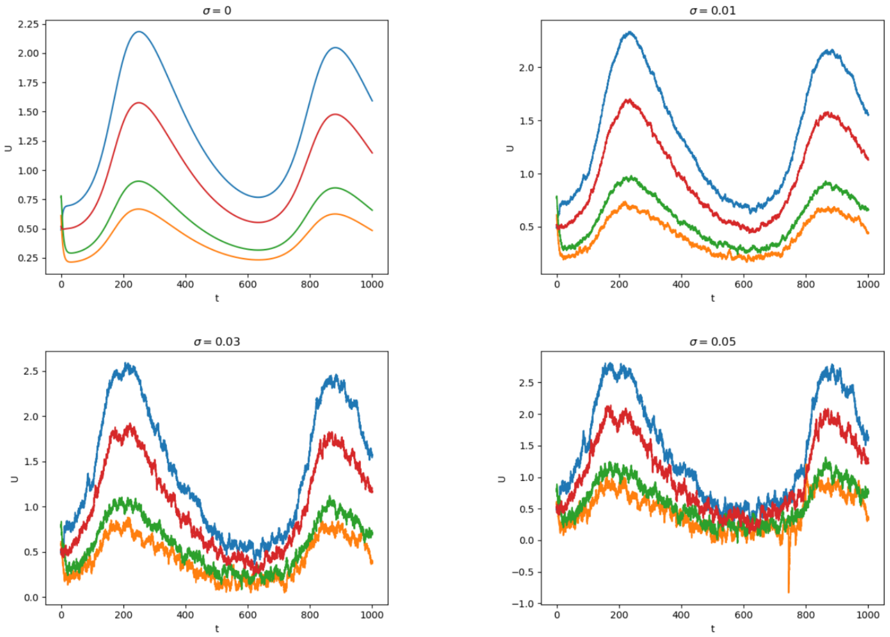

Fig AA. Variation of σ. Progression of functions R for different σ.

Distances in RD processes

The concept of using internode Euclidean distances is represented for a graph of 4 nodes and 5 edges.

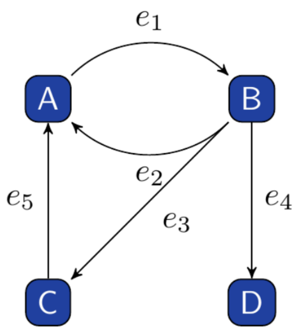

Fig AB. Example graph with a reciprocal edge. The reciprocal edge is defined by e_1 and e_2. A cycle from B to C and from C to A and from A back to B as well as an isolated node D which has no output covers some basic connectional features.

With regard to the graph with 2 nodes (Fig U) the differential equation systems for modeling the diffusion by using the Laplace matrix L_3 and the diffusion constant D for the larger example with a reciprocal edge (Fig AC) is given by:

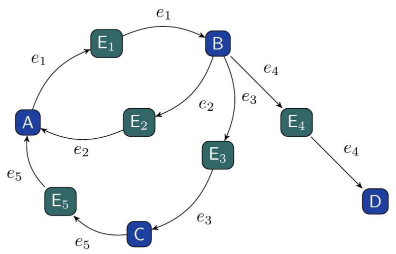

Fig AC. Example graph with inserted nodes. The inserted nodes are indicated by E_i. 4 primary nodes are connected by 5 connections and 5 inserted nodes.

$$\frac{dA}{dt} = -D \cdot e_1 \cdot A + D \cdot e_2 \cdot B + D \cdot e_5 \cdot C$$
$$\frac{dB}{dt} = D \cdot e_1 \cdot A - D \cdot (e_2 + e_3 + e_4) \cdot B$$
$$\frac{dC}{dt} = D \cdot e_3 \cdot B - D \cdot e_5 \cdot C$$
$$\frac{dD}{dt} = D \cdot e_4 \cdot B$$

This relates to the matrix notation as follows:

$$\frac{d}{dt}\begin{pmatrix} A \\ B \\ C \\ D \end{pmatrix} = D \cdot \begin{pmatrix} -e_1 & e_2 & 0 & e_5 \\ e_1 & -e_2 - e_3 - e_4 & 0 & 0 \\ 0 & e_3 & -e_5 & 0 \\ 0 & e_4 & 0 & 0 \end{pmatrix} \begin{pmatrix} A \\ B \\ C \\ D \end{pmatrix}$$

The differential equation system of the graph with inserted nodes (Fig AC) has the form of:

$$\frac{dA}{dt} = -D \cdot e_1 \cdot A + \Theta_2 \cdot D \cdot e_2 \cdot E_2 + \Theta_5 \cdot D \cdot e_5 \cdot E_5$$
$$\frac{dB}{dt} = \Theta_1 \cdot D \cdot e_1 \cdot E_1 - D \cdot (e_2 + e_3 + e_4) \cdot B$$
$$\frac{dC}{dt} = -D \cdot e_5 \cdot C + \Theta_3 \cdot D \cdot e_3 \cdot E_3$$
$$\frac{dD}{dt} = \Theta_4 \cdot D \cdot e_4 \cdot E_4$$
$$\frac{dE_1}{dt} = D \cdot e_1 \cdot A - \Theta_1 \cdot D \cdot e_1 \cdot E_1$$
$$\frac{dE_2}{dt} = D \cdot e_2 \cdot B - \Theta_2 \cdot D \cdot e_2 \cdot E_2$$
$$\frac{dE_3}{dt} = D \cdot e_3 \cdot B - \Theta_3 \cdot D \cdot e_3 \cdot E_3$$
$$\frac{dE_4}{dt} = D \cdot e_4 \cdot B - \Theta_4 \cdot D \cdot e_4 \cdot E_4$$
$$\frac{dE_5}{dt} = D \cdot e_5 \cdot C - \Theta_5 \cdot D \cdot e_5 \cdot E_5$$

This system can be simplified with regard to matrix notation by using the incidence matrix for the source graph (Fig AB). The edges are indicated by

$K_1 = (A, B), K_2 = (B, A), K_3 = (B, C), K_4 = (B, D)$ and $K_5 = (C, A)$ then the incidence matrix has the following form:

$$I = \begin{pmatrix} e_1 & -e_2 & 0 & 0 & -e_5 \\ -e_1 & e_2 & e_3 & e_4 & 0 \\ 0 & 0 & -e_3 & 0 & e_5 \\ 0 & 0 & 0 & -e_4 & 0 \end{pmatrix}$$

The matrix can be subdivided with regard to its positive portion

$$I_p = \begin{pmatrix} e_1 & 0 & 0 & 0 & 0 \\ 0 & e_2 & e_3 & e_4 & 0 \\ 0 & 0 & 0 & 0 & e_5 \\ 0 & 0 & 0 & 0 & 0 \end{pmatrix}$$

and its negative portion

$$I_n = \begin{pmatrix} 0 & -e_2 & 0 & 0 & -e_5 \\ -e_1 & 0 & 0 & 0 & 0 \\ 0 & 0 & -e_3 & 0 & 0 \\ 0 & 0 & 0 & -e_4 & 0 \end{pmatrix}$$

The adjacency matrix of the new graph with inserted nodes (Fig AC) has the form of:

$$A' = \begin{pmatrix} 0 & I_p \\ -I_n^T & 0 \end{pmatrix}$$

With the related Laplace-matrix L_3 the differential equation system describing the diffusion can be written as follows:

$$\frac{d}{dt} \begin{pmatrix} A \\ B \\ C \\ D \\ E_1 \\ \vdots \\ E_5 \end{pmatrix} = D \cdot L_3 \begin{pmatrix} A \\ B \\ C \\ D \\ E_1 \\ \vdots \\ E_5 \end{pmatrix}$$

Formalization of a RD with regard of distances

In the formalization of the RD with delay or attenuation of the diffusion term it should be emphasized that the reaction term is applied to the source node, only. Proceeding from a RD system with the adjacency matrix A, reaction term f, Laplace matrix L_3 and vector of concentration Y as a differential equation system we get

$$\frac{dY}{dt} = D \cdot L_3 \cdot Y + f(Y)$$

and are able to determine the adjacency matrix A^{neu} and Laplace-matrix L_3^{neu} of the new network with inserted nodes. As far as the source network contains n nodes and m edges the differential equation system of the extended network has the form:

$$\frac{d}{dt}\begin{pmatrix} Y_1 \\ \vdots \\ Y_n \\ E_1 \\ \vdots \\ E_m \end{pmatrix} = D \cdot L_3 \begin{pmatrix} Y_1 \\ \vdots \\ Y_n \\ E_1 \\ \vdots \\ E_m \end{pmatrix} + \begin{pmatrix} f_1(Y) \\ \vdots \\ f_n(Y) \\ 0 \\ \vdots \\ 0 \end{pmatrix}$$

For the implementation of larger networks, the Laplace matrix for the extended network has the dimension $(n+m) \times (n+m)$. By taking into account that newly inserted nodes possess only one input and one output, the Laplace matrix is sparse and computing with vectors is faster.

Supplement: Supporting material

In this section of the supplement, additional explanations and further material are given for the implementation and intermediate results of the RD systems that have been studied. In the last part, we have compiled the supplementary figures to the results section of the article.

The Butcher-Tableau for the Dormand-Prince method.

$$
\begin{array}{c|ccccccc}
0 & & & & & & & \\
\frac{1}{5} & \frac{1}{5} & & & & & & \\
\frac{3}{10} & \frac{3}{40} & \frac{9}{40} & & & & & \\
\frac{4}{5} & \frac{44}{45} & -\frac{56}{15} & \frac{32}{9} & & & & \\
\frac{8}{9} & \frac{19372}{6561} & -\frac{25360}{2187} & \frac{64448}{6561} & -\frac{212}{729} & & & \\
1 & \frac{9017}{3168} & -\frac{-355}{33} & \frac{46732}{5247} & \frac{49}{176} & -\frac{5103}{18656} & & \\
1 & \frac{35}{384} & 0 & \frac{500}{1113} & \frac{125}{192} & -\frac{2187}{6784} & \frac{11}{84} & \\
\hline
 & \frac{35}{384} & 0 & \frac{500}{1113} & \frac{125}{192} & -\frac{2187}{6784} & \frac{11}{84} & 0 \\
 & \frac{5179}{57600} & 0 & \frac{7571}{16695} & \frac{393}{640} & -\frac{92097}{339200} & \frac{187}{2100} & 0
\end{array}
$$

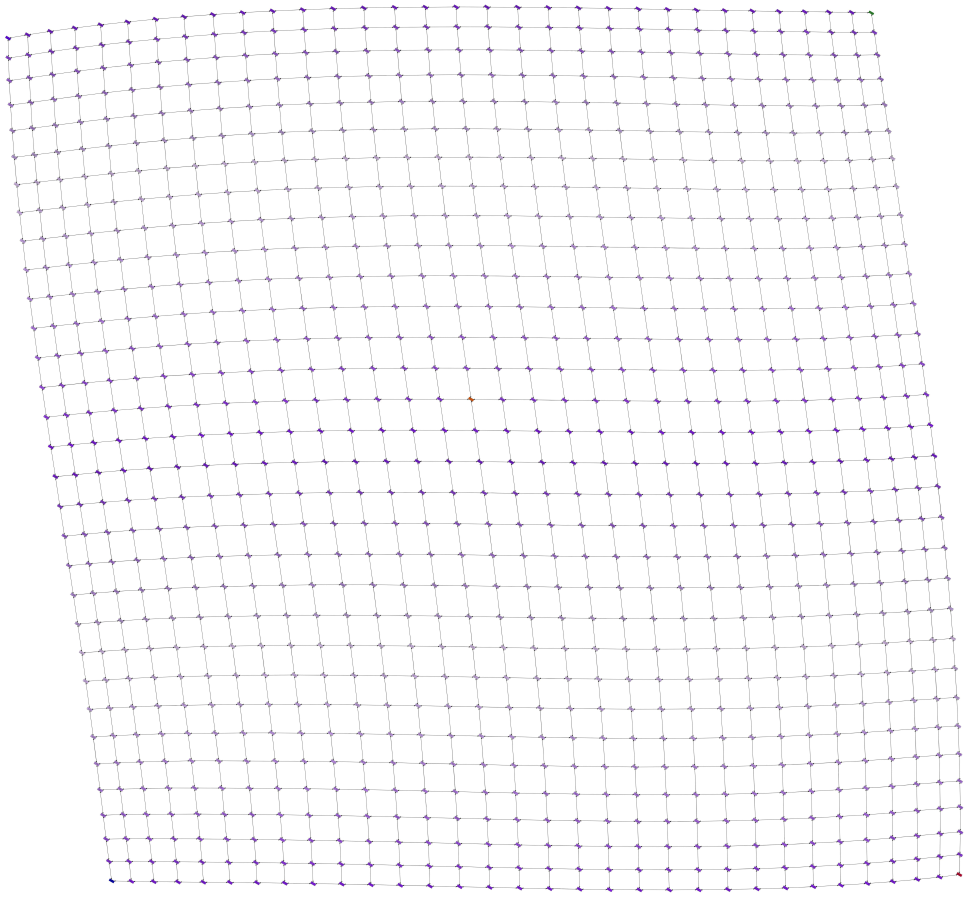

Fig AD. An example of a 32×32 lattice. The 32×32 lattice with 4 connected neighbors which was used to generate the Turing pattern. A center node (530), and corner nodes (1, 32, 993 1024) are labeled by different colors which were used in Figs Fig AT and Fig AU.

Tutorial part 3: RD systems in node chains and circuits

The RD systems of Gray-Scott (GS), Gierer-Meinhardt (GM) and Mimura-Murray (MM) show different progressions of their functions in directed networks with connections weights as well as without connection weights. The results of the RD systems under different initial conditions and constraints with regard to non-weighted, weighted, weight changes and inter-node distances in node-chains, node-circuits and a subconnectome will be presented in the following.

Unique initial conditions

All RD systems were applied to a 6 node chain (Fig AE). The color code of the nodes were applied to the graphs in the diagrams (Figs. Fig AF-Fig AH). The termination of RD processes were done until multiple detectable peaks of oscillating functions were recognizable. For example, we found this pattern in the GS process after 10000, in the GM process after 5000 and in the MM process after 5000 steps. However, the step sizes in the Runge-Kutta45 solver are adapted dynamically. The GS functions of the 6 nodes show only a few waves which reach a stable equilibrium very fast (Fig AF). The GM function follows a damped oscillation (Fig AG) whereas the phases stay relatively constant. The MM functions display sharp oscillating curves without damping (Fig AH). Peaks of the MM functions curves seem to follow a low-frequency oscillatory process.

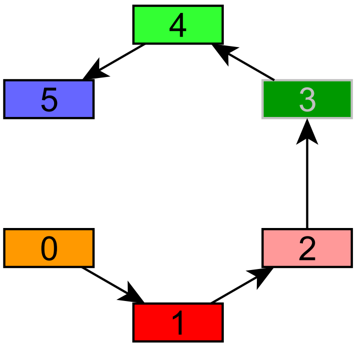

Fig AE. Test graph. The test graph has a directed node-chain structure.

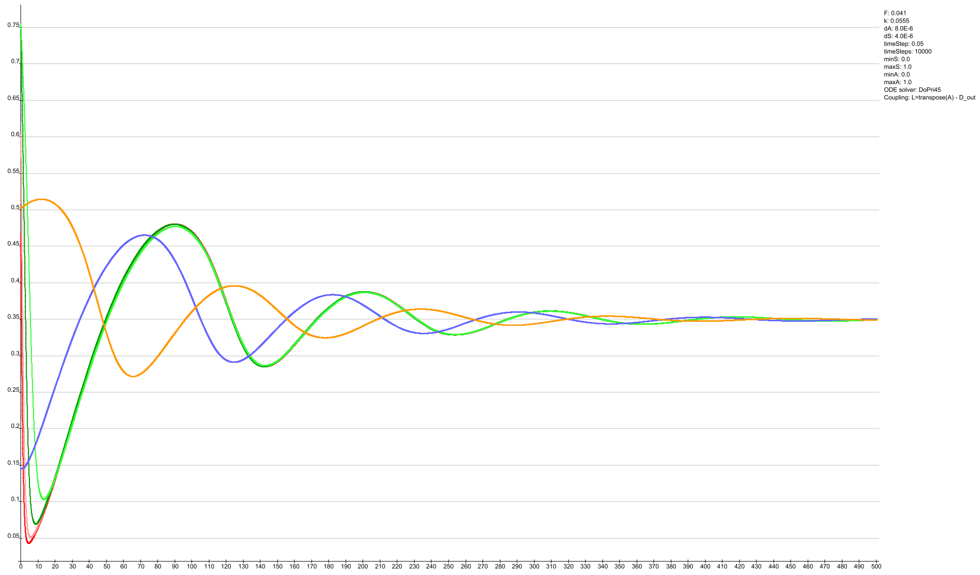

Fig AF. Functions of concentrations of the GS RD model. The GS function graph after 500 iterations for the regions arranged in a chain (Fig AE).

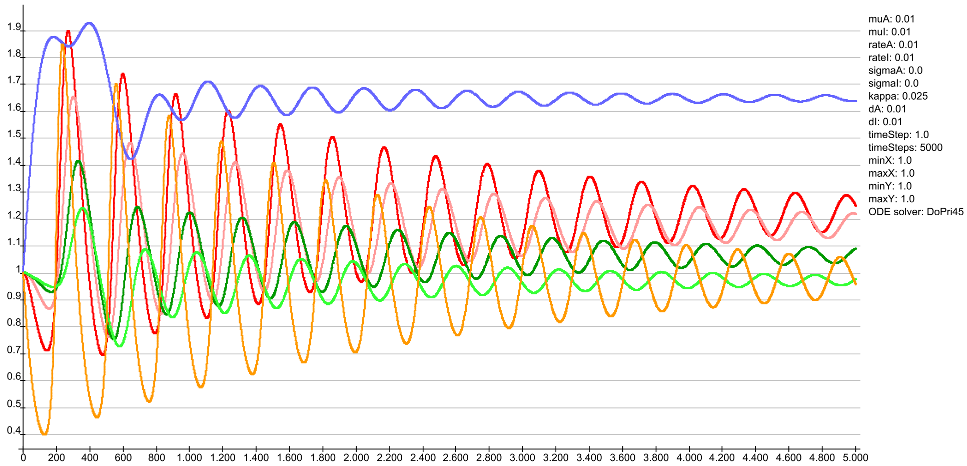

Fig AG. Functions of concentrations of the GM RD model. The GM function graphs after 5000 iterations for the regions arranged in a chain (Fig AE).

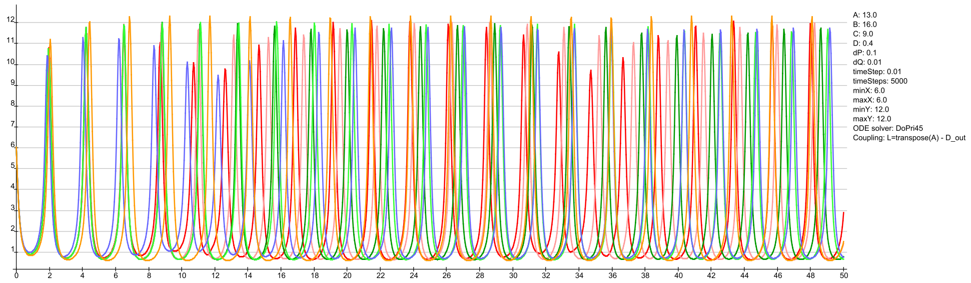

Fig AH. Functions of concentrations of the MM RD model. MM function graph after $t = 50$ iterations for the regions arranged in a chain (Fig AE).

Initial conditions for selected nodes

When applying initial conditions ($min_S = 1$, $max_S = 1$, $min_A = 1$, $max_A = 1$) to node 0 in the GS model all functions of the nodes have the same progression. A typical first wide wave with the largest peak is generated followed by a strong damping and 2 succeeding peaks (Fig AI). By assigning the initial condition to node 0 in the GM model ($V_0 = 1$, $W_0 = 1$) the oscillations as observed in (Fig AG) are damped. The functions show a relatively constant progression with similar phases. The function of node 6 has the largest initial peak and the strongest damping with small amplitudes (Fig AJ). The GM model appears to be more sensitive to an initial condition for a particular node than the GS model. After setting the initial values of the MM model to $min_X = 1$, $max_X = 1$, $min_Y = 1$ and $max_Y = 1$ the oscillation of all nodes (Fig AK) are comparable like in Fig Fig AH. The nodes 1 to 5 which have initial values $min_X = 0$, $max_X = 0$, $min_Y = 0$ and $max_Y = 0$ generate larger initial peaks than node 0 (Fig AG). Because the GS model does not process prolonged oscillations in a network it was not investigated under the following conditions and networks (Figs. Fig AF, Fig AI).

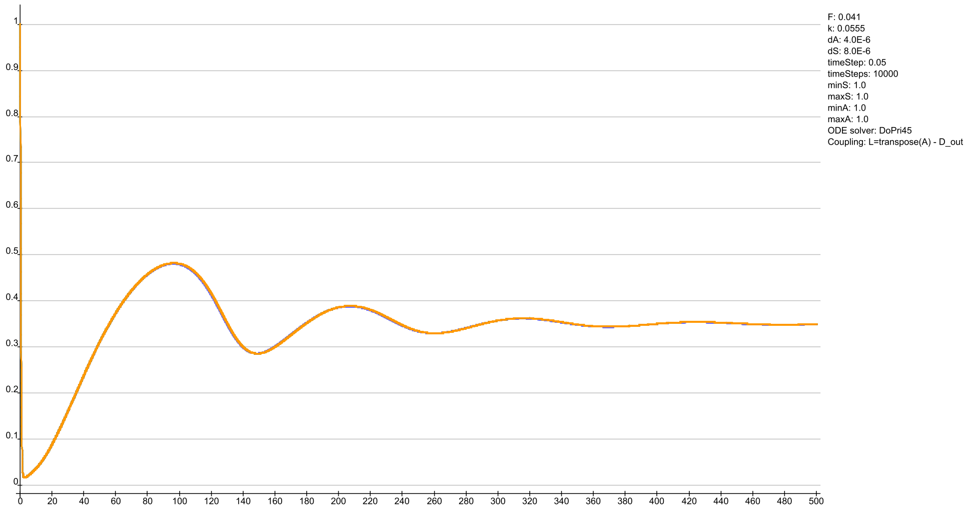

Fig AI. Functions of concentrations of the GS RD model with constant initial conditions. The GS function graph after 500 iterations. The initial conditions for node 0 were set to $min_S := 1$, $max_S = 1$, $min_A = 1$ and $max_A = 1$. The function graphs of all nodes are very similar and overlap.

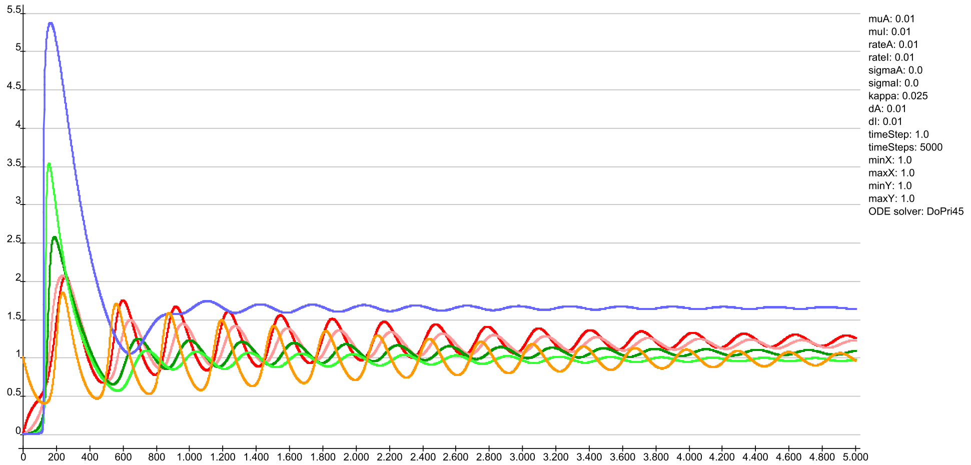

Fig AJ. Functions of concentrations of the GM RD model with constant initial conditions. The GM function graphs after 5000 iterations with initial conditions for node 0 of $V_0 = 1$ and $W_0 = 1$.

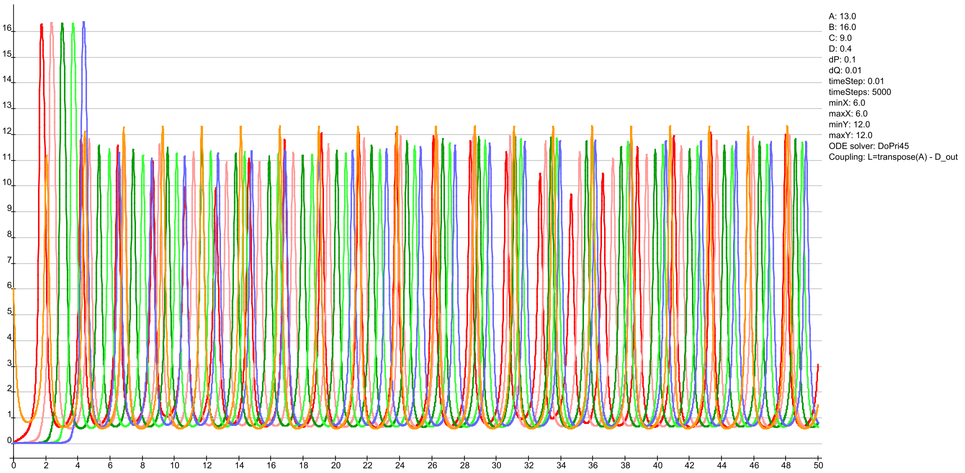

Fig AK. Functions of concentrations of the MM RD model with constant initial conditions. MM function graphs after 50 iterations. The initial conditions for node 0 are $min_X = 1$, $max_X = 1$, $min_Y = 1$, $max_Y = 1$.

GM and MM in a circuit

Non-weighted circuit

The GM and the MM models were investigated in a 3 node circuit with the feeder node 0 (Fig AL). Same sets of parameters for RD equation were used as in the chain graph. Here, the feeder 0 was initialized with parameters $V_0 = 1$ and $W_0 = 1$, whereas node 1 gets input by diffusion from the feeder node. The short delay of peaks between node 0 and node 1 can be seen in (Figs. Fig AM, Fig AN). The subsequent nodes 2 and 3 show a small delay to node 1. The phase diagrams of all 5 nodes are shown in Fig AO.

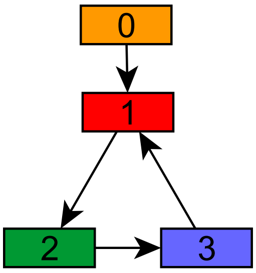

Fig AL. A test graph with a circular layout. The circuit graph with input node 0 and circular connected nodes 1-3

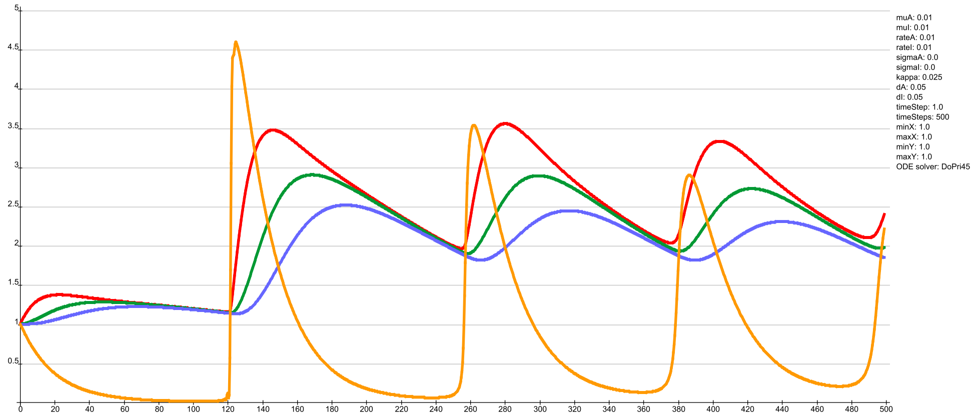

Fig AM. Functions of concentrations of the GM RD model with constant initial conditions. The GM function graph after 5000 iterations with initial conditions for node 0 $V_0 = 1$ and $W_0 = 1$ of the circular test graph (Fig AL).

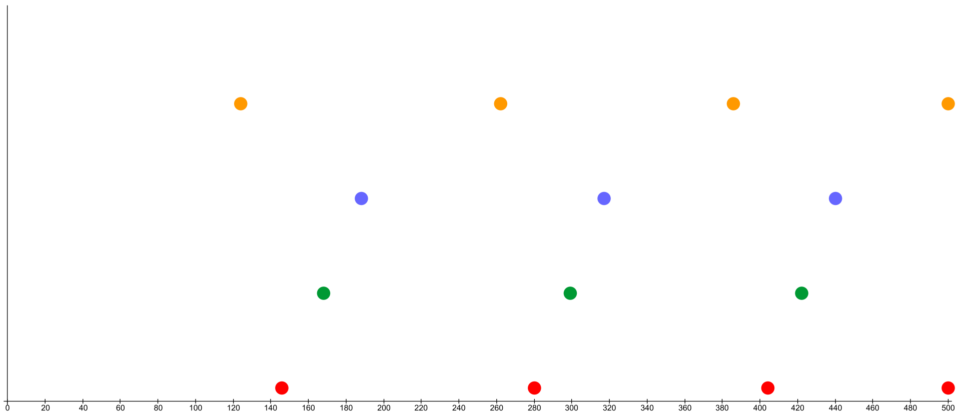

Fig AN. Visualization of peaks of functions of concentrations of the GM RD model. Peaks of the GM function graphs after 5000 iterations (Fig AM). The peaks of nodes 1, 2, 3 are delayed periodically.

The MM model shows a more regular oscillation without decrease of amplitudes (Fig AP). Node 1 is the only node in the graph which receives two inputs. This node shows a

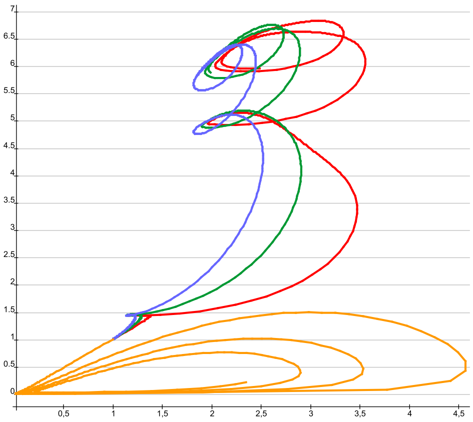

Fig AO. Functions of concentrations of the GM RD model in the phase space. The phase space of the GM function graphs (Fig AM). The different oscillations are obvious.

low-frequency overlap in its oscillations (Fig AQ) which cannot be found in node 2 and 3. Moreover, systematic delays of concentrations are generated comparable to the GM model (Fig AR). The phase diagram (Fig AS) is more regular than in the GM model (Fig AO) because a damping of oscillations is not present.

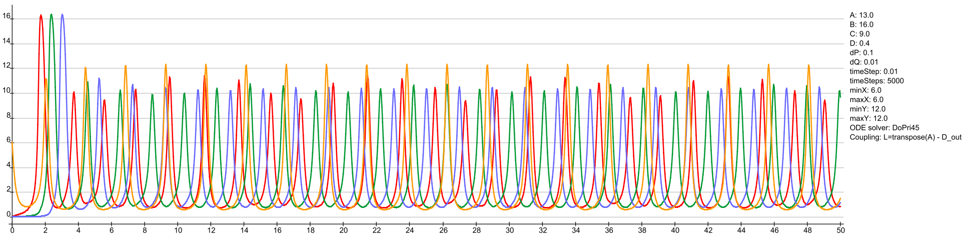

Fig AP. Functions of concentrations of the MM RD model. The MM function graph with initial conditions of node 0 of $min_X = 1$, $max_X = 1$, $min_Y = 1$, $max_Y = 1$.

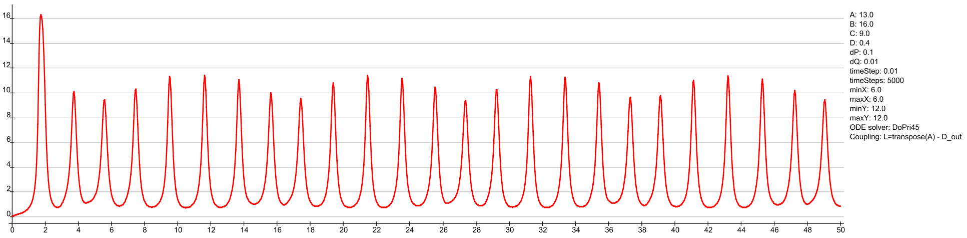

Fig AQ. Function of concentrations of node 1 of the MM RD model. The MM function of node 1 shows a low-frequency oscillation of peaks.

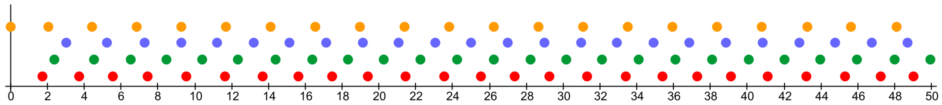

Fig AR. Peaks of the functions of concentrations of the MM RD model. The peaks of the functions of concentrations of the MM RD model are delayed like those of in the GM RD model (Fig AN).

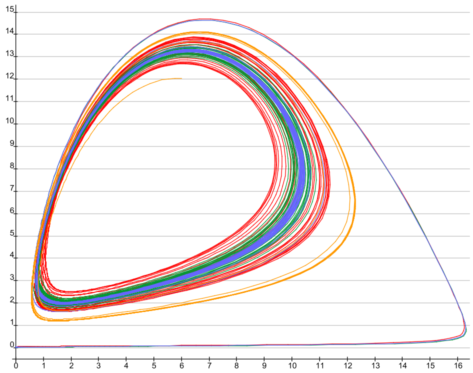

Fig AS. Functions of concentrations of the MM RD model in the phase space. The phase space of the MM RD function graphs (Fig AQ). The different oscillations are less in phase than those of the GM RD model (Fig AO).

GM RD produces patterns in a 32×32 lattice

A GM RD process was performed on a 32×32 lattice with reciprocally connected 4 neighbors (Fig AD). In a topologically ordered matrix the concentrations of each node show a discrete pattern of high and low activator concentrations (Fig AT). The inhibitor concentrations generate a stable Turing pattern as well (Fig AU). The implemented GM RD functions produce regular dot type Turing patterns that are similar to those shown above when using the same parameters (see parameter lists in Figs. Fig AT and Fig AU) and sufficient iterations (2000 iterations with 4 iterations: 8000 points of time). The four curves of concentrations are from the 4 corner nodes of the lattice and the center node (orange) (Fig AD).

Weighted circuits and decrease of weights

The weights of connections were applied to the circuit example (Fig AL) in the GM and MM models. Node 0 has the same initial conditions in the GM and MM models in the following examples. The weighted GM functions (Fig AW) show the damped oscillatory progression which is preserved by introducing connections weights to the RD process. The weighted MM functions (Fig AY) preserve their high-frequency oscillatory progression without damping and relatively constant amplitudes. Then the weight of connection from node 1 to node 2 was reduced by a factor of 10 for the GM and MM models. A relatively strong decrease of amplitudes of node 2 (green) and 3 (blue) can be seen (Fig AX). However, node 1 show brighter waves. A singular reduction of a connection weights obviously affects the pattern of oscillations of this circuit. The MM functions graph of node 1 shows a decrease of amplitude without increase or decrease of frequency (Fig AZ). Functions of node 2 and 3 appear unchanged with regard to the non-reduced connection weight circuit (Fig AZ).

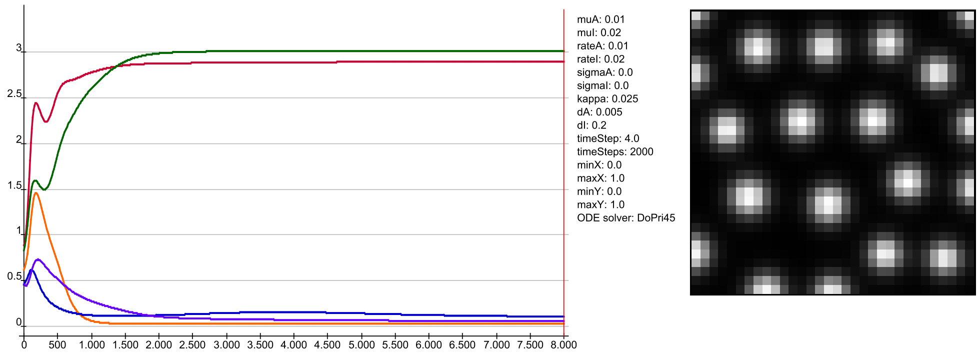

Fig AT. Pattern of concentrations of activators of a GM RD process on regular lattice. The stationary pattern of activator concentrations of a GM RD process of a 32×32 lattice (Fig AD) with reciprocally connected 4 neighbors. The output L_1 was used for diffusion.

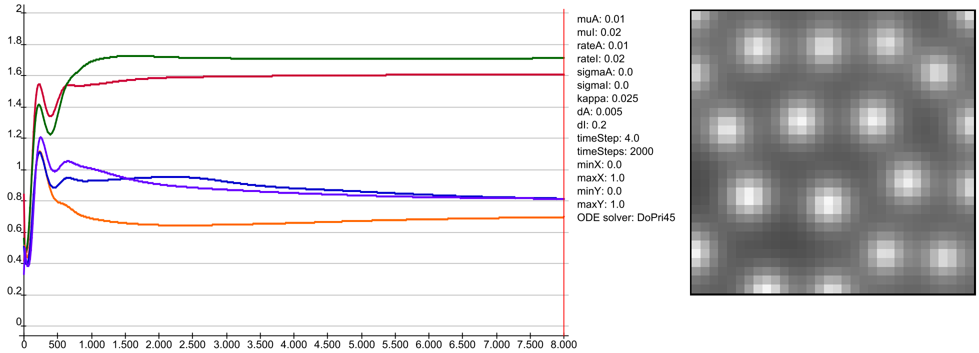

Fig AU. Pattern of concentrations of inhibitors of a GM RD process on regular lattice. The stationary pattern of inhibitor concentrations of a GM RD process of a 32×32 lattice (Fig AD) with reciprocally connected 4 neighbors. The output L_1 was used for diffusion.

Stability of MM RD with regard to stochastic noise in a subconnectome

The MM RD is relatively stable when applying additive stochastic noise to an empirical network (directed, weighted). The concentration functions are smooth without additive noise (Fig BA). When adding noise with $\sigma = 0.1$ the noise effect is obvious at the bottom of the concentration curves (Fig BB). The MM RD functions stay relatively stable when adding noise with a $\sigma = 0.5$ (Fig BC)

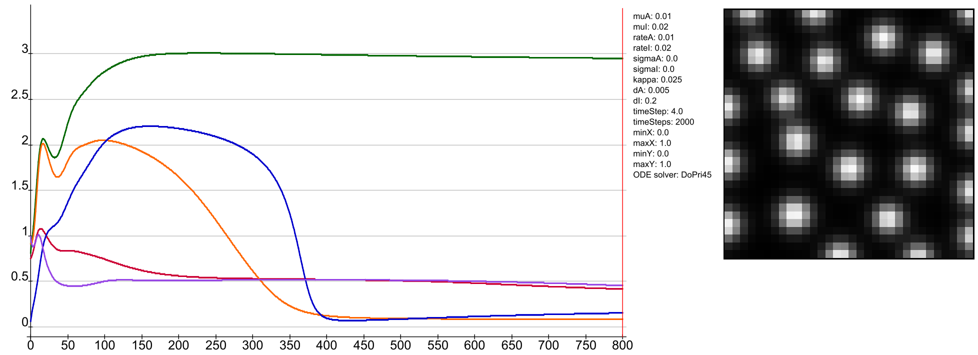

Fig AV. Pattern of concentrations of activators of a GM RD process on regular lattice with output diffusion L_3. The stationary pattern of activator concentrations of a GM RD process of a 32×32 lattice (Fig AD) with reciprocally connected 4 neighbors. The L_3 output variant of diffusion was applied.

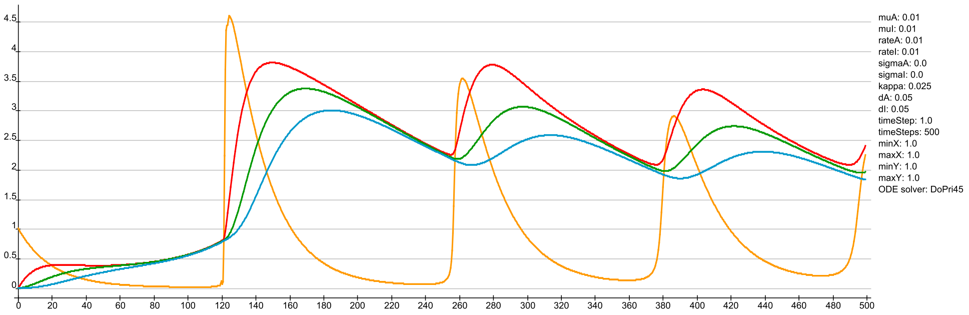

Fig AW. Functions of concentrations of the GM RD model on a weighted circular graph . The functions of concentrations of the GM RD model with initial conditions of node 0 of $V_0 = 1$ and $W_0 = 1$ (Fig AL). The weights of all connections are taken into account.

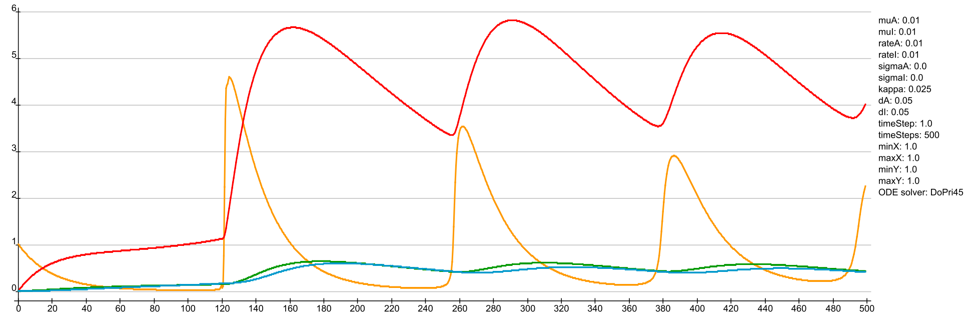

Fig AX. Functions of concentrations of the GM RD model on circular graph with weight reduction. The functions of concentrations of the GM RD model with initial conditions of node 0 of $V_0 = 1$ and $W_0 = 1$ (Fig AL). The weight of the connection from node 1 to node 2 has been reduced.

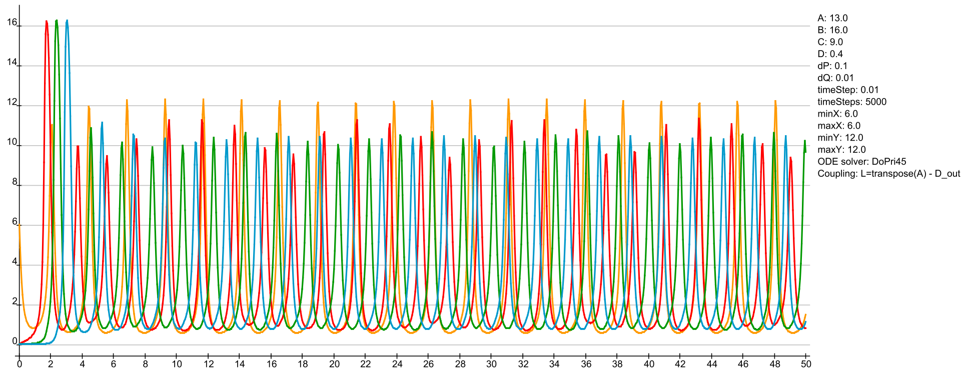

Fig AY. Functions of concentrations of the MM RD model on a weighted circular graph. The functions of concentrations of the MM RD model with initial conditions of node 0 of $min_X = 1$, $max_X = 1$, $min_Y = 1$, $max_Y = 1$ (Fig AL). The weights of all connections are considered.

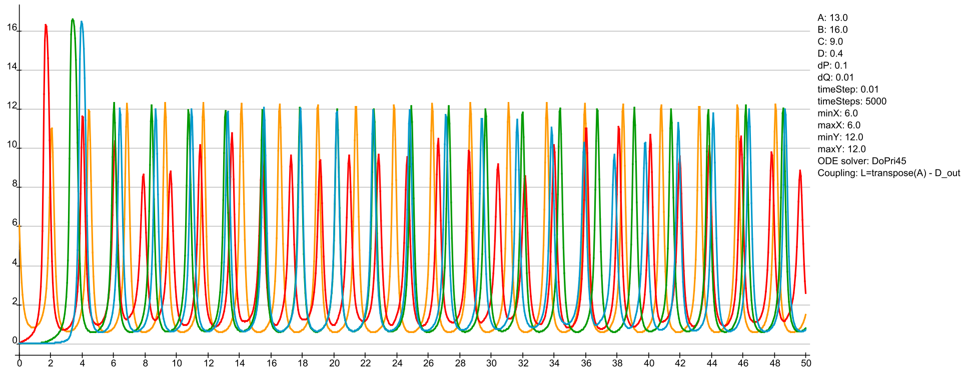

Fig AZ. Functions of concentrations of the MM RD model on a circular graph with weight reduction. The functions of concentrations of the MM RD model with initial conditions of node 0 of $min_X = 1$, $max_X = 1$, $min_Y = 1$, $max_Y = 1$ (Fig AL). The weight of the connection from node 1 to node 2 has been reduced.

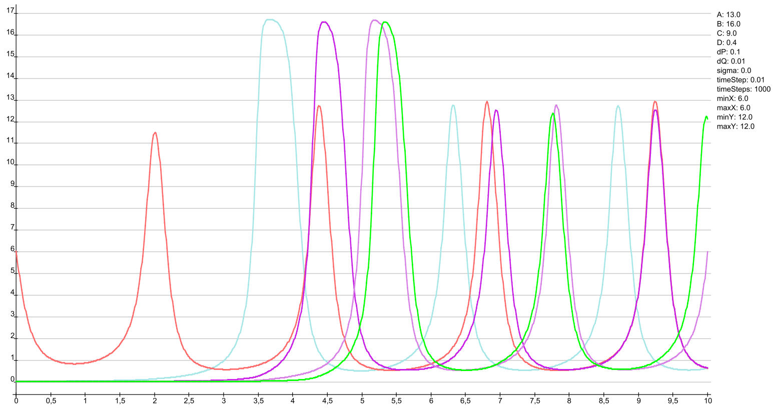

Fig BA. Functions of concentrations of the MM RD model without noise. The functions of concentration of the MM RD model in a network without noise.

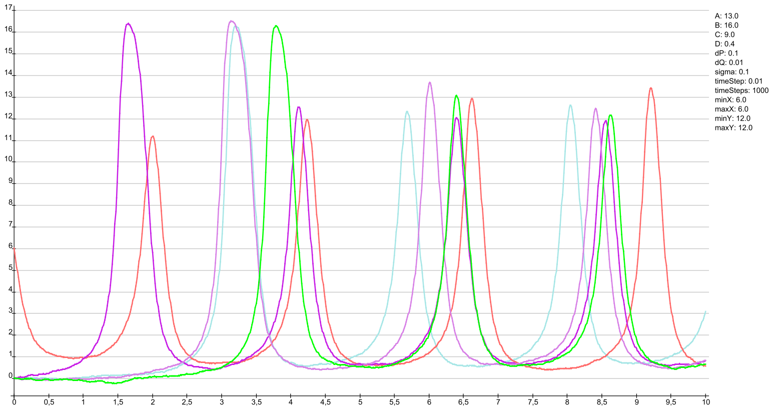

Fig BB. Functions of concentrations of the MM RD model with noise. The functions of concentration of the MM RD model in a network with stochastic noise of $\sigma = 0.1$.

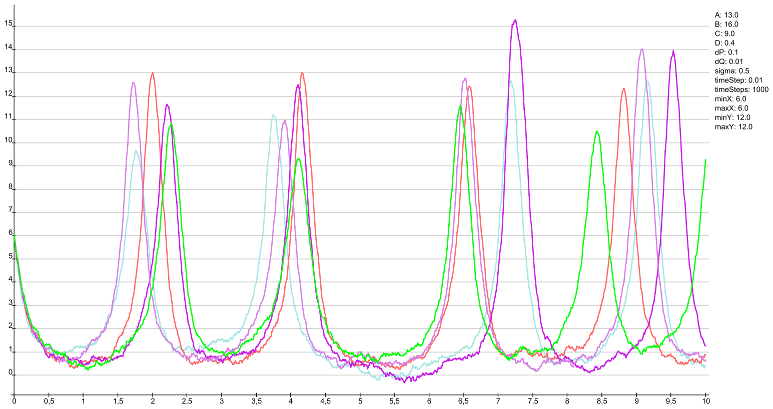

Fig BC. Functions of concentrations of the MM RD model with strong noise. The functions of concentration of the MM RD model in a network with stochastic noise of $\sigma = 0.5$.

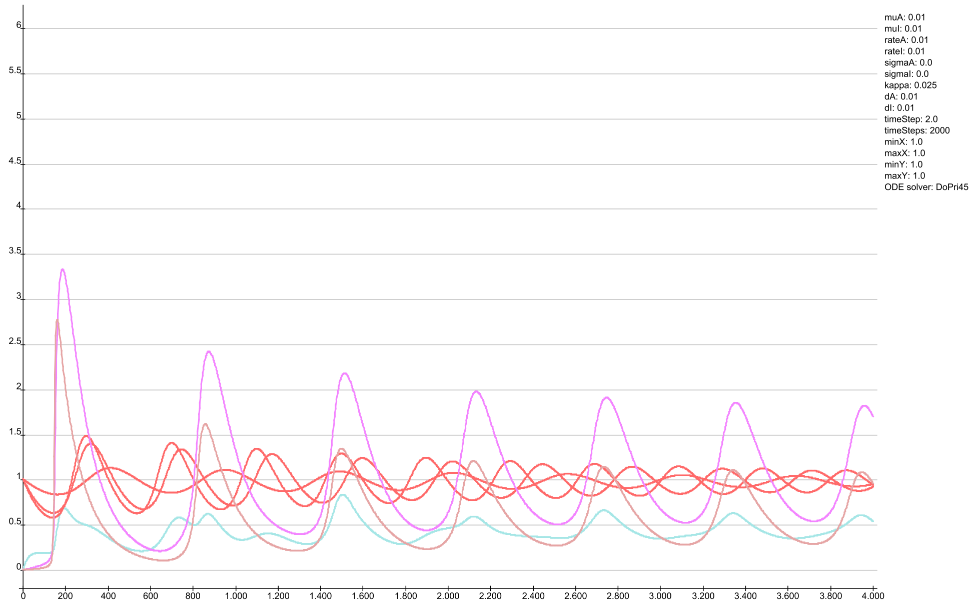

Fig BD. Functions of concentrations of activators of the GM RD model of the mechanosensitive pathway regions of the left side without weight reduction. Same initial conditions $V_0 = 1$ and $W_0 = 1$ were set for the dorsal root ganglia of the left side. Regions of interest are the same like those in Fig BK.

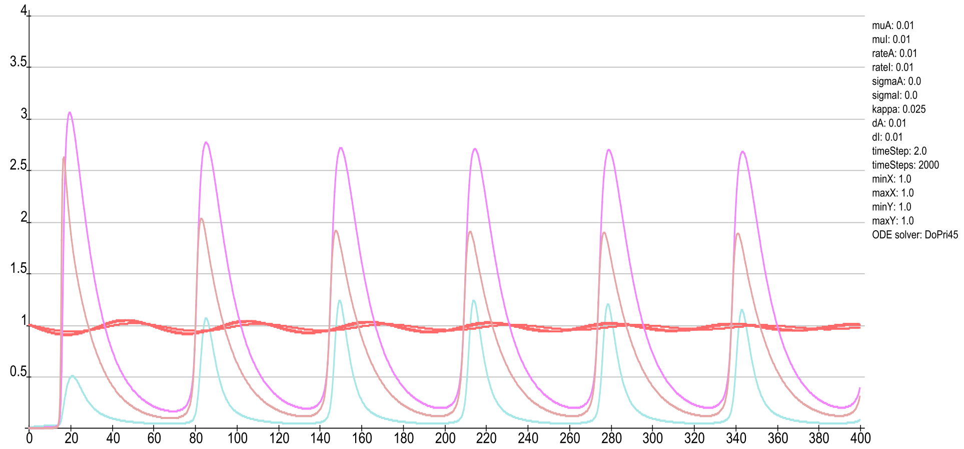

Fig BE. Functions of concentrations of activators of the GM RD model of the mechanosensitive pathway regions of the left side with constant weight reduction. Same initial conditions $V_0 = 1$ and $W_0 = 1$ were set for the dorsal root ganglia of the left side. The weight reduction has been applied to the DRG's left projection. Regions of interest are the same like those in Fig BK.

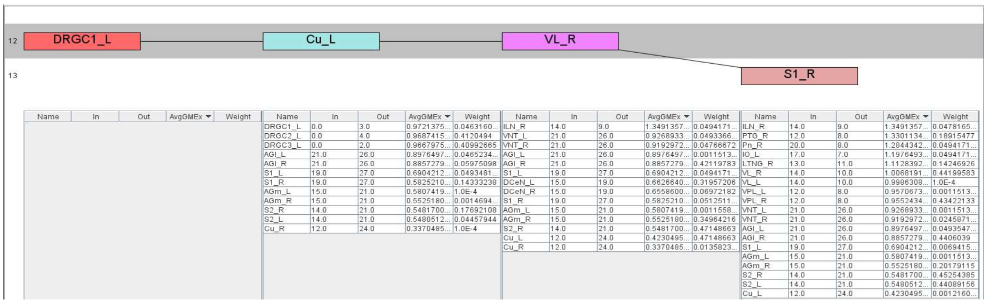

Fig BF. The average concentrations of activators of the GM RD model of input connections to regions of the mechanosensitive pathway. Input regions of the right ventrolateral thalamic nucleus were sorted for the average concentration of activators. IN: number of input connections of the bilateral mechanosensory subconnectome. OUT: number of output connections of the bilateral mechanosensory subconnectome. AvgGMEx: Average concentration of activators of the GM RD model. Weight: Logarithmic transformed weight of the region in the column "Name" to the region of the pathway. E.g., the weight of the connection from ILN_R to VL_R (pathway region) is 0.0494171..., ILN has 14 input connections within the bilateral mechanosensory subconnectome and 14 output connections.

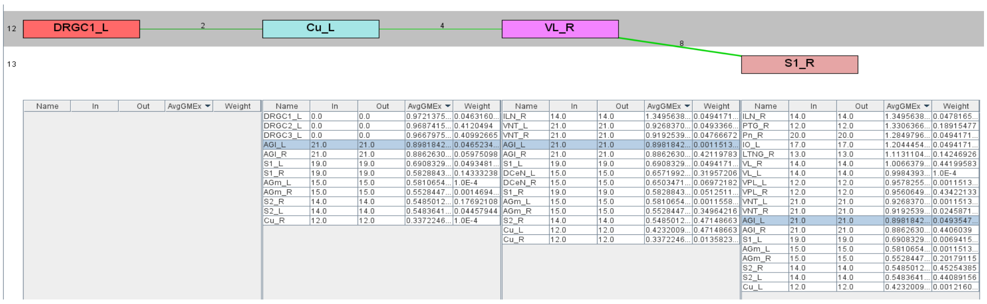

Fig BG. The average concentrations of activators of the GM RD model of input connections to regions of the mechanosensitive pathway following weight reduction. Input regions of the right ventrolateral thalamic nucleus were sorted for the average concentration of activators. Changes of ranks of regions appears for input regions of the ventrolateral thalamus and primary somatosensory cortex following the reduction of weights of the afferent cuneate nucleus. IN: number of input connections of the bilateral mechanosensory subconnectome. OUT: number of output connections of the bilateral mechanosensory subconnectome. AvgGMEx: Average concentration of activators of the GM RD model. Weight: Logarithmic transformed weight of the region in the column "Name" to the region of the pathway. E.g., the weight of the connection from ILN_R to VL_R (pathway region) is 0.0494171..., ILN has 14 input connections within the bilateral mechanosensory subconnectome and 14 output connections.

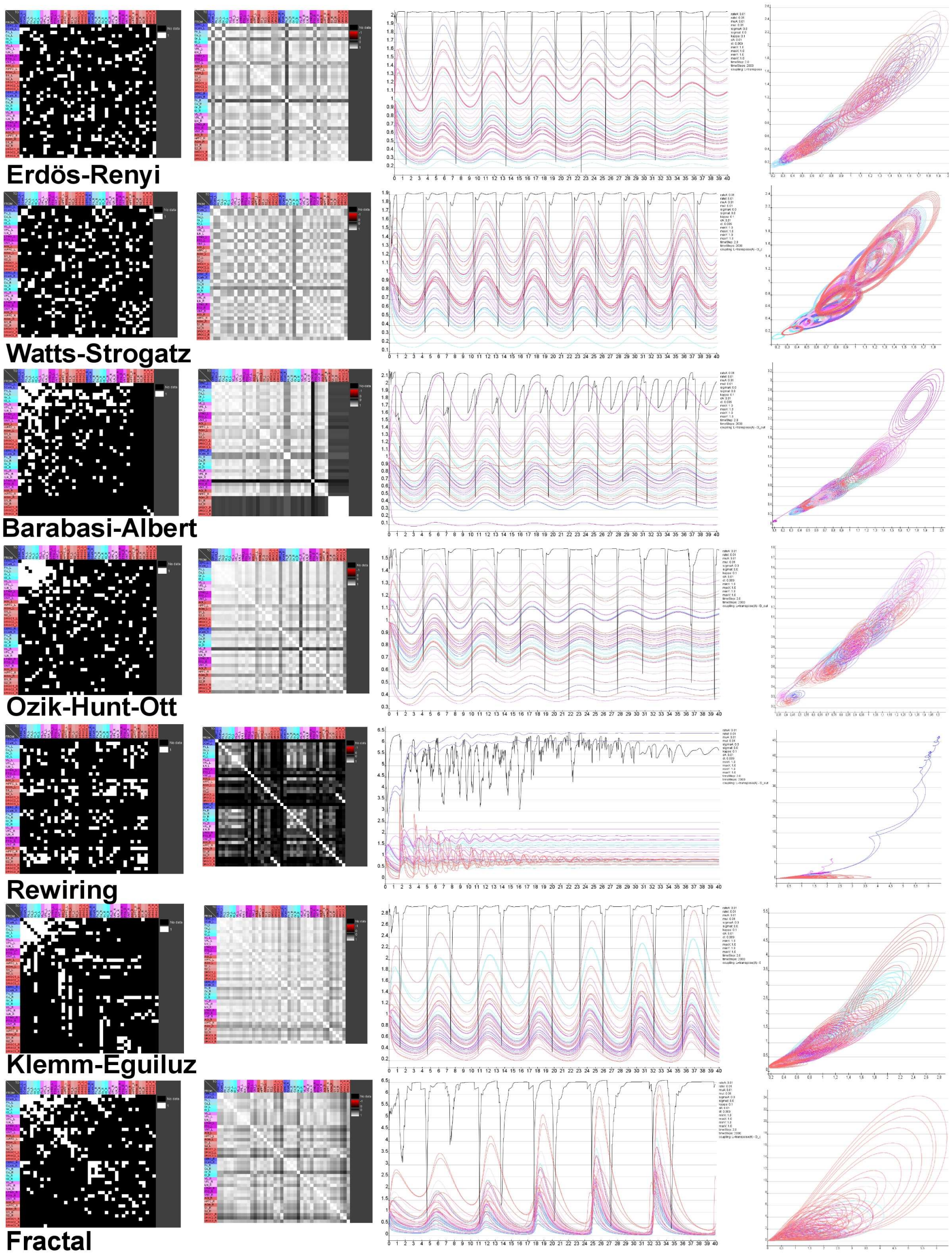

Fig BH. Comparison of GM dynamics in different degree preserving randomized networks. The left column shows the binary adjacency matrices. The second column displays the cross-correlation matrices of the GM model functions. The third column shows the course of functions with the Kuramoto index function in black. The last column contains the phase diagrams of functions.

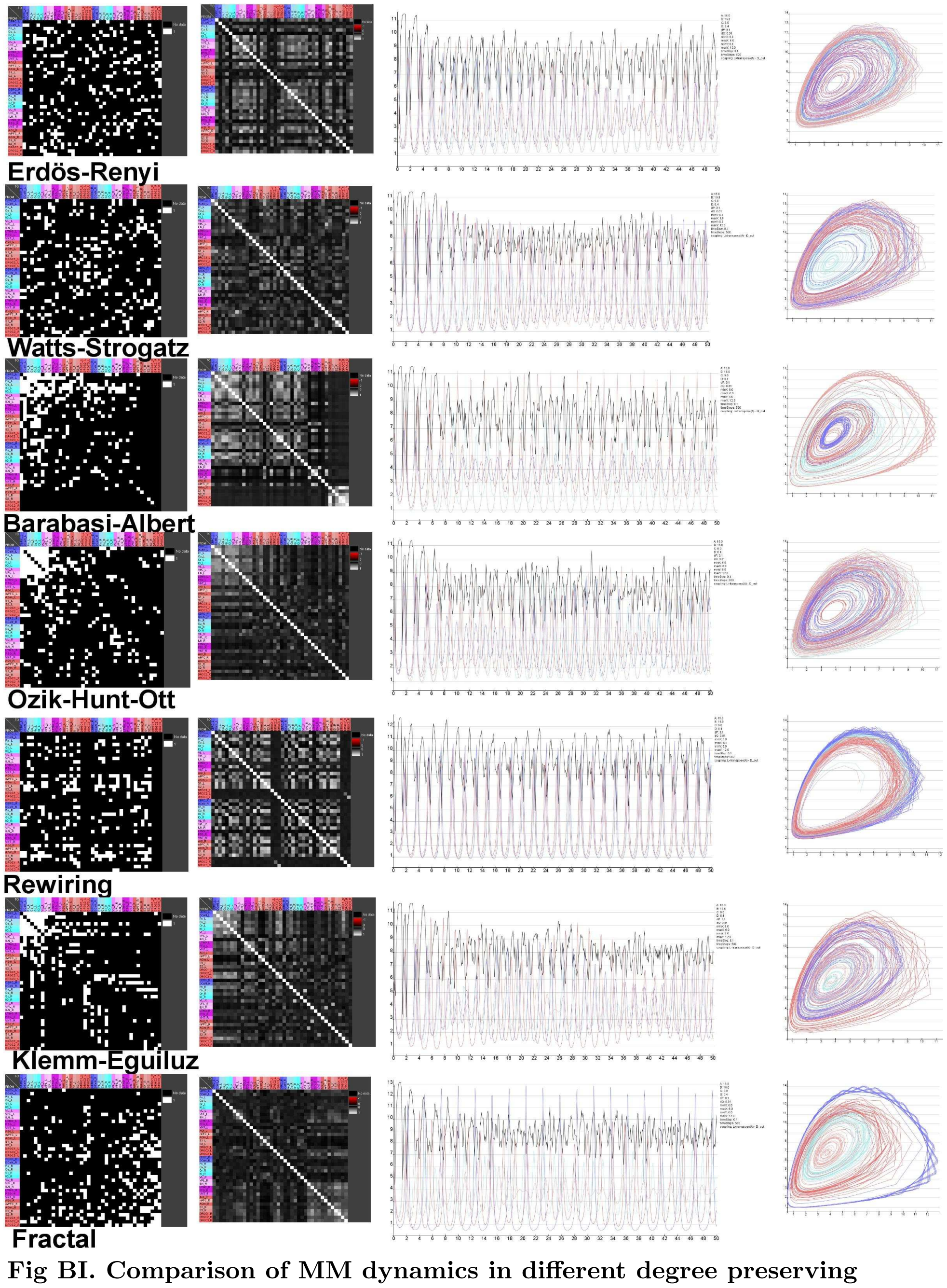

Fig BI. Comparison of MM dynamics in different degree preserving randomized networks. The left locum shows the binary adjacency matrices. The second column displays the cross-correlation matrices of the MM model functions. The third column shows the course of functions with the Kuramoto index function in black. The last column contains the phase diagrams of functions.

Supplementary figures

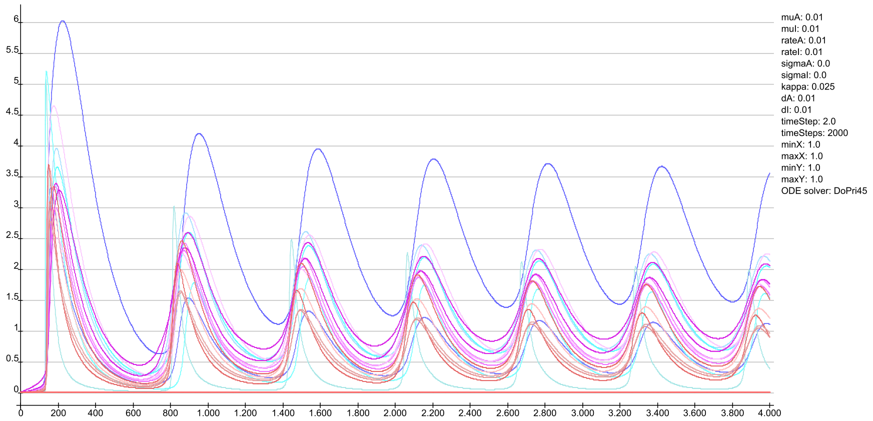

Fig BJ. Functions of concentrations for regions of right side. The functions of concentrations of right hemispheric regions are shown for the GM RD-model. Initial conditions $V_0 = 1$ and $W_0 = 1$ were set for the dorsal root ganglia of the right side.

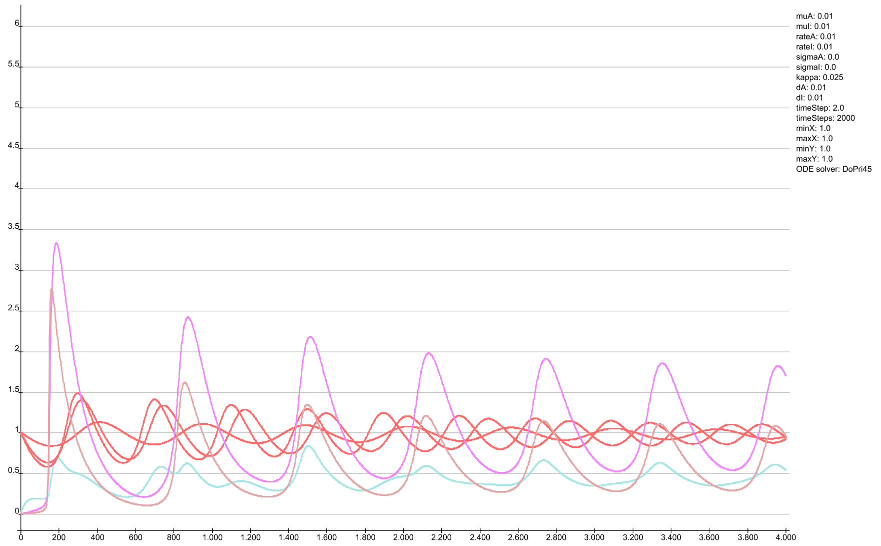

Fig BK. Functions of concentrations of the mechanosensitive pathway regions of the left side. The functions of concentrations of left hemispheric regions are shown for the GM RD-model. Initial conditions $V_0 = 1$ and $W_0 = 1$ were set for the dorsal root ganglia of the left side.

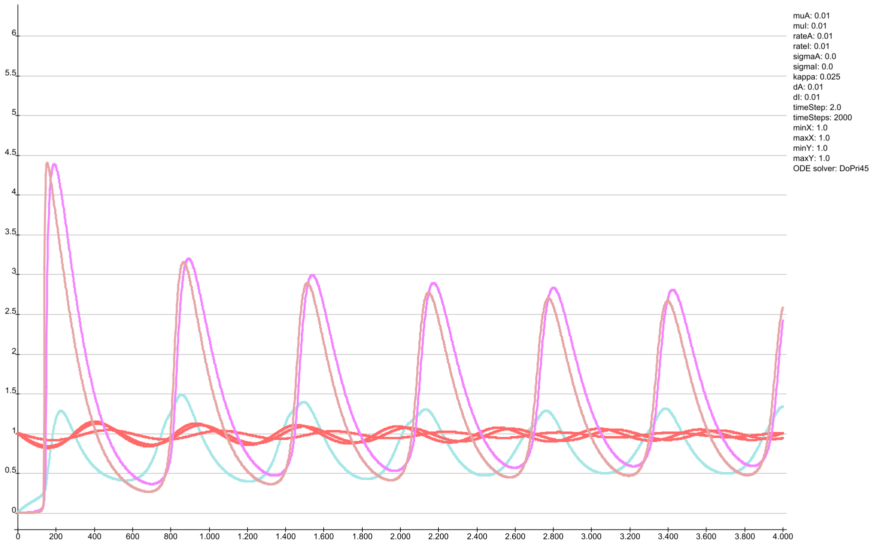

Fig BL. Functions of concentrations of the mechanosensitive pathway regions of the left side with weight reduction. The functions of concentrations of right hemispheric regions are shown for the GM RD-model following reduction of connection weights of the DRG projection to the cuneate nucleus. The decrease of amplitudes of DRG regions with regard to Fig Fig BK is obvious. Initial conditions $V_0 = 1$ and $W_0 = 1$ were set for the dorsal root ganglia of the left side.

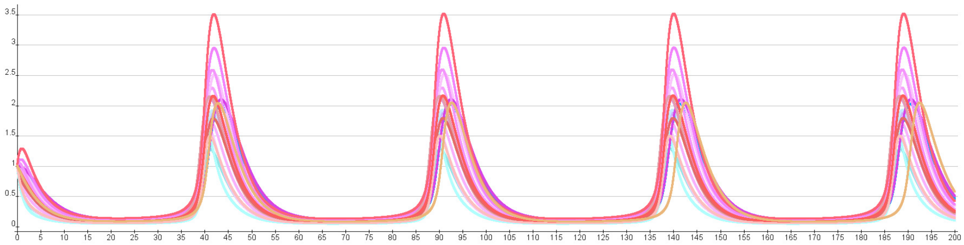

Fig BM. Oscillation of function u in a network. The function of u for $a = b = 0.01$ shows stable oscillations when coupling in a network is performed.

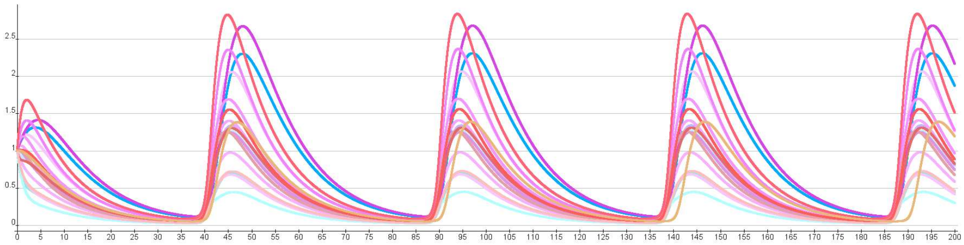

Fig BN. Oscillation of function v in a network. The function of v for $a = b = 0.01$ shows stable oscillations when coupling in a network is performed.

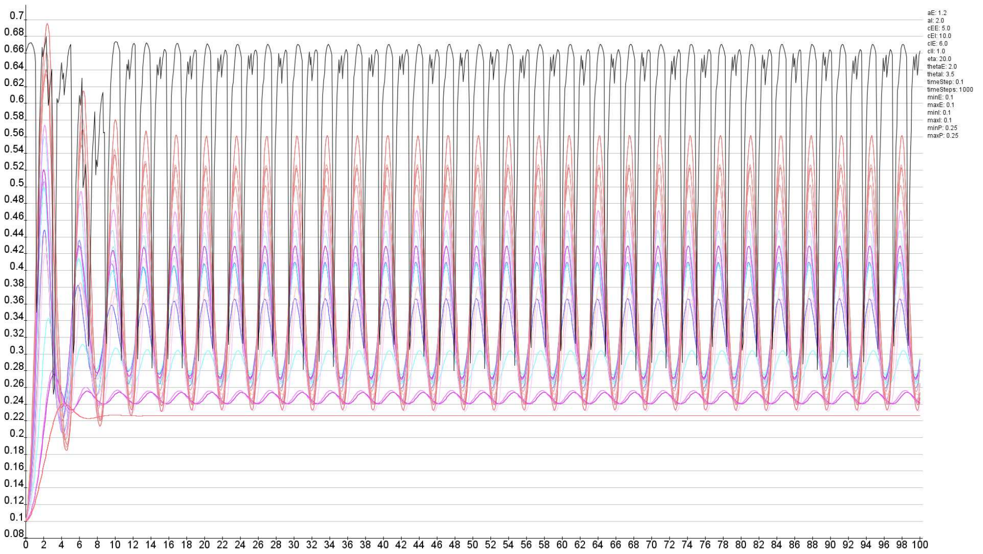

Fig BO. Wilson-Cowan neural mass model. The Wilson-Cowan model was applied to the network shown in 2 and which was used for GM and MM analysis as well. The functions of regions are displayed in the corresponding region color. The Kuramoto index is the black curve.

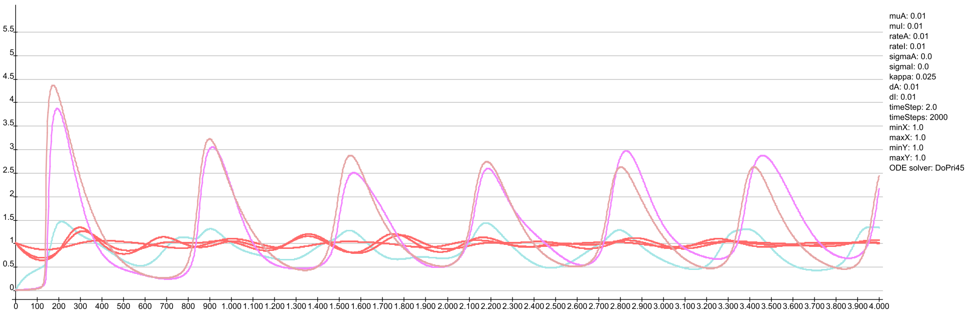

Fig BP. Functions of concentrations of the mechanosensitive pathway regions of the left side with weight reduction. Same initial conditions $V_0 = 1$ and $W_0 = 1$ were set for the dorsal root ganglia of the left side. The weight modulation function has been applied to the DRG's left projection. Regions of interest are the same like those in Fig BK. The result can be directly compared with the functions of concentrations of the same regions following a constant weight reduction (Fig BL).

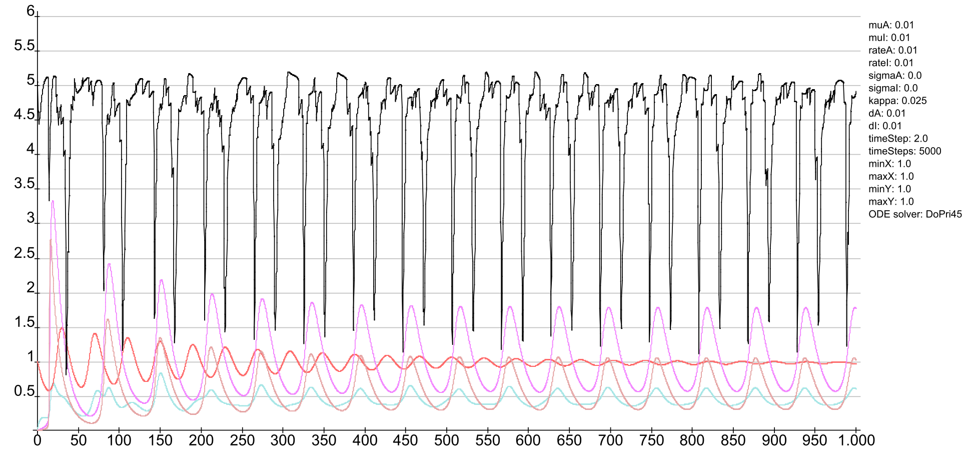

Fig BQ. GM RD-model without weight reduction. GM RD-model has been computed 10000 iterations. Initial conditions $V_0 = 1$ and $W_0 = 1$ were set for the dorsal root ganglia 1-3 of the left side. The x-axis values are to be multiplied by 10, since a total of 10000 iterations were performed. The black curve indicates the Kuramoto order parameter.

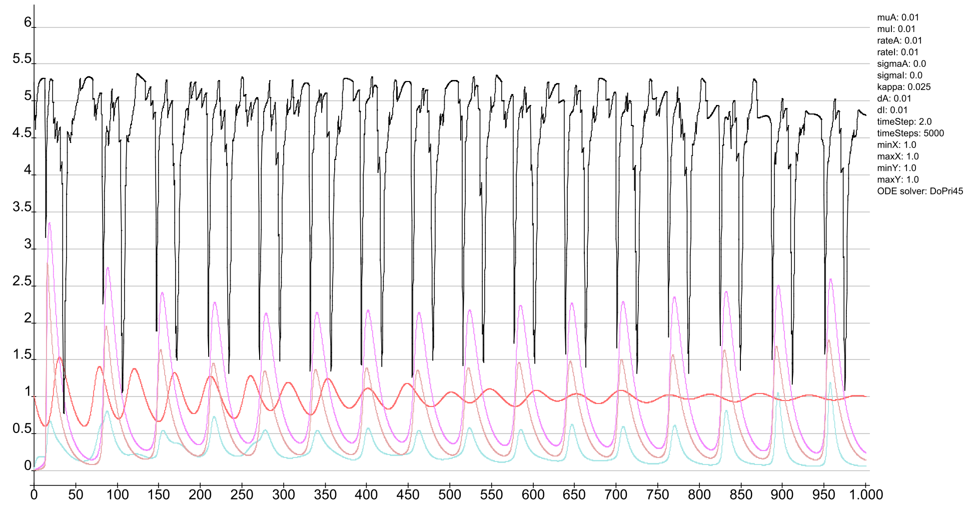

Fig BR. GM RD-model with weight reduction. The weight modulation function (damped cosine function) was applied to the weights of the DRG 1-3 projections to the cuneate nucleus of the left side. Initial conditions $V_0 = 1$ and $W_0 = 1$ were set for the dorsal root ganglia 1-3 of the left side. The x-axis values are to be multiplied by 10, since a total of 10000 iterations were performed. The black curve indicates the Kuramoto order parameter.

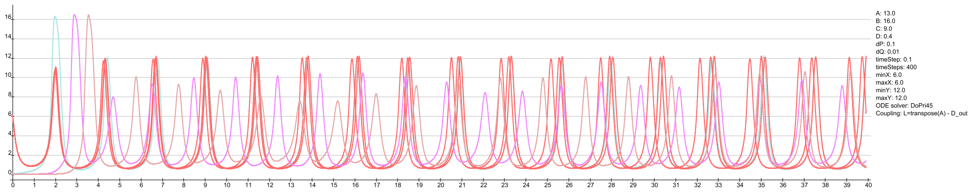

Fig BS. Functions of concentrations of the MM RD of the regions of the left side. The MM concentration functions of same ROIs like those in GM RD-modeling (3). Initial conditions $P_0 = 6$ and $Q_0 = 12$ were set for the three dorsal root ganglia of the left side.

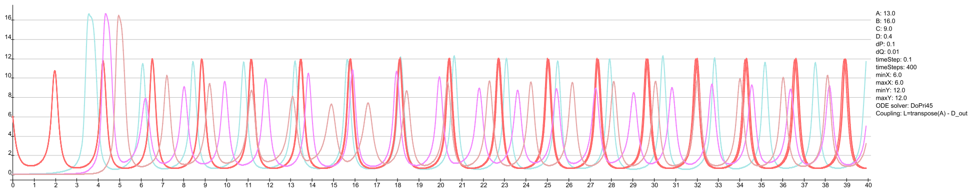

Fig BT. Functions of concentrations of the MM RD of the regions of the left side with weight reduction. Same initial conditions $P_0 = 6$ and $Q_0 = 12$ were set for the dorsal root ganglia of the left side. The same constant weight reduction has been applied to the DRG's left projection and network as that in Fig BK. In contrast to Fig BK the functions of concentrations of all regions were plotted.

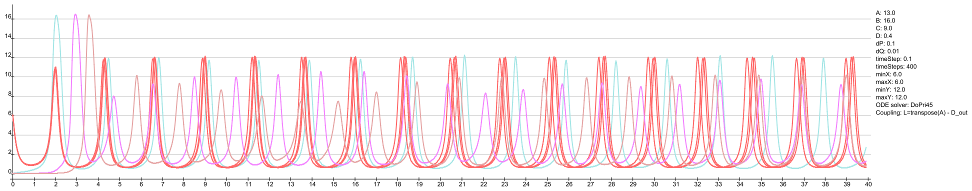

Fig BU. Functions of concentrations of the MM RD of the regions of the left side with dynamic weight reduction. Same initial conditions $P_0 = 6$ and $Q_0 = 12$ were set for the three dorsal root ganglia of the left side. The weight modulation function (21) has been applied to the DRG's left projection. Regions of interest are the same like those in Fig BK.

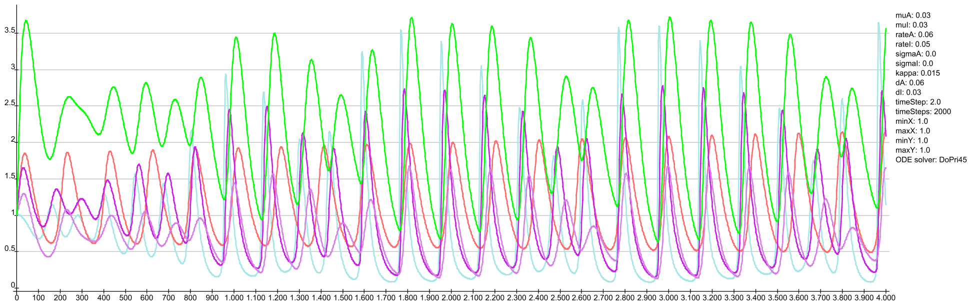

Fig BV. GM RD process on the mechanosensitive subnetwork. The result of a GM RD process on the mechanosensitive subnetwork without distance conditions is shown by concentration functions. Color codes of functions are the same as regions colors in 22.

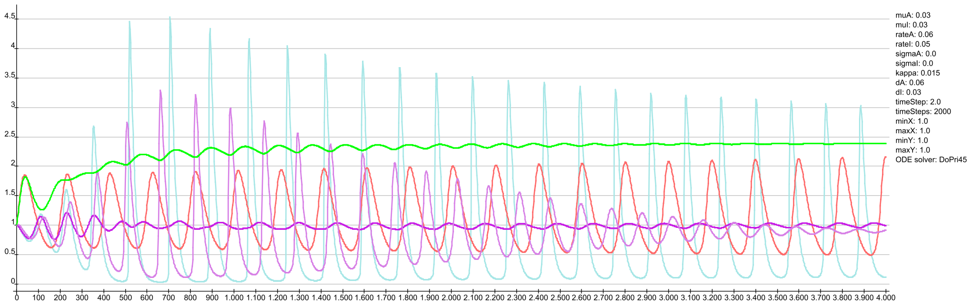

Fig BW. GM RD process on the mechanosensitive subnetwork with the distance condition. The result of a GM RD process on the mechanosensitive subnetwork with application of the distance conditions is visualized by concentration functions.
